# Phylodynamics of Influenza A/H1N1pdm09 in India Reveals Circulation Patterns and Increased Selection for Clade 6b Residues and Other High Mortality Mutants

**DOI:** 10.3390/v11090791

**Published:** 2019-08-27

**Authors:** Dillon C. Adam, Matthew Scotch, C Raina. MacIntyre

**Affiliations:** 1 Biosecurity Program, The Kirby Institute, Faculty of Medicine, University of New South Wales, Sydney, NSW 2052, Australia; 2 Biodesign Center for Environmental Health Engineering, Biodesign Institute, Arizona State University, Tempe, AZ 85287, USA; 3 College of Health Solutions, Arizona State University, Phoenix, AZ 85004, USA; 4 College of Public Service & Community Solutions, Arizona State University, Tempe, AZ 85004, USA

**Keywords:** Influenza, public health, phylogenetics, India

## Abstract

The clinical severity and observed case fatality ratio of influenza A/H1N1pdm09 in India, particularly in 2015 and 2017 far exceeds current global estimates. Reasons for these frequent and severe epidemic waves remain unclear. We used Bayesian phylodynamic methods to uncover possible genetic explanations for this, while also identifying the transmission dynamics of A/H1N1pdm09 between 2009 and 2017 to inform future public health interventions. We reveal a disproportionate selection at haemagglutinin residue positions associated with increased morbidity and mortality in India such as position 222 and clade 6B characteristic residues, relative to equivalent isolates circulating globally. We also identify for the first time, increased selection at position 186 as potentially explaining the severity of recent A/H1N1pdm09 epidemics in India. We reveal national routes of A/H1N1pdm09 transmission, identifying Maharashtra as the most important state for the spread throughout India, while quantifying climactic, ecological, and transport factors as drivers of within-country transmission. Together these results have important implications for future A/H1N1pdm09 surveillance and control within India, but also for epidemic and pandemic risk prediction around the world.

## 1. Introduction

In early 2009, a novel influenza A H1N1 (A/H1N1pdm09) virus emerged in Veracruz, Mexico and California, USA and was responsible for the first influenza pandemic of the 21st century [1]. As a triple-reassortant influenza virus antigenically distinct from the former seasonal A/H1N1 [2], the virus quickly spread around the world causing severe perturbations to health and surveillance systems [3,4]. During the pandemic, estimated case fatality ratios (CFR) ranged from less than 0.001% to 10% due in part to significant case under-ascertainment and the heterogeneity of case definitions between countries [5]. However, a true geographic variation in CFR could not be excluded [6]. Furthermore, as diagnostic capacity was overwhelmed, laboratory confirmation of infection was largely restricted to severe and fatal cases leaving estimates of total morbidity unknown. Studies have since estimated approximately 24% (95% confidence interval: 20–27%) of the global population was infected during the pandemic [7], while mortality was similar to that of a severe seasonal epidemic (~0.01% CFR) [8]. Consistent with past pandemics, A/H1N1pdm09 has continued to circulate around the world seasonally every year since 2009, replacing pre-pandemic A/H1N1 strains [9,10], and co-circulating with influenza A/H3N2 and influenza B viruses [11,12].

The first case of A/H1N1pdm09 in India was reported in May 2009 in the city of Hyderabad, Telangana [13]. By December, widespread human-to-human transmission led to substantial morbidity and mortality within the country [13,14]. Following a second epidemic wave in early 2010, approximately 50,000 cases had been reported in India during the pandemic period with a reported CFR of 6.1% [15]. In subsequent seasons, the annual incidence of H1N1pdm09 in India was low, with approximately 5000 cases or less reported nationally each year. However, in 2015 and 2017, widespread epidemics occurred with an estimated 43,000 and 39,000 cases reported, as well as approximately 3000 and 2300 deaths respectively [16]. States particularly affected included Maharashtra, Gujarat, Rajasthan and Madhya Pradesh, accounting for 75.6% (*n* = 2261/2991), and 72.1% (*n* = 1634/2266) of all deaths nationally in 2015 and 2017. In some states, CFR of up 20% in 2015 and 30% in 2017 have been reported. Reasons for these frequent and severe epidemic waves of A/H1N1pdm09 in India with apparent high mortality remain unclear. Resource-constrained lower-middle income countries such as India where access to quality health care might be limited have been associated with excess influenza mortality [6,8,17], however ongoing reports of A/H1N1pdm09 associated mortality among otherwise healthy adults aged under 65 years in India remains particularly unusual [18,19,20].

Emerging methods of data integration in Bayesian phylogenetics have provided new insights into the evolution and dynamics of influenza A viruses [21,22,23], however the use of these methods have yet to be applied to A/H1N1pdm09 in India. In this study, we aim to utilize these methods to explore possible genetic explanations for the high severity and mortality of A/H1N1pdm09 in India. We also aim to understand the temporal, population and transmission dynamics of A/H1N1pdm09 in India to estimate potential case under-ascertainment and opportunities for outbreak control. Our results have potential implications for predicting the future risk of influenza A/H1N1pdm09 severity and spread, both within India and around the world.

## 2. Materials and Methods

### 2.1. Compilation of Sequence Datasets

We searched the Global Initiative for the Sharing All Influenza Data (GISAID) on 28 March 2018 for all available haemagglutinin (HA) gene sequences sampled in India between 2009 and 2017 inclusive [24]. We identified 930 out of 1025 openly available sequences with collection date and location metadata publicly available or available upon request from the uploading authors (Acknowledgment Table 1). Of those, we considered only 625 to be of sufficient length for analysis (>1600 bp). We removed 12 sequences across five State and Union Territories (S/UT) of India due to low sampling frequencies defined as less than two sequences per 10 million population within the study period. This cut-off was selected through trial and error with the purpose of maintaining sufficient sampling across the study period, without excessively removing valuable data-points (sequences and locations). We aligned the final dataset of 613 sequences using MUSCLE v.3.8 [25] in Geneious v10.1.2 [26] and manually inspected and trimmed the HA coding regions for further analysis. Table 1 and Supplementary Figure S1 shows the spatial and temporal distribution of the Indian sequence dataset. For comparative analyses with globally circulating sequences we searched GISAID for all full length (>1600 bp) A/H1N1pdm09 HA sequences sampled during the same period with complete region and date of sampling metadata. Excluding India, we identified 23,144 records. We removed 1935 records with duplicate isolate sources resulting in a final global dataset of 21,209 sequences aggregated to one of ten regions roughly similar to a previous study [11]: Northern Asia (Mongolia and Russia), China, Japan/Korea, Southern Asia/South-East Asia, Middle East/Western Asia, Africa, Europe, North America (Central America and USA/Canada), South America, and Oceania. The spatial and temporal distribution of the complete global A/H1N1pdm09 sequence dataset can be seen in Supplementary Table S1. To reduce computational burden and limit the impact of sampling bias we created two independent sequence subsets (S1 and S2), randomly sampling up to 50 sequences per region-year as per previous studies [11,12]. Each subset was aligned using MAFFT v1.3.7 [27] in Geneious v10.1.2 [26] and manually inspected and trimmed as before.

### 2.2. A/H1N1pdm09 Transmission within India

We used BEAUti v1.10 [28] to specify a discrete-trait phylogeographic model to estimate all possible transitions between the 14 Indian S/UT included in our Indian dataset (*n* = 613). We selected a GTR+Γ_4_ substitution model with a relaxed clock based on preliminary path sampling and stone-stepping sampling results [29,30] (Supplementary Table S2) and correlation (R^2^ = 0.81) between the sampling time and root-to-tip divergence of HA [31] (Supplementary Figure S2). We ran four models independently with 50 million Markov chain Monte Carlo (MCMC) generations sampling every 5000 steps. We inspected runs for similar convergence around the posterior using Tracer v1.6 [32]. We combined combined runs using LogCombiner v1.10 [28] after inspecting for similar convergence around the posterior using Tracer v1.6 removing 10% each as burn-in [32]. Each model specified a nonparametric Bayesian Skygrid tree prior [33,34] with 50 intervals as default to reconstruct past population demographics. We produced maximum clade credibility (MCC) trees from the combined posterior tree distribution using TreeAnnotator v1.10 specifying median node heights [28]. We used SpreaD3 v0.9.6 [35] to calculate Bayes factors (BF) for each pairwise transition between the 14 S/UT as well as to geospatially render the phylogeographic projections. We defined sufficient support for transmission as a BF > 3 as per convention [36]. Higher levels of statistical support were defined according to Supplementary Table S3 [37].

A single state, Maharashtra, accounted for 39.5% (*n* = 242/613) of the complete India dataset. To compare and control for potential sampling in the dataset, we generated five unique subsets, randomly sampling up to five sequences per location-year in location-years with greater than five sequences, while leaving those with less than five sequences per location-year untouched. This created a more equitable spatiotemporal distribution of sequences without removing under-represented location-years (Supplementary Table S4). For each of the five random subsets, we specified an identical discrete-trait phylogeographic model as above. We calculated BF for each pairwise transition as the average between all five subsets using SpreaD3 v0.9.6 [35].

### 2.3. Predictors of Transmission within India

We extended the phylogeographic model above using a generalized linear modelling (GLM) framework to investigate the contribution of climactic, ecological, and demographic factors as potential predictors of transmission [23]. Based on previously published studies [38,39], preliminary ecological factors of interest included longitude, latitude, average temperature, and average precipitation. Preliminary demographic predictors included average population growth, population density, gross domestic state product (GDSP), and the percentage of S/UT population living in urban environments. We also calculated aviation passenger flux as the average number of domestic passengers recorded in all S/UT between 2009 and 2017 using data publicly available from the Indian Directorate General of Civil Aviation [40]. After assessing for collinearity, the final GLM included S/UT population density, average temperature, longitude, and passenger flux. We also included great-circle-distance between each pair-wise location and sample size by S/UT to assess for potential sampling biases. We estimated the mean posterior probability of each predictor’s inclusion in the model, BF, and the effect coefficient using R as per [36,41].

### 2.4. Positive Selection Analysis

For the complete Indian sequence dataset (*n* = 613) we generated rate ratios of non-synonymous to synonymous (d_N_/d_S_) mutations using Bayesian renaissance counting (BRC) implemented in BEAST v1.10 [28] assuming three independent (1, 2, 3) codon partitions [42]. At each site, positive selection was determined where the lower 95% Bayesian credible interval (BCI) was greater than one (d_N_/d_S_ > 1). Potential sites under selection were validated using both two-rate fixed effects likelihood (FEL) and single-likelihood ancestor counting (SLAC) tests in HyPhy assuming a p-value threshold of 0.1 which is convention [43,44]. For this, fixed MCC trees were generated with TreeAnnotator v1.10 [28] from previous BEAST outputs. We also used BRC methods in BEAST v1.10 to compare d_N_/d_S_ ratios at significant sites in both global A/H1N1pdm09 datasets previously generated (S1 and S2). Due to the significant number of taxa in both global datasets (2 × *n* = 4063), we first generated a posterior distribution of 10,000 trees each at 100 million MCMC generations before inferring d_N_/d_S_ ratios on a fixed posterior of 500 trees after removing 50% burn-in similar to Bedford et al. 2015 [11]. For all three datasets, relative H1 and H3 site numbering was determined using the influenza research database’s (FluDB) HA Subtype Numbering algorithm [45] and residue frequencies at selected sites was determined using Geneious v10.1.2 [26]. Selected sites within the HA structure (RCSB PDB ID: 4LXV [46]) were visualized with YASARA View v19.7.20 [47].

## 3. Results

### 3.1. CFR and Viral Population Demographics

**Figure 1:**
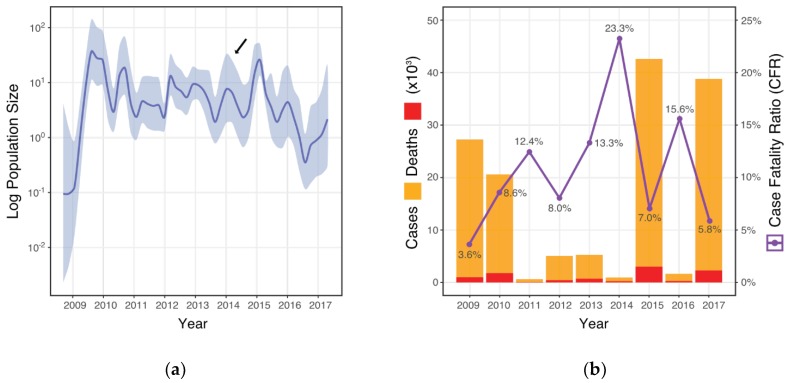
(**a**) Bayesian Skygrid estimation of effective viral population size (N_e_) of A/H1N1pdm09 in India between 2009 and 2017. Here, we show the mean N_e_ and respective 95% Bayesian credible interval (BCI) plotted in blue. (**b**) Official A/H1N1pdm09 case and death counts (left axis) reported by the National Centre for Disease Control in Delhi (NCDC) [15,16]. Calculated yearly case fatality ratios (CFR) with corresponding percentages shown (right axis). The arrow in Figure 1a points to the inferred seasonal epidemic in 2013/14, in contrast to reported cases in Figure 1b.

In Figure 1a we show the inferred past population demographics of A/H1N1pdm09 in India between 2009 and 2017 alongside the observed cases and deaths recorded each year [15,16], and calculated the CFR (Figure 1b). The largest epidemic peak inferred as the effective population size (N_e_) of A/H1N1pdm09 (Figure 1a) can be seen in 2009 (Median N_e_ = 36.24; 95 BCI = 11.93 to 152.25) concurrent with the global pandemic at the time. The second largest peak N_e_ can be seen in early 2015 (Median N_e_ = 25.58; 95 BCI = 13.74 to 54.90) with epidemic activity estimated to have begun in late 2014 (Figure 1a). A low N_e_ is seen in mid-2011 (Median N_e_ = 12.60; 95 BCI = 4.20 to 45.10) consistent with the observed case data (Figure 1b) before consecutive N_e_ waves of similar height can be seen in the years 2012 to 2014. Less than 1000 cases of A/H1N1pmd09 were reported nationally in 2014 (Figure 1b), however results of the demographic reconstruction suggest a typical seasonal epidemic beginning in late 2013 before declining in mid-2014 (Figure 1a Arrow). A widening 95% BCI can be seen in mid-2016 onward likely due to the reduction in taxa available for accurate demographic inference (*n* = 28).

### 3.2. d_N_/d_S_ Selection Analysis and Amino Acid Variations

**Figure 2:**
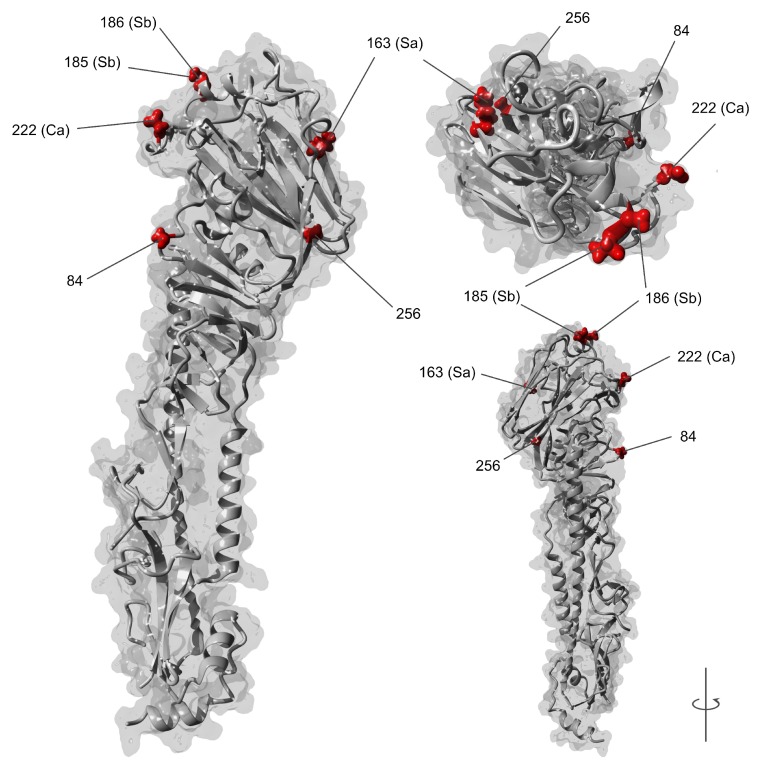
Relative structural locations of A/H1N1pdm09 residues within the HA monomer under positive selection in India. Sites under selection are highlighted in red and numbered without signal peptide (H1 numbering). Letters in parentheses indicate sites located within the known antigenic domains of HA1. (RCSB PDB ID: 4LXV).

Bayesian renaissance counting (BRC) identified 40 codon sites under positive selection given the uncertainty of the posterior phylogeny (95% BCI > 1.0). Six of these sites were also detected by both FEL and SLAC procedures assuming a fixed tree (Table 2). Site 222 (H1 numbering onwards) had the highest d_N_/d_S_ ratio (13.42) followed by site 84 (8.14), 185 (4.50), and 256 (4.39). All validated sites detected in the complete Indian dataset, excluding 163, exhibited significantly higher d_N_/d_S_ ratios given the 95% BCI determined by BRC compared to both international samples. Among the six selected sites, four (163, 185, 186 and 222) are located within the antigenic sites of HA (Ca, Cb, Sa, Sb). The relative structural positions of these six selected sites are visualized in Figure 2. The complete d_N_/d_S_ results for both the Indian and International sequence datasets can be seen in Supplementary Tables S5 and S6.

The frequency of residue changes (*Datafile 1*) at each detected site in India varied. Among the detected sites, S185T was the most frequently observed residue change (*n* = 473/613; 77.2%), followed by K163Q (*n* = 240/613; 39.2%), A256T (*n* = 231/613; 37.7%), and S84N (*n* = 160/613; 26.1%). Two residue changes, D222G and D222N, were observed in 3.1% (*n* = 19/613) and 1.6% (*n* = 10/613) of the Indian sample respectively. Four residue changes could be observed at site 84; in descending order of frequency: S84N (*n* = 160/613; 26.1%), S84G (*n* = 66/613; 10.8%), S84I (*n* = 6/613; 1.0%) and S84D (*n* = 2/613; 0.3%). Eight discrete changes were observed at selected site 186; in descending order of frequency: A186T (*n* = 5/613; 0.82%), A186G (*n* = 2/613; 0.33%), and A186V (*n* = 1/613; 0.16%).

### 3.3. Phylogeography of A/H1N1pdm09 

**Figure 3:**
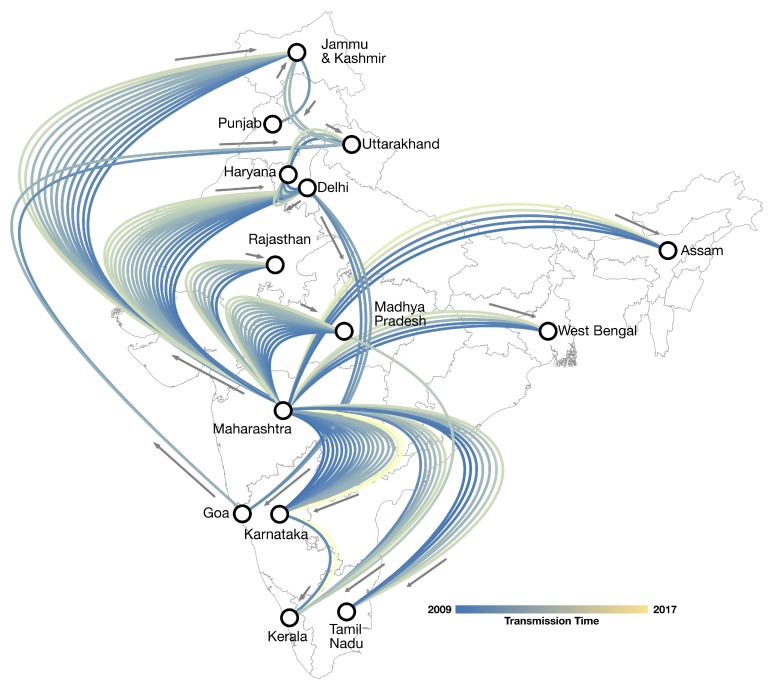
Phylogeography of definitive A/H1N1pdm09 transmission between S/UT in India since 2009. Paths between S/UT are coloured by inferred transmission time and directions indicated by adjacent arrows. All routes shown are characterised by definitive statistical support (Bayes factor (BF) > 100). *Supported* to *very strongly supported* routes (100 > BF > 3) are not shown but can be seen in Supplementary Table S8.

In Figure 3 we show the spatio-temporal projection of definitive A/H1N1pdm09 transmission (BF > 100) in India from 2009 to 2017 based on the most complete sequence dataset available (*n* = 613). The corresponding phylogeographic MCC tree can be seen in Supplementary Figure S3. We observe consistent historic transmission over time from Maharashtra to most S/UT included in our model, spanning the entire latitude of India. The average of each of the five randomly down-sampled datasets showed similar routes of transmission demonstrating connections from Maharashtra to most S/UT (Supplementary Figure S4 and Table S7). Between the 14 S/UT modelled as discrete-traits, we identified 30 supported (BF > 3) routes of asymmetric transmission out of a possible 182 unique paths (Supplementary Table S8). Maharashtra was the most frequently implicated origin site for transmission (*n* = 9/30; 30%) and the most supported (221,244 > BF > 199.23) followed by Karnataka (*n* = 8/30; 27%), and Jammu and Kashmir (*n* = 4/30; 13%). Kerala was the most frequently implicated destination for transmission (*n* = 5/30; 17%), followed by Jammu and Kashmir, Punjab, and Karnataka (*n* = 3/30; 10% each). Assam, Punjab, and Rajasthan were not supported sources for transmission (BF < 1) but were variably implicated as destinations (*n* = 4/30; 13% and 1/30; 3% respectively). Maharashtra was the only state implicated as a frequent source of transmission (*n* = 6/30; 20%) but never as a destination.

### 3.4. Generalized Linear Modelling

Of the 11 demographic, ecological, and climactic factors included as predictors in the GLM, seven were supported as either promoters or protectors of A/H1N1pdm09 transmission between the 14 S/UT modelled (Table 3). The most supported factor identified in the model was great-circle-distance between any two pair-wise locations (BF = 269.3). As indicated by the negative model coefficient (β| δ = −0.53), increasing distance between states was definitively protective of viral transmission. Population density and average temperature by origin were also both protective of viral transmission between S/UT, however with reduced support (BF = 8.93 and 4.69 respectively). In contrast, sample size by origin was the most supported factor (BF = 237.2) and strongly correlated with definitive promotion of viral transmission indicated by the positive model coefficient (β| δ = 1.46). Sample size by destination was also implicated as a strong promotor of viral transmission (BF = 10.3). Other apparent promotors of A/H1N1pdm09 transmission included aviation passenger flux both by origin and destination, and sample size by destination. Passenger flux by origin had the highest median effect size coefficient (β| δ = 1.86) of all factors however the wide uncertainty of the 95% BCI means it might also be protective (−0.46 to 2.95).

## 4. Discussion

This study provides new insights into the high CFR and dynamics of A/H1N1pdm09 in India. Using the most comprehensive A/H1N1pdm09 HA sequence dataset annotated with associated temporal and spatial metadata available from India, our analysis has uncovered possible genetic explanations for the apparent high CFR of A/H1N1pdm09 observed there. Case fatality ratios aim to measure the individual risk of death among infected cases and are frequently used as a proxy for disease severity within populations [48]. In India, yearly CFR for A/H1N1pdm09 have ranged from 3.6% to 23.3%, which is orders of magnitude higher than observed in other countries (Figure 1b). The true CFR is certainly lower due to case under-ascertainment [48]. For example, in states with more than 100 reported deaths, the upper CFR range from 2009 to 2017 drops to 18.2% [19]. In order to roughly determine the degree of possible case ascertainment issues affecting CFR estimates in India, we used demographic reconstruction methods to infer the effective viral population size (N_e_) of A/H1N1pdm09 through time. In contrast to official case counts which show little activity in the three years from 2012 to 2014 (Figure 1b) we infer sizable A/H1N1pdm09 epidemics in India occurring almost yearly (Figure 1a). This suggests a high degree of case under-ascertainment during those years. Likely explanations for this include the widespread circulation of mild or subclinical strains or increasing population immunity to drifted but antigenically indistinguishable strains. In the absence of any significant changes to surveillance and reporting guidelines however, the magnitude of cases and deaths therefore detected in 2015 and 2017 suggests the emergence of antigenically novel A/H1N1pdm09 viruses. The acute magnitude and severity of the 2015 season in particular was reflected extensively in the Indian media at the time [49,50,51], and largely affected younger and middle-aged persons under 65 years and involved the deaths of healthy people [18,19,20]. Clinicians working in intensive care reported severe clinical manifestations and extensive ulceration of the trachea-bronchial tree on bronchoscopy (personal correspondence, clinician round table). This is in contrast to other countries where post-pandemic A/H1N1pdm09-predominate seasons have been relatively mild [52,53].

In our study we provide possible genetic explanations for the circulation of a severe A/H1N1pdm09 virus in India which has yet to be articulated in the literature. We provide evidence of increased positive selection within the antigenic sites of HA (Figure 2) among Indian A/H1N1pdm09 strains relative to globally circulating strains that could explain the severity of recent epidemics observed there (Table 2). Progressive selection at HA antigenic sites (i.e., antigenic drift) is principally responsible for the seasonal emergence of influenza A epidemics worldwide [54,55], and higher rates of selection at antigenic sites are known to drive the increased frequency and severity of A/H3N2 epidemics [56]. Residue changes within the four antigenic sites of A/H1N1pdm09 HA (Sa, Sb, Ca and Cb) continue to be detected worldwide [57] including S185T [58,59,60] detected in our study (Table 2; Sb domain). Residue change S185T was observed in the majority of our extant Indian sample (*n* = 473/643; 77.2%). Increased selection at position 186 however has not previously been reported. Residue changes at this position have been observed overseas among cases with severe disease concurrent with other residue changes also detected in our study (Table 2) such as S185T and D222N/G [61,62,63,64]. Outside of typical determinates associated with excess influenza mortality such as reduced access to health care in India [6,8,17], selection at these sites together provide possible genetic explanations for the unusual severity of recent epidemics in India. At site 186 particularly, the equivalent position in A/H3N2 viruses (position 189) is one of seven key positions within HA capable of generating antigenically novel variants from a single residue change [65]. Therefore, residue changes at this site, such as A186T, may constitute a new antigenic determinate of A/H1N1pdm09 severity. This could have substantial public health implications for future epidemic prevention, control, and response within India, but also for risk prediction around the world. However, the low frequency of residue changes at position 186 in our sample (*n* = 8/613; 1.31%) mean these mutations might also be sporadic. Yet, the robust evidence for increased selection relative to internationally circulating A/H1N1pdm09 viruses (Table 2) suggests an unusual situation could be occurring in India which warrants further investigation. Future studies such as haemagglutinin inhibition (HI) assays will be necessary to provide empirical evidence of the potential antigenic implications of residue changes at position 186 in A/H1N1pdm09 rather than position 189 in A/H3N2.

Our analysis also detected significantly increased selection at positions 84 and 256, relative to overseas viruses (Table 2). Along with position 163 which we found to be under increased selection in India (d_N_/d_S_ = 3.83; BCI = 2.68–5.35) but not significantly more than overseas (Table 2: BCI = 1.79–6.38), residue changes at these positions such as S84N, K163Q, and A256T (along with S185T mentioned previously) were frequently observed in our sample and are known to be characteristic of clade 6B and 6B.1 (S84N) A/H1N1pdm09 strains [66]. Clade 6B viruses are further defined by residue changes D97N and K283E in HA1, yet only position 97 and not 283 observed increased selection (d_N_/d_S_ = 3.14; BCI = 2.06–4.33) relative to overseas H1N1pdm09 viruses (Supplementary Table S5). Overall 36.1% (*n* = 221/613) of our sample comprised clade 6B H1N1pdm09 viruses, the majority (63.8%; *n* = 141/221) of which were collected in 2015 (Supplementary Table S9). Clade 6B strains have been detected globally since 2012 [66] and our results show sporadic detection of 6B resides since then (Supplementary Table S9) contrary to previous analyses [67,68] which suggested emergence in India in 2015. Notably, a previous study from 2014 found an association between clade 6B viruses with reduced immune responses among younger and middle-aged persons born after 1985 [69]. As the majority of sequences isolated during the severe 2015 season (96.1%; *n* = 141/147) comprised of 6B viruses, the increased selection at these sites relative to overseas viruses (excluding K163Q and K283E) provides additional explanation for increased morbidity and mortality observed in India during that season. By 2017, 77.7% (*n* = 7/9) of the sequenced dataset was of clade 6B.1, although there was little data available from that year. Following the widespread global circulation of clade 6B and subclade 6B.1 (S84N, S162N, and I216T) residues, the A/H1N1pdm09 vaccine strain was updated for the first time since 2009 (from A/California/7/2009 to A/Michigan/45/2015) during the 2017–2019 Southern Hemisphere influenza seasons [70,71]. The 2019–2020 Northern Hemisphere recommendation includes another updated A/H1N1pdm09 vaccine strain, A/Brisbane/02/2018 from clade 6B.1A, which includes additional residue changes [72]. Since the uptake of the influenza vaccine however remains low in India, the increased selection and significant emergence of clade 6B in 2015 and 6B.1 in 2017 could have contributed to the high morbidity and mortality observed there. Increasing influenza vaccination rates should help to reduce the impact of future A/H1N1pdm09 epidemics in India.

Finally, we calculated selection ratios at position 222 up to eight times greater than the equivalent sample of globally circulating A/H1N1pdm09 (Table 2), the highest of all sites detected. Variants at this position, particularly D222G/N are known to preferentially bind to α2,3-linked sialic acid (α2,3-SA) receptors necessary for lower respiratory tract colonisation in humans [73,74] and have demonstrated an associated 11% increase in morbidity and 23% increase in mortality in cases infected with D222G/N mutants [75,76]. Earlier studies had shown A/H1N1pdm09 strains circulating in India in 2014 had acquired residue changes D222N in HA [77,78] although this was contested by the National Institute of Virology in Pune [79]. In our sample of 613 Indian taxa we identified both G222 and N222 residues, albeit at low frequencies, 3.1% (*n* = 19/613) and 1.6% (*n* = 10/613) respectively, and only once in 2015. Like position 186, the sporadic detection of residue changes at position 222 means the impact on the observed morbidity and morbidity in India is not clear. However, the disproportionate increase in selection at position 222 (Table 2) further exemplifies the unusual epidemiological situation in the country. Because short-term selection is a stochastic process [80] but long-term is known to involve complex interactions between A/H1N1pdm09 evolution, host response, and human behavior [54], the observed increase in pervasive positive selection in India could therefore be the result of between-host transmission characteristics unique to the country and warrants further investigation [54].

Overall the combination of increased selection for and circulation of clade 6B genogroups, increased selection of known determinates of A/H1N1pdm09 severity such as D222N/G, and the identification of potentially new antigenic determinates of severity such as A186T, together provides credible genomic evidence that might explain the increased frequency and severity of A/H1N1pdm09 epidemics in India. Seasonal surveillance of clade 6B residues (and related subclades), D222G/N, and residue changes at site 186 may assist with the early detection of severe epidemics in the future.

Our study is also the first to simultaneously integrate GLM and Bayesian phylogeography analysis methods to empirically quantify the transmission dynamics of A/H1N1pdm09 in India revealing supported routes of A/H1N1pdm09 transmission. We have identified Maharashtra state as a key location disseminating A/H1N1pdm09 to many S/UT including Rajasthan, Tamil Nadu, Delhi, Jammu and Kashmir, Karnataka, Madhya Pradesh, West Bengal, Assam, and Kerala (Figure 3). These results could have important implications for surveillance, risk assessment, and epidemic control strategies including vaccination in the country, as they could also be generalisable for other influenza A viruses such as A/H3N2 or other potentially pandemic variants that could emerge in the future. For example, India, along with East and Southeast Asia has been shown to be a significant source for globally circulating influenza A/H3N2 viruses [11]. The emergence of a novel pandemic influenza A strain in India represents a significant health risk to the global population. Therefore understanding the transmission patterns of influenza A within India allows for rapid risk assessment not only within the country, but also risk assessment for subsequent spread around the world.

The identification of key climactic, demographic, and ecological factors associated with A/H1N1pdm09 transmission within India similarly indicates opportunities for targeted interventions during outbreaks that could also be generalisable among S/UT not included in our model. For example, increasing distance between S/UT was the most significant (BF = 269.3) factor identified as contributing to the model and was associated with the prevention of transmission (Table 3). Other preventative factors while immutable included increasing population density and average temperature by originating S/UT. This suggests that S/UT with lower average temperatures are more likely to transmit A/H1N1pdm09 to other S/UT in India. This appears consistent with previous biological evidence suggesting cooler temperatures are favourable for influenza transmission [81,82] and the known seasonality of influenza in India where large temperature variations are observed between Northern India and Southern India during winter [83,84]. High passenger flux by both origin and destination was another factor shown to be on average predictive of transmission between S/UT in India, but the evidence was inconclusive (Table 3). Taken together, public health decision makers might choose to prioritise efforts restricting human movement between neighbouring S/UT, and potentially restricting key domestic flights with high passenger flux.

Overall this study has some key limitations. First, sample representativeness is a limitation of any epidemiological study, including phylogeography. Only 14 S/UT were included in our model due to limited sample availability or unrepresentative sampling (five states with less than two sequences per 10 million population were removed). This includes less than half of the 36 S/UT in India. Ancestral state reconstruction methods like those used by discrete-trait phylogeography can only generate inferences from traits assigned to the extant taxa. For example, no sequences were available from Gujarat, which has been affected by particularly large outbreaks (approximately 7100 cases in 2015 and 7700 in 2017), meaning inferred transmission to and from Gujarat while perhaps expected, remains unresolved in our model. Limited sample availability may also have the effect of exaggerating the evidence of transmission between any two pair-wise locations in the model, for example, where Gujarat or another unsampled state may have acted as intermediate locations for transmission. Sample size by origin was also implicated as a predictor of transmission in the complete Indian dataset (*n* = 613), which suggests sampling bias may be affecting our primary results. For example, Maharashtra was the most highly supported location for transmission to other S/UT, but also had the most sequences available (*n* = 242/613; 39.5%). Discrete-trait Bayesian phylogeography is known to be susceptible to sampling bias and, in such cases, produces overestimates of origin support [85,86]. It is reassuring however that the results from each of the five randomly down-sampled datasets showed not dissimilar evidence for transmission from Maharashtra and other S/UT (Supplementary Figure S4, Table S7). Furthermore, Maharashtra also endured the largest recorded outbreaks (approximately 8500 cases in 2015 and 6100 cases in 2017 [16]) and had the highest average number of domestic passengers between 2009 and 2017 [40] suggesting our primary results may in fact reflect the truth. However, measures of transit between states in our GLM model only extended to domestic aviation. Other significant means of interstate travel in India such as rail (nationally 8.1 billion passengers in 2017 [87]) was not considered in our model due to the lack of S/UT transit data. We hypothesize that rail-travel would have a significant effect on A/H1N1pdm09 transmission in India, particularly over short distances, however the absence of this data does not affect the interpretation of our results. Rather, any effect of rail-travel remains unresolved. Future studies could investigate the effect of rail-travel if state-based data were to become available.

Lastly, only HA sequences were included in our analysis due to the limited availability of whole A/H1N1pdm09 genomes from India. Residue variations among other genes such as Polymerase basic protein 2 (PB2) have been shown to affect viral replication in other influenza A viruses such as A/H5N1 which are associated with increased morbidity and mortality [88]. We cannot know if other more important residue changes may be driving the frequency and severity of A/H1N1pdm09 outbreaks in India, rather than selection within HA. Future studies however could investigate selection pressures and residue variations within PB2, among the other gene segments of influenza A.

## 5. Conclusions

Our findings have important implications in understanding the dynamics of influenza A/H1N1pdm09 transmission and evolution in India which could inform future public health prevention and control efforts in the country. We have identified increased selection pressure at multiple meaningful HA residue positions including site 222 and clade 6B characteristic residues relative to internationally circulating viruses that may explain the high CFR observed there, particularly the abnormally severe 2015 and 2017 seasons, and signifies a link to the unique public health burden of the virus in India. We believe this is the first study to observe increased selection at site 186 and believe this site could be a potential determinate of A/H1N1pdm09 severity. Future investigations however are warranted to confirm the antigenic potential of residue changes at this position and associated impact on morbidity and mortality. We have revealed national routes of A/H1N1pdm09 transmission in India identifying Maharashtra as the most supported state for spread throughout the country while quantifying climactic, ecological, and transport factors as drivers of within-country transmission. Together these results have important implications for future A/H1N1pdm09 surveillance and control within India but also for epidemic and pandemic risk prediction around the world. Strengthening influenza surveillance capacity in the country should remain a priority, first to improve estimates of severity, and second, to prepare for future epidemics and pandemics. Continuous monitoring of haemagglutinin changes remains vital for public health surveillance in India.

## Figures and Tables

**Table 1:** Haemagglutinin (HA) sequence dataset by year and State and Union Territories (S/UT) of India included for analysis.

	Year										
S/UT (Population 10^6^)	2009	2010	2011	2012	2013	2014	2015	2016	2017	Total	%
Assam (31.2)	4	4					1	11		20	3.3
Delhi (16.8)	10	4	7	14	6	2	20			63	10.3
Goa (1.5)	1	3	3	3						10	1.6
Haryana (25.4)	1	2	1	3	3		2			12	2.0
Jammu and Kashmir (12.5)	1	2	8	29	7		11			58	9.5
Karnataka (61.1)	19	16	5	15					6	61	10.0
Kerala (33.4)	1	2		9	2	3	3		2	22	3.6
Madhya Pradesh (72.6)	4	8	4	5	5		18			44	7.2
Maharashtra (112.4)	42	8	5	54	66	6	53	8		242	39.5
Punjab (27.7)	1	2	4							7	1.1
Rajasthan (68.6)	3	1		3	5		6			18	2.9
Tamil Nadu (72.15)	5			2		2	6		1	16	2.6
Uttarakhand (10.1)	1	1		1	2		1			6	1.0
West Bengal (91.3)	3			5			26			34	5.5
Year Total	96	53	37	143	96	13	147	19	9	613	100.0

**Table 2:** d_N_/d_S_ ratios of codon sites identified in India under pervasive positive selection relative to d_N_/d_S_ ratios of two distinct international samples S1 and S2.

Site (H3 ^a^)	Ag ^b^	India Taxa (*n* = 613)		International (S1)		International (S2)	
		d_N_/d_S_	95% BCI	d_N_/d_S_	95% BCI	d_N_/d_S_	95% BCI
84 (92)	*n*/a	8.14	(5.68–10.92)	2.53	(1.69–3.45)	3.51	(2.33–4.79)
163 (166)	Sa	3.83	(2.68–5.35)	4.54	(2.98–6.38)	2.62	(1.79–3.68)
185 (188)	Sb	4.50	(3.18–6.21)	2.14	(1.41–2.94)	1.13	(0.76–1.54)
186 (189)	Sb	3.35	(2.26–4.51)	1.44	(0.97–1.94)	1.47	(0.93–1.97)
222 (225)	Ca	13.42	(9.43–18.42)	3.42	(2.30–4.81)	4.32	(2.87–5.85)
256 (259)	*n*/a	4.39	(3.12–6.09)	1.16	(0.74–1.58)	1.08	(0.75–1.48)

^a^ Relative H3 numbering determined by FluDB HA Subtype Numbering algorithm [45]. ^b^ Antigenic Domain (Ag).

**Table 3:** Predictors of A/H1N1pdm09 transmission in India between 14 S/UT from 2009 to 2017.

Predictor	E(δ) Probability	(β| δ) Coefficient	95% BCI	BF ^a^
Distance	0.88	−0.53	−0.79 to −0.28	269.3
SS origin	0.87	1.46	0.96 to 2.12	237.2
P Flux destination	0.31	0.35	0.15 to 0.56	15.67
SS destination	0.22	0.26	0.11 to 0.42	10.03
Pop Dense origin	0.2	−1.43	−2.82 to −0.28	8.93
P Flux origin	0.14	1.86	−0.46 to 2.95	5.84
Average temp origin	0.12	−0.49	−0.8 to −0.18	4.69

^a^ Predictors are ordered by decreasing significance (BF) and probability of inclusion (E(δ)) in the model as a measure of the likelihood of impact on transmission.

## Supplementary Materials

The following are available online at https://www.mdpi.com/1999-4915/11/9/791/s1: Figure S1: Spatial distribution of 613 taxa included in the final dataset sampled in India between 2009 and 2017 by S/UT. Figure S2: Root-to-tip regression over time of 613 H1N1pdm09 isolates sampled from India between 2009 and 2017 inclusive. Figure S3: Maximum clade credibility tree (MCC) of 613 A/H1N1pdm09 taxa sequenced for HA between 2009 and 2017 inclusive from 14 states and union territories (S/UT) in India. Figure S4: Definitively supported (BF > 100) routes of A/H1N1pdm09 transmission between S/UT in India from 2009 to 2017 based on five randomly subsampled datasets. Table S1: Global dataset of HA sequences by year and region for comparative analysis. Table S2: Substitution model and clock prior testing results using path sampling (PS) and stepping-stone sampling (SSS) of log marginal likelihoods. Table S3: Interpretation of computed BF values. Table S4: Subsample of HA sequences by year and S/UT included for replicate analysis. Table S5: dN/dS rate ratios and 95% BCI of all positively selected HA sites among Indian (n = 613) and International (S1 and S2) taxa of H1N1pdm09 detected using BRC. Table S6: Codon sites under pervasive positive selection as identified using Bayesian Renaissance Counting (BRC), two-rate fixed effects likelihood (FEL) and single-likelihood ancestor counting (SLAC) in India. Table S7: Statistically supported routes for A/H1N1pdm09 transmission in India between 14 S/UT from 2009 to 2017 based on the average of five randomly subsampled sequence datasets (n = 259). Table S8: Statistically supported routes for A/H1N1pdm09 transmission in India between 14 S/UT discrete locations from 2009 to 2017. Table S9: Distribution of A/H1N1pdm09 viruses belonging to clade 6B by year and S/UT in India between 2009 and 2011. Acknowledgment Table 1: GISAID EpiFlu™ Database Acknowledgment Table. Datafile 1: Alignment of 613 A/H1N1pdm09 HA isolates.
