# Supplementary material for: Phylodynamics of Influenza A/H1N1pdm09 in India Reveals Circulation Patterns and Increased Selection for Clade 6b Residues and Other High Mortality Mutants
Source: Viruses. 2019 Aug 27;11(9):791. doi: 10.3390/v11090791 (PMC6783925; doi:10.3390/v11090791)
Supplement: Supplementary file 1 [file viruses-11-00791-s001.zip › Acknowledgment Table 1.docx]

**GISAID EpiFlu™ Database Acknowledgment Table**

We gratefully acknowledge the authors and originating and submitting laboratories of the sequences from GISAID’s EpiFlu™ Database on which this research is based. The list is detailed below.

| **Isolate ID** | **Patient Location** | **Collection Date** | **Isolate Name** | **Submitting Laboratory** | **Authors/Isolate Submitter** |
| --- | --- | --- | --- | --- | --- |
| EPI_ISL_279404 | Rajasthan | 2009 | A/Jodhpur/1316/2009 | Other Database Import | Angel,B.; Angel,A.; Joshi,A.P.; Baharia,R.K.; Rathore,S.; Joshi,V. |
| EPI_ISL_71311 | Karnataka | 2009-06 | A/Blore/NIV236/2009 | Other Database Import | Mishra,A.; Potdar,V.; Chadha,M.; Jadhav,S.; Mullick,J.; Cherian,S. |
| EPI_ISL_75212 | Maharashtra | 2009-06 | A/Mum/NIV261/2009 | Other Database Import | Mullick,J.; Potdar,V.A.; Payyapilly,B.J.; Keng,S.S.; Cherian,S.S.; Jadhav,S.M.; Mishra,A.C. |
| EPI_ISL_75213 | Delhi | 2009-06 | A/Delhi/NIV57/2009 | Other Database Import | Mullick,J.; Potdar,V.A.; Payyapilly,B.J.; Keng,S.S.; Cherian,S.S.; Jadhav,S.M.; Mishra,A.C. |
| EPI_ISL_70312 | Maharashtra | 2009-06-10 | A/India/4725/2009 | Centers for Disease Control and Prevention | Garten,R. |
| EPI_ISL_95145 | Maharashtra | 2009-06-21 | A/Pune/NIV161/2009 | Other Database Import | Mullick,J.; Potdar,V.A. |
| EPI_ISL_95133 | Tamil Nadu | 2009-06-27 | A/Assam/RMRC_709/2016 | Other Database Import | Borkakoty,B.; Jakharia,A.; Sarmah,K.; Hazarika,R.; Biswas,D.; Mahanta,J. |
| EPI_ISL_95132 | Tamil Nadu | 2009-06-27 | A/Kolar/MCVRAF3154/2017 | Other Database Import | Jagadesh,A.; Arunkumar,G. |
| EPI_ISL_71312 | Karnataka | 2009-07 | A/Blore/NIV310/2009 | Other Database Import | Mishra,A.; Potdar,V.; Chadha,M.; Jadhav,S.; Mullick,J.; Cherian,S. |
| EPI_ISL_75215 | Maharashtra | 2009-07 | A/Pune/NIV807/2009 | Other Database Import | Mullick,J.; Potdar,V.A.; Chadha,M.; Payyapilly,B.J.; Keng,S.S.; Cherian,S.S.; Jadhav,S.M.; Mishra,A.C. |
| EPI_ISL_75220 | Maharashtra | 2009-07 | A/Mum/NIV968/2009 | Other Database Import | Mullick,J.; Potdar,V.A.; Chadha,M.; Payyapilly,B.J.; Keng,S.S.; Cherian,S.S.; Jadhav,S.M.; Mishra,A.C. |
| EPI_ISL_75214 | Tamil Nadu | 2009-07 | A/Assam/RMRC_711/2016 | Other Database Import | Borkakoty,B.; Jakharia,A.; Sarmah,K.; Hazarika,R.; Biswas,D.; Mahanta,J. |
| EPI_ISL_95126 | Maharashtra | 2009-07-07 | A/Mum/NIV398/2009 | Other Database Import | Mullick,J.; Potdar,V.A. |
| EPI_ISL_95125 | Tamil Nadu | 2009-07-15 | A/Assam/RMRC_693/2016 | Other Database Import | Borkakoty,B.; Jakharia,A.; Sarmah,K.; Hazarika,R.; Biswas,D.; Mahanta,J. |
| EPI_ISL_95124 | Maharashtra | 2009-07-21 | A/Pune/NIV652/2009 | Other Database Import | Mullick,J.; Cherian,S.S.; Potdar,V.A.; Chadha,M.S.; Mishra,A.C. |
| EPI_ISL_95123 | Maharashtra | 2009-07-25 | A/Pune/NIV759/2009 | Other Database Import | Mullick,J.; Potdar,V.A. |
| EPI_ISL_161527 | Delhi | 2009-07-29 | A/Delhi/018/2009 | Other Database Import | Kumar,S.; Khare,S.; Saidullah,B.; Gandhok,I.; Rai,A. |
| EPI_ISL_71313 | Delhi | 2009-08 | A/Delhi/NIV3610/2009 | Other Database Import | Mishra,A.; Potdar,V.; Chadha,M.; Jadhav,S.; Mullick,J.; Cherian,S.; Mullick,J.; Cherian,S.S.; Potdar,V.A.; Chadha,M.S.; Mishra,A.C. |
| EPI_ISL_71316 | Maharashtra | 2009-08 | A/Mum/NIV5442/2009 | Other Database Import | Mishra,A.; Potdar,V.; Chadha,M.; Jadhav,S.; Mullick,J.; Cherian,S. |
| EPI_ISL_71320 | Maharashtra | 2009-08 | A/Pune/NIV6196/2009 | Other Database Import | Mishra,A.; Potdar,V.; Chadha,M.; Jadhav,S.; Mullick,J.; Cherian,S. |
| EPI_ISL_71321 | Maharashtra | 2009-08 | A/Pune/NIV6447/2009 | Other Database Import | Mishra,A.; Potdar,V.; Chadha,M.; Jadhav,S.; Mullick,J.; Cherian,S. |
| EPI_ISL_71322 | Maharashtra | 2009-08 | A/Pune/NIV8489/2009 | Other Database Import | Mishra,A.; Potdar,V.; Chadha,M.; Jadhav,S.; Mullick,J.; Cherian,S. |
| EPI_ISL_71323 | Maharashtra | 2009-08 | A/Pune/NIV9355/2009 | Other Database Import | Mishra,A.; Potdar,V.; Chadha,M.; Jadhav,S.; Mullick,J.; Cherian,S. |
| EPI_ISL_75208 | Karnataka | 2009-08 | A/Blore/NIV1189/2009 | Other Database Import | Mullick,J.; Potdar,V.A.; Chadha,M.; Payyapilly,B.J.; Keng,S.S.; Cherian,S.S.; Jadhav,S.M.; Mishra,A.C. |
| EPI_ISL_75216 | Maharashtra | 2009-08 | A/Mum/NIV9312/2009 | Other Database Import | Mullick,J.; Potdar,V.A.; Chadha,M.; Payyapilly,B.J.; Keng,S.S.; Cherian,S.S.; Jadhav,S.M.; Mishra,A.C. |
| EPI_ISL_75217 | Maharashtra | 2009-08 | A/Dhule/NIV9433/2009 | Other Database Import | Mullick,J.; Potdar,V.A.; Chadha,M.; Payyapilly,B.J.; Keng,S.S.; Cherian,S.S.; Jadhav,S.M.; Mishra,A.C. |
| EPI_ISL_75218 | Maharashtra | 2009-08 | A/Jalna/NIV9436/2009 | Other Database Import | Mullick,J.; Potdar,V.A.; Chadha,M.; Payyapilly,B.J.; Keng,S.S.; Cherian,S.S.; Jadhav,S.M.; Mishra,A.C. |
| EPI_ISL_75219 | Maharashtra | 2009-08 | A/Ytml/NIV9438/2009 | Other Database Import | Mullick,J.; Potdar,V.A.; Chadha,M.; Payyapilly,B.J.; Keng,S.S.; Cherian,S.S.; Jadhav,S.M.; Mishra,A.C. |
| EPI_ISL_95152 | Maharashtra | 2009-08-05 | A/Mum/NIV1134/2009 | Other Database Import | Mullick,J.; Potdar,V.A. |
| EPI_ISL_95153 | Maharashtra | 2009-08-05 | A/Mum/NIV1126/2009 | Other Database Import | Mullick,J.; Potdar,V.A. |
| EPI_ISL_161509 | Karnataka | 2009-08-06 | A/Karnataka/001/2009 | Other Database Import | Kumar,S.; Khare,S.; Saidullah,B.; Gandhok,I.; Rai,A. |
| EPI_ISL_95150 | Maharashtra | 2009-08-06 | A/Pune/NIV1166/2009 | Other Database Import | Mullick,J.; Potdar,V.A. |
| EPI_ISL_161510 | Kerala | 2009-08-14 | A/Kerala/002/2009 | Other Database Import | Kumar,S.; Khare,S.; Saidullah,B.; Gandhok,I.; Rai,A. |
| EPI_ISL_190141 | Karnataka | 2009-08-19 | A/Bangalore/2960-03/2009 | Other Database Import | Thomas,M.; Desai,A.; Vasanthapuram,R. |
| EPI_ISL_71314 | Delhi | 2009-09 | A/Delhi/NIV3704/2009 | Other Database Import | Mishra,A.; Potdar,V.; Chadha,M.; Jadhav,S.; Mullick,J.; Cherian,S. |
| EPI_ISL_71317 | Maharashtra | 2009-09 | A/Mum/NIV9945/2009 | Other Database Import | Mishra,A.; Potdar,V.; Chadha,M.; Jadhav,S.; Mullick,J.; Cherian,S. |
| EPI_ISL_71318 | Maharashtra | 2009-09 | A/Pune/NIV10278/2009 | Other Database Import | Mishra,A.; Potdar,V.; Chadha,M.; Jadhav,S.; Mullick,J.; Cherian,S. |
| EPI_ISL_71319 | Maharashtra | 2009-09 | A/Pune/NIV10604/2009 | Other Database Import | Mishra,A.; Potdar,V.; Chadha,M.; Jadhav,S.; Mullick,J.; Cherian,S. |
| EPI_ISL_95154 | Maharashtra | 2009-09-05 | A/Ngp/NIV11203/2009 | Other Database Import | Mullick,J.; Potdar,V.A. |
| EPI_ISL_190142 | Karnataka | 2009-09-08 | A/Bangalore/6053-14/2009 | Other Database Import | Thomas,M.; Desai,A.; Vasanthapuram,R. |
| EPI_ISL_190143 | Karnataka | 2009-09-09 | A/Bangalore/6206-12/2009 | Other Database Import | Thomas,M.; Desai,A.; Vasanthapuram,R. |
| EPI_ISL_190144 | Karnataka | 2009-09-16 | A/Bangalore/6974-08/2009 | Other Database Import | Thomas,M.; Desai,A.; Vasanthapuram,R. |
| EPI_ISL_95156 | West Bengal | 2009-09-20 | A/Kol/NIV105774/2009 | Other Database Import | Mullick,J.; Potdar,V.A. |
| EPI_ISL_95161 | Delhi | 2009-09-30 | A/Delhi/NIV0951831/2009 | Other Database Import | Mullick,J.; Potdar,V.A. |
| EPI_ISL_95162 | Delhi | 2009-09-30 | A/Delhi/NIV0951830/2009 | Other Database Import | Mullick,J.; Potdar,V.A. |
| EPI_ISL_161514 | Goa | 2009-10-06 | A/Goa/006/2009 | Other Database Import | Kumar,S.; Khare,S.; Saidullah,B.; Gandhok,I.; Rai,A. |
| EPI_ISL_95148 | Maharashtra | 2009-10-06 | A/Ngp/NIV14725/2009 | Other Database Import | Mullick,J.; Potdar,V.A. |
| EPI_ISL_95159 | Delhi | 2009-10-06 | A/Delhi/NIV0951833/2009 | Other Database Import | Mullick,J.; Potdar,V.A. |
| EPI_ISL_95160 | Delhi | 2009-10-06 | A/Delhi/NIV0951832/2009 | Other Database Import | Mullick,J.; Potdar,V.A. |
| EPI_ISL_190145 | Karnataka | 2009-10-07 | A/Bangalore/8330-09/2009 | Other Database Import | Thomas,M.; Desai,A.; Vasanthapuram,R. |
| EPI_ISL_95147 | Maharashtra | 2009-10-07 | A/Pune/NIV14886/2009 | Other Database Import | Mullick,J.; Potdar,V.A. |
| EPI_ISL_161520 | Madhya Pradesh | 2009-10-10 | A/Madhya Pradesh/011/2009 | Other Database Import | Kumar,S.; Khare,S.; Saidullah,B.; Gandhok,I.; Rai,A. |
| EPI_ISL_95146 | Maharashtra | 2009-10-11 | A/Pune/NIV15492/2009 | Other Database Import | Mullick,J.; Potdar,V.A. |
| EPI_ISL_96755 | Assam | 2009-10-14 | A/Assam/2220/2009 | Other Database Import | Biswas,D.; Lahan,M.; Yadav,K.; Borkakoty,B.; Mahanta,J. |
| EPI_ISL_95155 | West Bengal | 2009-10-15 | A/Kol/NIV105777/2009 | Other Database Import | Mullick,J.; Potdar,V.A. |
| EPI_ISL_190146 | Karnataka | 2009-10-20 | A/Bangalore/8973-04/2009 | Other Database Import | Thomas,M.; Desai,A.; Vasanthapuram,R. |
| EPI_ISL_190147 | Karnataka | 2009-10-20 | A/Bangalore/8975-19/2009 | Other Database Import | Thomas,M.; Desai,A.; Vasanthapuram,R. |
| EPI_ISL_66666 | Maharashtra | 2009-10-20 | A/India/8489/2009 | Centers for Disease Control and Prevention | Garten,R. |
| EPI_ISL_190148 | Karnataka | 2009-10-26 | A/Bangalore/9274-06/2009 | Other Database Import | Thomas,M.; Desai,A.; Vasanthapuram,R. |
| EPI_ISL_206026 | Assam | 2009-10-28 | A/Assam/2257/2009 | Other Database Import | Biswas,D.; Sarmah,K.; Dutta,M.; Buragohain,M.; Yadav,K.; Borkakoty,B. |
| EPI_ISL_206025 | Assam | 2009-10-29 | A/Assam/2264/2009 | Other Database Import | Biswas,D.; Sarmah,K.; Dutta,M.; Buragohain,M.; Yadav,K.; Borkakoty,B. |
| EPI_ISL_206027 | Assam | 2009-11-06 | A/Assam/2283/2009 | Other Database Import | Biswas,D.; Sarmah,K.; Dutta,M.; Buragohain,M.; Yadav,K.; Borkakoty,B. |
| EPI_ISL_161521 | Tamil Nadu | 2009-11-06 | A/Assam/RMRC_609/2016 | Other Database Import | Borkakoty,B.; Jakharia,A.; Sarmah,K.; Hazarika,R.; Biswas,D.; Mahanta,J. |
| EPI_ISL_161515 | Punjab | 2009-11-26 | A/Punjab/007/2009 | Other Database Import | Kumar,S.; Khare,S.; Saidullah,B.; Gandhok,I.; Rai,A. |
| EPI_ISL_161517 | Rajasthan | 2009-11-26 | A/Rajasthan/009/2009 | Other Database Import | Kumar,S.; Khare,S.; Saidullah,B.; Gandhok,I.; Rai,A. |
| EPI_ISL_190149 | Karnataka | 2009-11-28 | A/Bangalore/10519-13/2009 | Other Database Import | Thomas,M.; Desai,A.; Vasanthapuram,R. |
| EPI_ISL_201011 | Madhya Pradesh | 2009-11-30 | A/Indore/10/2009 | Other Database Import | Sahu,M.; Shukla,M.K.; Singh,N.; Barde,P.V. |
| EPI_ISL_75209 | Maharashtra | 2009-12 | A/Pune/NIV20007/2009 | Other Database Import | Mullick,J.; Potdar,V.A.; Chadha,M.; Payyapilly,B.J.; Keng,S.S.; Cherian,S.S.; Jadhav,S.M.; Mishra,A.C. |
| EPI_ISL_75210 | Maharashtra | 2009-12 | A/Pune/NIV21115/2009 | Other Database Import | Mullick,J.; Potdar,V.A.; Chadha,M.; Payyapilly,B.J.; Keng,S.S.; Cherian,S.S.; Jadhav,S.M.; Mishra,A.C. |
| EPI_ISL_75211 | Maharashtra | 2009-12 | A/Pune/NIV21123/2009 | Other Database Import | Mullick,J.; Potdar,V.A.; Chadha,M.; Payyapilly,B.J.; Keng,S.S.; Cherian,S.S.; Jadhav,S.M.; Mishra,A.C. |
| EPI_ISL_190150 | Karnataka | 2009-12-03 | A/Bangalore/10695-10/2009 | Other Database Import | Thomas,M.; Desai,A.; Vasanthapuram,R. |
| EPI_ISL_190151 | Karnataka | 2009-12-03 | A/Bangalore/10698-02/2009 | Other Database Import | Thomas,M.; Desai,A.; Vasanthapuram,R. |
| EPI_ISL_176800 | Jammu & Kashmir | 2009-12-04 | A/Jammu & Kashmir/013/2009 | Other Database Import | Kumar,S.; Khare,S.; Saidullah,B.; Rai,A. |
| EPI_ISL_279400 | Rajasthan | 2009-12-04 | A/Jodhpur/526/2009 | Other Database Import | Angel,B.; Angel,A.; Joshi,A.P.; Baharia,R.K.; Rathore,S.; Joshi,V. |
| EPI_ISL_95144 | Maharashtra | 2009-12-05 | A/Pune/NIV19609/2009 | Other Database Import | Mullick,J.; Potdar,V.A. |
| EPI_ISL_161512 | West Bengal | 2009-12-06 | A/West Bengal/004/2009 | Other Database Import | Kumar,S.; Khare,S.; Saidullah,B.; Gandhok,I.; Rai,A. |
| EPI_ISL_161523 | Uttarakhand | 2009-12-08 | A/Uttrakhand/014/2009 | Other Database Import | Kumar,S.; Khare,S.; Saidullah,B.; Gandhok,I.; Rai,A. |
| EPI_ISL_95143 | Maharashtra | 2009-12-10 | A/Pune/NIV19934/2009 | Other Database Import | Mullick,J.; Potdar,V.A. |
| EPI_ISL_95142 | Maharashtra | 2009-12-11 | A/Pune/NIV20069/2009 | Other Database Import | Mullick,J.; Potdar,V.A. |
| EPI_ISL_161526 | Delhi | 2009-12-13 | A/Delhi/017/2009 | Other Database Import | Kumar,S.; Khare,S.; Saidullah,B.; Gandhok,I.; Rai,A. |
| EPI_ISL_161525 | Delhi | 2009-12-14 | A/Delhi/016/2009 | Other Database Import | Kumar,S.; Khare,S.; Saidullah,B.; Gandhok,I.; Rai,A. |
| EPI_ISL_190152 | Karnataka | 2009-12-15 | A/Bangalore/11210-01/2009 | Other Database Import | Thomas,M.; Desai,A.; Vasanthapuram,R. |
| EPI_ISL_161524 | Haryana | 2009-12-16 | A/Haryana/015/2009 | Other Database Import | Kumar,S.; Khare,S.; Saidullah,B.; Gandhok,I.; Rai,A. |
| EPI_ISL_167443 | Madhya Pradesh | 2009-12-16 | A/Indore/59/2009 | Other Database Import | Barde,P.V.; Sahu,M.; Shukla,M.K.; Kori,B.K.; Sharma,R.K.; Singh,N. |
| EPI_ISL_190153 | Karnataka | 2009-12-19 | A/Bangalore/11406-11/2009 | Other Database Import | Thomas,M.; Desai,A.; Vasanthapuram,R. |
| EPI_ISL_95158 | Maharashtra | 2009-12-21 | A/Pune/NIV0953725/2009 | Other Database Import | Mullick,J.; Potdar,V.A. |
| EPI_ISL_95140 | Maharashtra | 2009-12-23 | A/Pune/NIV21139/2009 | Other Database Import | Mullick,J.; Potdar,V.A. |
| EPI_ISL_95141 | Maharashtra | 2009-12-23 | A/Pune/NIV21090/2009 | Other Database Import | Mullick,J.; Potdar,V.A. |
| EPI_ISL_95135 | Maharashtra | 2009-12-24 | A/Pune/NIV21259/2009 | Other Database Import | Mullick,J.; Potdar,V.A. |
| EPI_ISL_95136 | Maharashtra | 2009-12-24 | A/Pune/NIV21218/2009 | Other Database Import | Mullick,J.; Potdar,V.A. |
| EPI_ISL_95138 | Maharashtra | 2009-12-24 | A/Pune/NIV21163/2009 | Other Database Import | Mullick,J.; Potdar,V.A. |
| EPI_ISL_95139 | Maharashtra | 2009-12-24 | A/Pune/NIV21159/2009 | Other Database Import | Mullick,J.; Potdar,V.A. |
| EPI_ISL_95137 | Maharashtra | 2009-12-25 | A/Pune/NIV21193/2009 | Other Database Import | Mullick,J.; Potdar,V.A. |
| EPI_ISL_151997 | Madhya Pradesh | 2009-12-26 | A/Jabalpur/112/2009 | Other Database Import | Barde,P.V.; Sahu,M.; Shukla,M.K.; Kori,B.K.; Sahare,L. |
| EPI_ISL_190154 | Karnataka | 2009-12-26 | A/Bangalore/11658-15/2009 | Other Database Import | Thomas,M.; Desai,A.; Vasanthapuram,R. |
| EPI_ISL_190155 | Karnataka | 2009-12-31 | A/Bangalore/12220-18/2009 | Other Database Import | Thomas,M.; Desai,A.; Vasanthapuram,R. |
| EPI_ISL_161528 | Rajasthan | 2010-01-10 | A/Rajasthan/019/2010 | Other Database Import | Kumar,S.; Khare,S.; Saidullah,B.; Gandhok,I.; Rai,A. |
| EPI_ISL_95134 | Maharashtra | 2010-01-13 | A/Ngp/NIV22704/2010 | Other Database Import | Mullick,J.; Potdar,V.A. |
| EPI_ISL_176802 | Jammu & Kashmir | 2010-01-15 | A/Jammu & Kashmir/022/2010 | Other Database Import | Kumar,S.; Khare,S.; Saidullah,B.; Rai,A. |
| EPI_ISL_167444 | Madhya Pradesh | 2010-01-21 | A/Khargone/293/2010 | Other Database Import | Barde,P.V.; Sahu,M.; Shukla,M.K.; Kori,B.K.; Sharma,R.K.; Singh,N. |
| EPI_ISL_161529 | Assam | 2010-01-28 | A/Assam/020/2010 | Other Database Import | Kumar,S.; Khare,S.; Saidullah,B.; Gandhok,I.; Rai,A. |
| EPI_ISL_161533 | Madhya Pradesh | 2010-01-28 | A/Madhya Pradesh/024/2010 | Other Database Import | Kumar,S.; Khare,S.; Saidullah,B.; Gandhok,I.; Rai,A. |
| EPI_ISL_201012 | Madhya Pradesh | 2010-02-09 | A/Indore/379/2010 | Other Database Import | Sahu,M.; Shukla,M.K.; Singh,N.; Barde,P.V. |
| EPI_ISL_95128 | Maharashtra | 2010-03-09 | A/Pune/NIV26410/2010 | Other Database Import | Mullick,J.; Potdar,V.A. |
| EPI_ISL_104331 | Karnataka | 2010-03-22 | A/India/Blore/2010 | Other Database Import | Sharma,S.; Parida,M.M.; Shukla,J.; Joshi,G.; Dash,P.; Rao,P.V.L. |
| EPI_ISL_77718 | Maharashtra | 2010-04-03 | A/India/3725/2010 | Centers for Disease Control and Prevention | Garten,R. |
| EPI_ISL_95157 | Maharashtra | 2010-04-03 | A/Vadu/NIV1043725/2010 | Other Database Import | Mullick,J.; Potdar,V.A. |
| EPI_ISL_79358 | Maharashtra | 2010-05-08 | A/India/8910/2010 | Centers for Disease Control and Prevention | Garten,R. |
| EPI_ISL_79711 | Maharashtra | 2010-05-08 | A/India/8910/2010 | Centers for Disease Control and Prevention | Garten,R. |
| EPI_ISL_95127 | Maharashtra | 2010-05-21 | A/Nanded/NIV29214/2010 | Other Database Import | Mullick,J.; Potdar,V.A. |
| EPI_ISL_79359 | Kerala | 2010-06-17 | A/India/007/2010 | Centers for Disease Control and Prevention | Garten,R. |
| EPI_ISL_96754 | Assam | 2010-06-17 | A/Assam/2590/2010 | Other Database Import | Biswas,D.; Lahan,M.; Yadav,K.; Borkakoty,B.; Mahanta,J. |
| EPI_ISL_161532 | Kerala | 2010-06-19 | A/Kerala/023/2010 | Other Database Import | Kumar,S.; Khare,S.; Saidullah,B.; Gandhok,I.; Rai,A. |
| EPI_ISL_79357 | Maharashtra | 2010-06-28 | A/India/5107/2010 | Centers for Disease Control and Prevention | Garten,R. |
| EPI_ISL_176801 | Jammu & Kashmir | 2010-07-19 | A/Jammu & Kashmir/021/2010 | Other Database Import | Kumar,S.; Khare,S.; Saidullah,B.; Rai,A. |
| EPI_ISL_206028 | Assam | 2010-07-20 | A/Assam/2668/2010 | Other Database Import | Biswas,D.; Sarmah,K.; Dutta,M.; Buragohain,M.; Yadav,K.; Borkakoty,B. |
| EPI_ISL_206029 | Assam | 2010-07-22 | A/Assam/2677/2010 | Other Database Import | Biswas,D.; Sarmah,K.; Dutta,M.; Buragohain,M.; Yadav,K.; Borkakoty,B. |
| EPI_ISL_190156 | Karnataka | 2010-07-23 | A/Bangalore/3934-28/2010 | Other Database Import | Thomas,M.; Desai,A.; Vasanthapuram,R. |
| EPI_ISL_190157 | Karnataka | 2010-07-27 | A/Bangalore/4080-23/2010 | Other Database Import | Thomas,M.; Desai,A.; Vasanthapuram,R. |
| EPI_ISL_190158 | Karnataka | 2010-07-28 | A/Bangalore/4161-22/2010 | Other Database Import | Thomas,M.; Desai,A.; Vasanthapuram,R. |
| EPI_ISL_190159 | Karnataka | 2010-07-28 | A/Bangalore/4163-30/2010 | Other Database Import | Thomas,M.; Desai,A.; Vasanthapuram,R. |
| EPI_ISL_161543 | Goa | 2010-08-07 | A/Goa/034/2010 | Other Database Import | Kumar,S.; Khare,S.; Saidullah,B.; Gandhok,I.; Rai,A. |
| EPI_ISL_190160 | Karnataka | 2010-08-07 | A/Bangalore/4895-25/2010 | Other Database Import | Thomas,M.; Desai,A.; Vasanthapuram,R. |
| EPI_ISL_190161 | Karnataka | 2010-08-08 | A/Bangalore/4976-17/2010 | Other Database Import | Thomas,M.; Desai,A.; Vasanthapuram,R. |
| EPI_ISL_167445 | Madhya Pradesh | 2010-08-09 | A/Jabalpur/543/2010 | Other Database Import | Barde,P.V.; Sahu,M.; Shukla,M.K.; Kori,B.K.; Sharma,R.K.; Singh,N. |
| EPI_ISL_190162 | Karnataka | 2010-08-09 | A/Bangalore/4985-07/2010 | Other Database Import | Thomas,M.; Desai,A.; Vasanthapuram,R. |
| EPI_ISL_190163 | Karnataka | 2010-08-11 | A/Bangalore/5274-20/2010 | Other Database Import | Thomas,M.; Desai,A.; Vasanthapuram,R. |
| EPI_ISL_190164 | Karnataka | 2010-08-11 | A/Bangalore/5279-29/2010 | Other Database Import | Thomas,M.; Desai,A.; Vasanthapuram,R. |
| EPI_ISL_190091 | Karnataka | 2010-08-13 | A/Bangalore/5531-27/2010 | Other Database Import | Thomas,M.; Desai,A.; Vasanthapuram,R. |
| EPI_ISL_190092 | Karnataka | 2010-08-13 | A/Bangalore/5532-05/2010 | Other Database Import | Thomas,M.; Desai,A.; Vasanthapuram,R. |
| EPI_ISL_190093 | Karnataka | 2010-08-13 | A/Bangalore/5674-21/2010 | Other Database Import | Thomas,M.; Desai,A.; Vasanthapuram,R. |
| EPI_ISL_190094 | Karnataka | 2010-08-14 | A/Bangalore/5712-24/2010 | Other Database Import | Thomas,M.; Desai,A.; Vasanthapuram,R. |
| EPI_ISL_161538 | Haryana | 2010-08-15 | A/Haryana/029/2010 | Other Database Import | Kumar,S.; Khare,S.; Saidullah,B.; Gandhok,I.; Rai,A. |
| EPI_ISL_190095 | Karnataka | 2010-08-17 | A/Bangalore/6106-16/2010 | Other Database Import | Thomas,M.; Desai,A.; Vasanthapuram,R. |
| EPI_ISL_161545 | Delhi | 2010-08-20 | A/Delhi/036/2010 | Other Database Import | Kumar,S.; Khare,S.; Saidullah,B.; Gandhok,I.; Rai,A. |
| EPI_ISL_161539 | Punjab | 2010-08-21 | A/Punjab/030/2010 | Other Database Import | Kumar,S.; Khare,S.; Saidullah,B.; Gandhok,I.; Rai,A. |
| EPI_ISL_161547 | Delhi | 2010-08-22 | A/Delhi/038/2010 | Other Database Import | Kumar,S.; Khare,S.; Saidullah,B.; Gandhok,I.; Rai,A. |
| EPI_ISL_190096 | Karnataka | 2010-08-23 | A/Bangalore/6846-26/2010 | Other Database Import | Thomas,M.; Desai,A.; Vasanthapuram,R. |
| EPI_ISL_161546 | Delhi | 2010-08-25 | A/Delhi/037/2010 | Other Database Import | Kumar,S.; Khare,S.; Saidullah,B.; Gandhok,I.; Rai,A. |
| EPI_ISL_161536 | Goa | 2010-08-26 | A/Goa/027/2010 | Other Database Import | Kumar,S.; Khare,S.; Saidullah,B.; Gandhok,I.; Rai,A. |
| EPI_ISL_104330 | Madhya Pradesh | 2010-09-09 | A/India/GWL_DSC/2010 | Other Database Import | Sharma,S.; Parida,M.M.; Shukla,J.; Joshi,G.; Dash,P.; Rao,P.V.L. |
| EPI_ISL_151998 | Madhya Pradesh | 2010-09-16 | A/Indore/1085/2010 | Other Database Import | Barde,P.V.; Sahu,M.; Shukla,M.K.; Kori,B.K.; Sahare,L. |
| EPI_ISL_161548 | Delhi | 2010-09-17 | A/Delhi/039/2010 | Other Database Import | Kumar,S.; Khare,S.; Saidullah,B.; Gandhok,I.; Rai,A. |
| EPI_ISL_161542 | Uttarakhand | 2010-09-24 | A/Uttrakhand/033/2010 | Other Database Import | Kumar,S.; Khare,S.; Saidullah,B.; Gandhok,I.; Rai,A. |
| EPI_ISL_201013 | Madhya Pradesh | 2010-09-30 | A/Jabalpur/1413/2010 | Other Database Import | Sahu,M.; Shukla,M.K.; Singh,N.; Barde,P.V. |
| EPI_ISL_161544 | Goa | 2010-10-09 | A/Goa/035/2010 | Other Database Import | Kumar,S.; Khare,S.; Saidullah,B.; Gandhok,I.; Rai,A. |
| EPI_ISL_161537 | Haryana | 2010-10-19 | A/Haryana/028/2010 | Other Database Import | Kumar,S.; Khare,S.; Saidullah,B.; Gandhok,I.; Rai,A. |
| EPI_ISL_161540 | Punjab | 2010-10-19 | A/Punjab/031/2010 | Other Database Import | Kumar,S.; Khare,S.; Saidullah,B.; Gandhok,I.; Rai,A. |
| EPI_ISL_201014 | Madhya Pradesh | 2010-11-04 | A/Bhopal/1544/2010 | Other Database Import | Sahu,M.; Shukla,M.K.; Singh,N.; Barde,P.V. |
| EPI_ISL_164487 | Jammu & Kashmir | 2011-01-03 | A/Srinigar/04/2011 | Centers for Disease Control and Prevention | Smith, Catherine B. |
| EPI_ISL_176838 | Jammu & Kashmir | 2011-01-20 | A/Jammu & Kashmir/044/2011 | Other Database Import | Kumar,S.; Khare,S.; Saidullah,B.; Rai,A. |
| EPI_ISL_176839 | Jammu & Kashmir | 2011-01-31 | A/Jammu & Kashmir/045/2011 | Other Database Import | Kumar,S.; Khare,S.; Saidullah,B.; Rai,A. |
| EPI_ISL_164489 | Jammu & Kashmir | 2011-02-14 | A/Srinigar/126/2011 | Centers for Disease Control and Prevention | Smith, Catherine B. |
| EPI_ISL_161549 | Punjab | 2011-02-21 | A/Punjab/040/2011 | Other Database Import | Kumar,S.; Khare,S.; Saidullah,B.; Gandhok,I.; Rai,A. |
| EPI_ISL_161560 | Delhi | 2011-02-24 | A/Delhi/051/2011 | Other Database Import | Kumar,S.; Khare,S.; Saidullah,B.; Gandhok,I.; Rai,A. |
| EPI_ISL_161561 | Delhi | 2011-02-24 | A/Delhi/052/2011 | Other Database Import | Kumar,S.; Khare,S.; Saidullah,B.; Gandhok,I.; Rai,A. |
| EPI_ISL_164488 | Jammu & Kashmir | 2011-02-25 | A/Srinigar/201/2011 | Centers for Disease Control and Prevention | Smith, Catherine B. |
| EPI_ISL_161565 | Delhi | 2011-03-05 | A/Delhi/056/2011 | Other Database Import | Kumar,S.; Khare,S.; Saidullah,B.; Rai,A. |
| EPI_ISL_164491 | Jammu & Kashmir | 2011-03-08 | A/Srinigar/234/2011 | Centers for Disease Control and Prevention | Smith, Catherine B. |
| EPI_ISL_161550 | Punjab | 2011-03-09 | A/Punjab/041/2011 | Other Database Import | Kumar,S.; Khare,S.; Saidullah,B.; Gandhok,I.; Rai,A. |
| EPI_ISL_161551 | Punjab | 2011-03-17 | A/Punjab/042/2011 | Other Database Import | Kumar,S.; Khare,S.; Saidullah,B.; Gandhok,I.; Rai,A. |
| EPI_ISL_161558 | Haryana | 2011-03-24 | A/Haryana/049/2011 | Other Database Import | Kumar,S.; Khare,S.; Saidullah,B.; Gandhok,I.; Rai,A. |
| EPI_ISL_161552 | Punjab | 2011-03-28 | A/Punjab/043/2011 | Other Database Import | Kumar,S.; Khare,S.; Saidullah,B.; Gandhok,I.; Rai,A. |
| EPI_ISL_161563 | Delhi | 2011-04-01 | A/Delhi/054/2011 | Other Database Import | Kumar,S.; Khare,S.; Saidullah,B.; Gandhok,I.; Rai,A. |
| EPI_ISL_161564 | Delhi | 2011-04-03 | A/Delhi/055/2011 | Other Database Import | Kumar,S.; Khare,S.; Saidullah,B.; Gandhok,I.; Rai,A. |
| EPI_ISL_164492 | Jammu & Kashmir | 2011-04-04 | A/Srinigar/385/2011 | Centers for Disease Control and Prevention | Smith, Catherine B. |
| EPI_ISL_167446 | Madhya Pradesh | 2011-04-19 | A/Bhopal/1613/2011 | Other Database Import | Barde,P.V.; Sahu,M.; Shukla,M.K.; Kori,B.K.; Sharma,R.K.; Singh,N. |
| EPI_ISL_161557 | Goa | 2011-05-19 | A/Goa/048/2011 | Other Database Import | Kumar,S.; Khare,S.; Saidullah,B.; Gandhok,I.; Rai,A. |
| EPI_ISL_161559 | Delhi | 2011-05-19 | A/Delhi/050/2011 | Other Database Import | Kumar,S.; Khare,S.; Saidullah,B.; Gandhok,I.; Rai,A. |
| EPI_ISL_93611 | Maharashtra | 2011-05-22 | A/India/4947/2011 | Centers for Disease Control and Prevention | Garten,R. |
| EPI_ISL_161556 | Goa | 2011-06-07 | A/Goa/047/2011 | Other Database Import | Kumar,S.; Khare,S.; Saidullah,B.; Gandhok,I.; Rai,A. |
| EPI_ISL_161555 | Goa | 2011-06-23 | A/Goa/046/2011 | Other Database Import | Kumar,S.; Khare,S.; Saidullah,B.; Gandhok,I.; Rai,A. |
| EPI_ISL_161562 | Delhi | 2011-06-23 | A/Delhi/053/2011 | Other Database Import | Kumar,S.; Khare,S.; Saidullah,B.; Gandhok,I.; Rai,A. |
| EPI_ISL_190097 | Karnataka | 2011-07-20 | A/Bangalore/1103-51/2011 | Other Database Import | Thomas,M.; Desai,A.; Vasanthapuram,R. |
| EPI_ISL_190098 | Karnataka | 2011-08-04 | A/Bangalore/1326-52/2011 | Other Database Import | Thomas,M.; Desai,A.; Vasanthapuram,R. |
| EPI_ISL_146133 | Maharashtra | 2011-08-10 | A/India/D1510037/2015 | Other Database Import | Potdar,V.; Dakhave,M.; Hinge,D.; Bhosle,P.; Koul,P.; Dar,L.; Raj,K.; Chadha,M. |
| EPI_ISL_132712 | Madhya Pradesh | 2011-08-24 | A/India/GWL-02/2011 | Other Database Import | Sharma,S. |
| EPI_ISL_132713 | Madhya Pradesh | 2011-08-24 | A/India/GWL-01/2011 | Other Database Import | Sharma,S. |
| EPI_ISL_190099 | Karnataka | 2011-09-09 | A/Bangalore/1808-53/2011 | Other Database Import | Thomas,M.; Desai,A.; Vasanthapuram,R. |
| EPI_ISL_190100 | Karnataka | 2011-09-23 | A/Bangalore/1974-55/2011 | Other Database Import | Thomas,M.; Desai,A.; Vasanthapuram,R. |
| EPI_ISL_146135 | Maharashtra | 2011-09-23 | A/India/Pun153225/2015 | Other Database Import | Potdar,V.A.; Hinge,D.D.; Harpale,P.M. |
| EPI_ISL_201015 | Madhya Pradesh | 2011-10-06 | A/Bhopal/1664/2011 | Other Database Import | Sahu,M.; Shukla,M.K.; Singh,N.; Barde,P.V. |
| EPI_ISL_103728 | Maharashtra | 2011-10-20 | A/India/5756/2011 | Centers for Disease Control and Prevention | Garten,R. |
| EPI_ISL_190101 | Karnataka | 2011-11-17 | A/Bangalore/2327-56/2011 | Other Database Import | Thomas,M.; Desai,A.; Vasanthapuram,R. |
| EPI_ISL_258671 | Maharashtra | 2011-11-22 | A/India/P153321/2015 | Other Database Import | Potdar,V.; Dakhave,M.; Hinge,D.; Bhosle,P.; Koul,P.; Dar,L.; Raj,K.; Chadha,M. |
| EPI_ISL_164494 | Jammu & Kashmir | 2011-12-22 | A/Srinigar/827/2011 | Centers for Disease Control and Prevention | Smith, Catherine B. |
| EPI_ISL_161582 | Goa | 2012-01-09 | A/Goa/073/2012 | Other Database Import | Kumar,S.; Khare,S.; Saidullah,B.; Gandhok,I.; Rai,A. |
| EPI_ISL_146119 | Maharashtra | 2012-02-10 | A/India/D1510044/2015 | Other Database Import | Potdar,V.; Dakhave,M.; Hinge,D.; Bhosle,P.; Koul,P.; Dar,L.; Raj,K.; Chadha,M. |
| EPI_ISL_161587 | Haryana | 2012-02-17 | A/Haryana/078/2012 | Other Database Import | Kumar,S.; Khare,S.; Saidullah,B.; Gandhok,I.; Rai,A. |
| EPI_ISL_164495 | Jammu & Kashmir | 2012-02-20 | A/Srinigar/1118/2011 | Centers for Disease Control and Prevention | Smith, Catherine B. |
| EPI_ISL_146139 | Maharashtra | 2012-02-24 | A/India/Jaipur153442/2015 | Other Database Import | Potdar,V.; Dakhave,M.; Hinge,D.; Bhosle,P.; Koul,P.; Dar,L.; Raj,K.; Chadha,M. |
| EPI_ISL_146141 | Maharashtra | 2012-02-24 | A/India/D1510040/2015 | Other Database Import | Potdar,V.; Dakhave,M.; Hinge,D.; Bhosle,P.; Koul,P.; Dar,L.; Raj,K.; Chadha,M. |
| EPI_ISL_258670 | Maharashtra | 2012-02-24 | A/India/P153388/2015 | Other Database Import | Potdar,V.; Dakhave,M.; Hinge,D.; Bhosle,P.; Koul,P.; Dar,L.; Raj,K.; Chadha,M. |
| EPI_ISL_146143 | Maharashtra | 2012-02-24 | A/India/P153389/2015 | Other Database Import | Potdar,V.; Dakhave,M.; Hinge,D.; Bhosle,P.; Koul,P.; Dar,L.; Raj,K.; Chadha,M. |
| EPI_ISL_146129 | Maharashtra | 2012-02-24 | A/Barwani/6024/2015 | Other Database Import | Sahu,M.; Shukla,M.K.; Singh,N.; Barde,P.V. |
| EPI_ISL_258672 | Maharashtra | 2012-02-27 | A/India/Srinagar1513274/2015 | Other Database Import | Potdar,V.; Dakhave,M.; Hinge,D.; Bhosle,P.; Koul,P.; Dar,L.; Raj,K.; Chadha,M. |
| EPI_ISL_258677 | Maharashtra | 2012-02-28 | A/India/Kol-3828/2015 | Other Database Import | Mukherjee,A.; Chawla-Sarkar,M. |
| EPI_ISL_146131 | Maharashtra | 2012-02-29 | A/India/D1510042/2015 | Other Database Import | Potdar,V.; Dakhave,M.; Hinge,D.; Bhosle,P.; Koul,P.; Dar,L.; Raj,K.; Chadha,M. |
| EPI_ISL_164496 | Jammu & Kashmir | 2012-03-05 | A/Srinigar/1186/2011 | Centers for Disease Control and Prevention | Smith, Catherine B. |
| EPI_ISL_258674 | Maharashtra | 2012-03-05 | A/India/P153681/2015 | Other Database Import | Potdar,V.; Dakhave,M.; Hinge,D.; Bhosle,P.; Koul,P.; Dar,L.; Raj,K.; Chadha,M. |
| EPI_ISL_258686 | Maharashtra | 2012-03-05 | A/India/8448/2015 (2015) | National Centre for Disease Control (NCDC) |  |
| EPI_ISL_258675 | Maharashtra | 2012-03-06 | A/India/Srinagar1510348/2015 | Other Database Import | Potdar,V.; Dakhave,M.; Hinge,D.; Bhosle,P.; Koul,P.; Dar,L.; Raj,K.; Chadha,M. |
| EPI_ISL_258689 | Maharashtra | 2012-03-07 | A/India/P1510348/2015 | National Institute of Infectious Diseases (NIID) | Takashita,Emi; Fujisaki,Seiichiro; Shirakura,Masayuki; Watanabe,Shinji; Odagiri,Takato |
| EPI_ISL_161574 | Delhi | 2012-03-09 | A/Delhi/065/2012 | Other Database Import | Kumar,S.; Khare,S.; Saidullah,B.; Gandhok,I.; Rai,A. |
| EPI_ISL_161573 | Delhi | 2012-03-10 | A/Delhi/064/2012 | Other Database Import | Kumar,S.; Khare,S.; Saidullah,B.; Gandhok,I.; Rai,A. |
| EPI_ISL_258680 | Maharashtra | 2012-03-13 | A/India/Srinagar1513279/2015 | Other Database Import | Potdar,V.; Dakhave,M.; Hinge,D.; Bhosle,P.; Koul,P.; Dar,L.; Raj,K.; Chadha,M. |
| EPI_ISL_258676 | Maharashtra | 2012-03-15 | A/India/DRDE_GWL703/2015 | Other Database Import | Parida,M.; Dash,P.K.; Kumar,J.S.; Joshi,G.; Tandel,K.; Sharma,S.; Srivastava,A.; Agarwal,A.; Saha,A.; Saraswat,S.; Karothia,D.; Malviya,V. |
| EPI_ISL_258688 | Maharashtra | 2012-03-16 | A/India/DRDE_GWL719/2015 | Other Database Import | Parida,M.; Dash,P.K.; Kumar,J.S.; Joshi,G.; Tandel,K.; Sharma,S.; Srivastava,A.; Agarwal,A.; Saha,A.; Saraswat,S.; Karothia,D.; Malviya,V. |
| EPI_ISL_258681 | Maharashtra | 2012-03-16 | A/India/DRDE_GWL721/2015 | Other Database Import | Parida,M.; Dash,P.K.; Kumar,J.S.; Joshi,G.; Tandel,K.; Sharma,S.; Srivastava,A.; Agarwal,A.; Saha,A.; Saraswat,S.; Karothia,D.; Malviya,V. |
| EPI_ISL_258673 | Maharashtra | 2012-03-17 | A/India/Kol-3846/2015 | Other Database Import | Mukherjee,A.; Chawla-Sarkar,M. |
| EPI_ISL_258666 | Maharashtra | 2012-03-17 | A/India/Kol-3959/2015 | Other Database Import | Mukherjee,A.; Chawla-Sarkar,M. |
| EPI_ISL_258690 | Maharashtra | 2012-03-21 | A/India/DRDE_GWL812/2015 | Other Database Import | Dash,P.K.; Pattabiraman,C.; Tandel,K.; Sharma,S.; Siddappa,S.; Kumar,J.; Gowda,M.; Krishna,S.; Parida,M.M. |
| EPI_ISL_258679 | Maharashtra | 2012-03-21 | A/India/D1510043/2015 | Other Database Import | Gurav,Y.K.; Chadha,M.S.; Tandale,B.V.; Potdar,V.A.; Pawar,S.D.; Shil,P.; Deoshatwar,A.R.; Aarthy,R.; Bhushan,A.; Dakhave,M.; Hinge,D.; Bhosle,P.; Koul,P.; Dar,L.; Raj,K. |
| EPI_ISL_258682 | Maharashtra | 2012-03-21 | A/India/P154479/2015 | Other Database Import | Potdar,V.; Dakhave,M.; Hinge,D.; Bhosle,P.; Koul,P.; Dar,L.; Raj,K.; Chadha,M. |
| EPI_ISL_258684 | Maharashtra | 2012-03-21 | A/India/Kol-S4163/2015 | Other Database Import | Mukherjee,A.; Chawla-Sarkar,M. |
| EPI_ISL_258687 | Maharashtra | 2012-03-21 | A/India/DRDE_GWL897/2015 | Other Database Import | Dash,P.K.; Pattabiraman,C.; Tandel,K.; Sharma,S.; Siddappa,S.; Kumar,J.; Gowda,M.; Krishna,S.; Parida,M.M. |
| EPI_ISL_258665 | Maharashtra | 2012-03-22 | A/India/DRDE_GWL812/2015 | Other Database Import | Parida,M.; Dash,P.K.; Kumar,J.S.; Joshi,G.; Tandel,K.; Sharma,S.; Srivastava,A.; Agarwal,A.; Saha,A.; Saraswat,S.; Karothia,D.; Malviya,V. |
| EPI_ISL_258685 | Maharashtra | 2012-03-22 | A/India/Kol-4040/2015 | Other Database Import | Mukherjee,A.; Chawla-Sarkar,M. |
| EPI_ISL_258683 | Maharashtra | 2012-03-22 | A/India/Kol-4122/2015 | Other Database Import | Mukherjee,A.; Chawla-Sarkar,M. |
| EPI_ISL_258667 | Maharashtra | 2012-03-22 | A/India/P154479/2015 | National Institute of Infectious Diseases (NIID) | Takashita,Emi; Fujisaki,Seiichiro; Shirakura,Masayuki; Watanabe,Shinji; Odagiri,Takato |
| EPI_ISL_167447 | Madhya Pradesh | 2012-03-26 | A/Bhopal/1697/2012 | Other Database Import | Barde,P.V.; Sahu,M.; Shukla,M.K.; Kori,B.K.; Sharma,R.K.; Singh,N. |
| EPI_ISL_164486 | Delhi | 2012-03-29 | A/Delhi/1966/2012 | Centers for Disease Control and Prevention | Smith, Catherine B. |
| EPI_ISL_258669 | Maharashtra | 2012-04-05 | A/India/DRDE_GWL897/2015 | Other Database Import | Parida,M.; Dash,P.K.; Kumar,J.S.; Joshi,G.; Tandel,K.; Sharma,S.; Srivastava,A.; Agarwal,A.; Saha,A.; Saraswat,S.; Karothia,D.; Malviya,V. |
| EPI_ISL_258678 | Maharashtra | 2012-04-07 | A/Betul/6515/2015 | Other Database Import | Sahu,M.; Shukla,M.K.; Singh,N.; Barde,P.V. |
| EPI_ISL_190102 | Karnataka | 2012-04-10 | A/Bangalore/597-39/2012 | Other Database Import | Thomas,M.; Desai,A.; Vasanthapuram,R. |
| EPI_ISL_190103 | Karnataka | 2012-04-12 | A/Bangalore/697-32/2012 | Other Database Import | Thomas,M.; Desai,A.; Vasanthapuram,R. |
| EPI_ISL_258706 | Kerala | 2012-04-16 | A/India/Kerala128941/2012 | Other Database Import | Potdar,V.; Dakhave,M.; Patil,K.; Chawala Sarkar,M.; B,A.; Koul,P.; Chadha,M. |
| EPI_ISL_258668 | Maharashtra | 2012-04-16 | A/India/DRDE_GWL84/2015 | Other Database Import | Dash,P.K.; Pattabiraman,C.; Tandel,K.; Sharma,S.; Siddappa,S.; Kumar,J.; Gowda,M.; Krishna,S.; Parida,M.M. |
| EPI_ISL_258707 | Kerala | 2012-04-19 | A/India/Kerala128942/2012 | Other Database Import | Potdar,V.; Dakhave,M.; Patil,K.; Chawala Sarkar,M.; B,A.; Koul,P.; Chadha,M. |
| EPI_ISL_258708 | Kerala | 2012-04-20 | A/India/Kerala128943/2012 | Other Database Import | Potdar,V.; Dakhave,M.; Patil,K.; Chawala Sarkar,M.; B,A.; Koul,P.; Chadha,M. |
| EPI_ISL_167448 | Madhya Pradesh | 2012-04-28 | A/Harda/1718/2012 | Other Database Import | Barde,P.V.; Sahu,M.; Shukla,M.K.; Kori,B.K.; Sharma,R.K.; Singh,N. |
| EPI_ISL_190104 | Karnataka | 2012-05-02 | A/Bangalore/1155-33/2012 | Other Database Import | Thomas,M.; Desai,A.; Vasanthapuram,R. |
| EPI_ISL_161580 | Goa | 2012-05-15 | A/Goa/071/2012 | Other Database Import | Kumar,S.; Khare,S.; Saidullah,B.; Gandhok,I.; Rai,A. |
| EPI_ISL_124939 | Maharashtra | 2012-05-17 | A/India/8557/2012 | Centers for Disease Control and Prevention | Garten,R. |
| EPI_ISL_161570 | Delhi | 2012-05-21 | A/Delhi/061/2012 | Other Database Import | Kumar,S.; Khare,S.; Saidullah,B.; Gandhok,I.; Rai,A. |
| EPI_ISL_190105 | Karnataka | 2012-05-26 | A/Bangalore/1428-35/2012 | Other Database Import | Thomas,M.; Desai,A.; Vasanthapuram,R. |
| EPI_ISL_190106 | Karnataka | 2012-06-02 | A/Bangalore/1491-34/2012 | Other Database Import | Thomas,M.; Desai,A.; Vasanthapuram,R. |
| EPI_ISL_128970 | Kerala | 2012-06-04 | A/India/447/2012 | Centers for Disease Control and Prevention | Garten,R. |
| EPI_ISL_161585 | Haryana | 2012-06-11 | A/Haryana/076/2012 | Other Database Import | Kumar,S.; Khare,S.; Saidullah,B.; Gandhok,I.; Rai,A. |
| EPI_ISL_258710 | Kerala | 2012-06-14 | A/India/Kerala1210448/2012 | Other Database Import | Potdar,V.; Dakhave,M.; Patil,K.; Chawala Sarkar,M.; B,A.; Koul,P.; Chadha,M. |
| EPI_ISL_129011 | Kerala | 2012-06-21 | A/India/449/2012 | Centers for Disease Control and Prevention | Garten,R. |
| EPI_ISL_258709 | Kerala | 2012-06-26 | A/India/Kerala1210449/2012 | Other Database Import | Potdar,V.; Dakhave,M.; Patil,K.; Chawala Sarkar,M.; B,A.; Koul,P.; Chadha,M. |
| EPI_ISL_258700 | West.Bengal | 2012-06-26 | A/India/P152246/2015 | Other Database Import | Potdar,V.; Dakhave,M.; Hinge,D.; Bhosle,P.; Koul,P.; Dar,L.; Raj,K.; Chadha,M. |
| EPI_ISL_258701 | West.Bengal | 2012-06-26 | A/India/P152434/2015 | Other Database Import | Potdar,V.; Dakhave,M.; Hinge,D.; Bhosle,P.; Koul,P.; Dar,L.; Raj,K.; Chadha,M. |
| EPI_ISL_258719 | Maharashtra | 2012-07-05 | A/India/D1510045/2015 | Other Database Import | Potdar,V.; Dakhave,M.; Hinge,D.; Bhosle,P.; Koul,P.; Dar,L.; Raj,K.; Chadha,M. |
| EPI_ISL_258716 | Maharashtra | 2012-07-05 | A/India/P154871/2015 | Other Database Import | Potdar,V.; Dakhave,M.; Hinge,D.; Bhosle,P.; Koul,P.; Dar,L.; Raj,K.; Chadha,M. |
| EPI_ISL_258717 | Maharashtra | 2012-07-06 | A/India/DRDE_GWL989/2015 | Other Database Import | Parida,M.; Dash,P.K.; Kumar,J.S.; Joshi,G.; Tandel,K.; Sharma,S.; Srivastava,A.; Agarwal,A.; Saha,A.; Saraswat,S.; Karothia,D.; Malviya,V. |
| EPI_ISL_258720 | Maharashtra | 2012-07-10 | A/India/D1510038/2015 | Other Database Import | Potdar,V.; Dakhave,M.; Hinge,D.; Bhosle,P.; Koul,P.; Dar,L.; Raj,K.; Chadha,M. |
| EPI_ISL_258702 | West.Bengal | 2012-07-11 | A/Dewas/4497/2015 | Other Database Import | Sahu,M.; Shukla,M.K.; Singh,N.; Barde,P.V. |
| EPI_ISL_167449 | Madhya Pradesh | 2012-07-13 | A/Jabalpur/1737/2012 | Other Database Import | Barde,P.V.; Sahu,M.; Shukla,M.K.; Kori,B.K.; Sharma,R.K.; Singh,N. |
| EPI_ISL_258713 | Maharashtra | 2012-07-17 | A/India/P153225/2015 | Other Database Import | Potdar,V.; Dakhave,M.; Hinge,D.; Bhosle,P.; Koul,P.; Dar,L.; Raj,K.; Chadha,M. |
| EPI_ISL_258703 | West.Bengal | 2012-07-20 | A/India/Srinagar1513278/2015 | Other Database Import | Potdar,V.; Dakhave,M.; Hinge,D.; Bhosle,P.; Koul,P.; Dar,L.; Raj,K.; Chadha,M. |
| EPI_ISL_258704 | West.Bengal | 2012-07-20 | A/India/Srinagar1513277/2015 | Other Database Import | Potdar,V.; Dakhave,M.; Hinge,D.; Bhosle,P.; Koul,P.; Dar,L.; Raj,K.; Chadha,M. |
| EPI_ISL_258718 | Maharashtra | 2012-07-20 | A/India/P153017/2015 | Other Database Import | Potdar,V.; Dakhave,M.; Hinge,D.; Bhosle,P.; Koul,P.; Dar,L.; Raj,K.; Chadha,M. |
| EPI_ISL_161566 | Delhi | 2012-07-26 | A/Delhi/057/2012 | Other Database Import | Kumar,S.; Khare,S.; Saidullah,B.; Gandhok,I.; Rai,A. |
| EPI_ISL_258715 | Maharashtra | 2012-07-26 | A/India/P153017/2015 | National Institute of Infectious Diseases (NIID) | Takashita,Emi; Fujisaki,Seiichiro; Shirakura,Masayuki; Watanabe,Shinji; Odagiri,Takato |
| EPI_ISL_190107 | Karnataka | 2012-07-27 | A/Bangalore/1980-42/2012 | Other Database Import | Thomas,M.; Desai,A.; Vasanthapuram,R. |
| EPI_ISL_190108 | Karnataka | 2012-07-30 | A/Bangalore/2008-43/2012 | Other Database Import | Thomas,M.; Desai,A.; Vasanthapuram,R. |
| EPI_ISL_258714 | Maharashtra | 2012-08-01 | A/India/D1510039/2015 | Other Database Import | Potdar,V.; Dakhave,M.; Hinge,D.; Bhosle,P.; Koul,P.; Dar,L.; Raj,K.; Chadha,M. |
| EPI_ISL_190109 | Karnataka | 2012-08-02 | A/Bangalore/2071-38/2012 | Other Database Import | Thomas,M.; Desai,A.; Vasanthapuram,R. |
| EPI_ISL_258725 | Maharashtra | 2012-08-04 | A/Ujjain/5448/2015 | Other Database Import | Sahu,M.; Shukla,M.K.; Singh,N.; Barde,P.V. |
| EPI_ISL_258712 | Maharashtra | 2012-08-12 | A/India/P153321/2015 | National Institute of Infectious Diseases (NIID) | Takashita,Emi; Fujisaki,Seiichiro; Shirakura,Masayuki; Watanabe,Shinji; Odagiri,Takato |
| EPI_ISL_190110 | Karnataka | 2012-08-14 | A/Bangalore/2285-44/2012 | Other Database Import | Thomas,M.; Desai,A.; Vasanthapuram,R. |
| EPI_ISL_161571 | Delhi | 2012-08-16 | A/Delhi/062/2012 | Other Database Import | Kumar,S.; Khare,S.; Saidullah,B.; Gandhok,I.; Rai,A. |
| EPI_ISL_167450 | Madhya Pradesh | 2012-08-16 | A/Jabalpur/1758/2012 | Other Database Import | Barde,P.V.; Sahu,M.; Shukla,M.K.; Kori,B.K.; Sharma,R.K.; Singh,N. |
| EPI_ISL_258721 | Maharashtra | 2012-08-17 | A/India/6691/2015 (H1N1) | National Centre for Disease Control (NCDC) |  |
| EPI_ISL_258711 | Maharashtra | 2012-08-23 | A/India/Pun153389/2015 | Other Database Import | Potdar,V.A.; Hinge,D.D.; Harpale,P.M. |
| EPI_ISL_258722 | Maharashtra | 2012-08-29 | A/India/Pun153388/2015 | Other Database Import | Potdar,V.A.; Hinge,D.D.; Harpale,P.M. |
| EPI_ISL_218340 | Maharashtra | 2012-08-30 | A/India/Nag1218589/2012 | Other Database Import | Potdar,V.A.; Dakhave,M.R. |
| EPI_ISL_190697 | Madhya Pradesh | 2012-08-31 | A/India/Gwl-06/2012 | Other Database Import | Sharma,S.; Dash,P.K.; Parida,M.M. |
| EPI_ISL_190111 | Karnataka | 2012-09-04 | A/Bangalore/2629-37/2012 | Other Database Import | Thomas,M.; Desai,A.; Vasanthapuram,R. |
| EPI_ISL_161569 | Delhi | 2012-09-13 | A/Delhi/060/2012 | Other Database Import | Kumar,S.; Khare,S.; Saidullah,B.; Gandhok,I.; Rai,A. |
| EPI_ISL_190112 | Karnataka | 2012-09-15 | A/Bangalore/2813-36/2012 | Other Database Import | Thomas,M.; Desai,A.; Vasanthapuram,R. |
| EPI_ISL_161575 | Delhi | 2012-09-24 | A/Delhi/066/2012 | Other Database Import | Kumar,S.; Khare,S.; Saidullah,B.; Gandhok,I.; Rai,A. |
| EPI_ISL_161572 | Delhi | 2012-09-25 | A/Delhi/063/2012 | Other Database Import | Kumar,S.; Khare,S.; Saidullah,B.; Gandhok,I.; Rai,A. |
| EPI_ISL_161576 | Delhi | 2012-09-25 | A/Delhi/067/2012 | Other Database Import | Kumar,S.; Khare,S.; Saidullah,B.; Gandhok,I.; Rai,A. |
| EPI_ISL_279405 | Rajasthan | 2012-09-28 | A/Jodhpur/3168/2012 | Other Database Import | Angel,B.; Angel,A.; Joshi,A.P.; Baharia,R.K.; Rathore,S.; Joshi,V. |
| EPI_ISL_161579 | Uttarakhand | 2012-10-04 | A/Uttrakhand/070/2012 | Other Database Import | Kumar,S.; Khare,S.; Saidullah,B.; Gandhok,I.; Rai,A. |
| EPI_ISL_133163 | Kerala | 2012-10-05 | A/India/1214/2012 | Centers for Disease Control and Prevention | Garten,R. |
| EPI_ISL_258694 | Maharashtra | 2012-10-08 | A/India/Srinagar1510349/2015 | Other Database Import | Potdar,V.; Dakhave,M.; Hinge,D.; Bhosle,P.; Koul,P.; Dar,L.; Raj,K.; Chadha,M. |
| EPI_ISL_161568 | Delhi | 2012-10-09 | A/Delhi/059/2012 | Other Database Import | Kumar,S.; Khare,S.; Saidullah,B.; Gandhok,I.; Rai,A. |
| EPI_ISL_161581 | Goa | 2012-10-09 | A/Goa/072/2012 | Other Database Import | Kumar,S.; Khare,S.; Saidullah,B.; Gandhok,I.; Rai,A. |
| EPI_ISL_190113 | Karnataka | 2012-10-15 | A/Bangalore/3361-46/2012 | Other Database Import | Thomas,M.; Desai,A.; Vasanthapuram,R. |
| EPI_ISL_258695 | Maharashtra | 2012-10-18 | A/India/8259/2015 (H1N1) | National Centre for Disease Control (NCDC) |  |
| EPI_ISL_190114 | Karnataka | 2012-10-20 | A/Bangalore/3432-40/2012 | Other Database Import | Thomas,M.; Desai,A.; Vasanthapuram,R. |
| EPI_ISL_258691 | Maharashtra | 2012-10-25 | A/India/D1510041/2015 | Other Database Import | Potdar,V.; Dakhave,M.; Hinge,D.; Bhosle,P.; Koul,P.; Dar,L.; Raj,K.; Chadha,M. |
| EPI_ISL_133153 | Maharashtra | 2012-11-10 | A/India/5416/2012 | Centers for Disease Control and Prevention | Garten,R. |
| EPI_ISL_161567 | Delhi | 2012-11-14 | A/Delhi/058/2012 | Other Database Import | Kumar,S.; Khare,S.; Saidullah,B.; Gandhok,I.; Rai,A. |
| EPI_ISL_258726 | Kerala | 2012-11-14 | A/India/Kerala132655/2012 | Other Database Import | Potdar,V.; Dakhave,M.; Patil,K.; Chawala Sarkar,M.; B,A.; Koul,P.; Chadha,M. |
| EPI_ISL_258696 | Maharashtra | 2012-11-17 | A/India/Pun153793/2015 | Other Database Import | Potdar,V.A.; Hinge,D.D.; Harpale,P.M. |
| EPI_ISL_252693 | Tamil Nadu | 2012-11-17 | A/Assam/RMRC_605/2016 | Other Database Import | Borkakoty,B.; Jakharia,A.; Sarmah,K.; Hazarika,R.; Biswas,D.; Mahanta,J. |
| EPI_ISL_161577 | Delhi | 2012-11-20 | A/Delhi/068/2012 | Other Database Import | Kumar,S.; Khare,S.; Saidullah,B.; Gandhok,I.; Rai,A. |
| EPI_ISL_161586 | Haryana | 2012-11-20 | A/Haryana/077/2012 | Other Database Import | Kumar,S.; Khare,S.; Saidullah,B.; Gandhok,I.; Rai,A. |
| EPI_ISL_258697 | Jammu & Kashmir | 2012-11-22 | A/India/Srinagar131670/2012 | Other Database Import | Potdar,V.; Dakhave,M.; Patil,K.; Chawala Sarkar,M.; B,A.; Koul,P.; Chadha,M. |
| EPI_ISL_190115 | Karnataka | 2012-11-24 | A/Bangalore/3666-41/2012 | Other Database Import | Thomas,M.; Desai,A.; Vasanthapuram,R. |
| EPI_ISL_252692 | Tamil Nadu | 2012-11-25 | A/Assam/RMRC_598/2016 | Other Database Import | Borkakoty,B.; Jakharia,A.; Sarmah,K.; Hazarika,R.; Biswas,D.; Mahanta,J. |
| EPI_ISL_135817 | Jammu & Kashmir | 2012-11-26 | A/India/2019/2012 | Centers for Disease Control and Prevention | Garten,R. |
| EPI_ISL_258698 | Jammu & Kashmir | 2012-11-26 | A/India/Srinagar131672/2012 | Other Database Import | Potdar,V.; Dakhave,M.; Patil,K.; Chawala Sarkar,M.; B,A.; Koul,P.; Chadha,M. |
| EPI_ISL_133537 | Jammu & Kashmir | 2012-12-03 | A/India/2055/2012 | Centers for Disease Control and Prevention | Garten,R. |
| EPI_ISL_134024 | Jammu & Kashmir | 2012-12-03 | A/India/2055/2012 | Centers for Disease Control and Prevention | Garten,R. |
| EPI_ISL_134420 | Jammu & Kashmir | 2012-12-03 | A/India/2055/2012 | Centers for Disease Control and Prevention | Garten,R. |
| EPI_ISL_134026 | Jammu & Kashmir | 2012-12-06 | A/India/2080/2012 | Centers for Disease Control and Prevention | Garten,R. |
| EPI_ISL_134839 | Jammu & Kashmir | 2012-12-06 | A/India/2080/2012 | Centers for Disease Control and Prevention | Garten,R. |
| EPI_ISL_134854 | Jammu & Kashmir | 2012-12-06 | A/India/2080/2012 | Centers for Disease Control and Prevention | Garten,R. |
| EPI_ISL_279401 | Rajasthan | 2012-12-10 | A/Jodhpur/3248/2012 | Other Database Import | Angel,B.; Angel,A.; Joshi,A.P.; Baharia,R.K.; Rathore,S.; Joshi,V. |
| EPI_ISL_279403 | Rajasthan | 2012-12-10 | A/Jodhpur/3240/2012 | Other Database Import | Angel,B.; Angel,A.; Joshi,A.P.; Baharia,R.K.; Rathore,S.; Joshi,V. |
| EPI_ISL_258692 | Maharashtra | 2012-12-11 | A/India/P153793/2015 | Other Database Import | Gurav,Y.K.; Chadha,M.S.; Tandale,B.V.; Potdar,V.A.; Pawar,S.D.; Shil,P.; Deoshatwar,A.R.; Aarthy,R.; Bhushan,A.; Dakhave,M.; Hinge,D.; Bhosle,P.; Koul,P.; Dar,L.; Raj,K. |
| EPI_ISL_258699 | Maharashtra | 2012-12-14 | A/India/DRDE_GWL672/2015 | Other Database Import | Dash,P.K.; Pattabiraman,C.; Tandel,K.; Sharma,S.; Siddappa,S.; Kumar,J.; Gowda,M.; Krishna,S.; Parida,M.M. |
| EPI_ISL_134423 | Jammu & Kashmir | 2012-12-17 | A/India/2150/2012 | Centers for Disease Control and Prevention | Garten,R. |
| EPI_ISL_134763 | Jammu & Kashmir | 2012-12-17 | A/India/2150/2012 | Centers for Disease Control and Prevention | Garten,R. |
| EPI_ISL_133530 | Jammu & Kashmir | 2012-12-19 | A/India/2169/2012 | Centers for Disease Control and Prevention | Garten,R. |
| EPI_ISL_134837 | Jammu & Kashmir | 2012-12-19 | A/India/2171/2012 | Centers for Disease Control and Prevention | Garten,R. |
| EPI_ISL_161578 | Delhi | 2012-12-19 | A/Delhi/069/2012 | Other Database Import | Kumar,S.; Khare,S.; Saidullah,B.; Gandhok,I.; Rai,A. |
| EPI_ISL_133532 | Jammu & Kashmir | 2012-12-20 | A/India/2177/2012 | Centers for Disease Control and Prevention | Garten,R. |
| EPI_ISL_133534 | Jammu & Kashmir | 2012-12-21 | A/India/2183/2012 | Centers for Disease Control and Prevention | Garten,R. |
| EPI_ISL_134023 | Jammu & Kashmir | 2012-12-21 | A/India/2181/2012 | Centers for Disease Control and Prevention | Garten,R. |
| EPI_ISL_134838 | Jammu & Kashmir | 2012-12-21 | A/India/2181/2012 | Centers for Disease Control and Prevention | Garten,R. |
| EPI_ISL_134855 | Jammu & Kashmir | 2012-12-21 | A/India/2181/2012 | Centers for Disease Control and Prevention | Garten,R. |
| EPI_ISL_133536 | Jammu & Kashmir | 2012-12-22 | A/India/2192/2012 | Centers for Disease Control and Prevention | Garten,R. |
| EPI_ISL_134027 | Jammu & Kashmir | 2012-12-22 | A/India/2192/2012 | Centers for Disease Control and Prevention | Garten,R. |
| EPI_ISL_134437 | Jammu & Kashmir | 2012-12-22 | A/India/2192/2012 | Centers for Disease Control and Prevention | Garten,R. |
| EPI_ISL_134840 | Jammu & Kashmir | 2012-12-22 | A/India/2192/2012 | Centers for Disease Control and Prevention | Garten,R. |
| EPI_ISL_138692 | Jammu & Kashmir | 2012-12-22 | A/India/2192/2012 | Centers for Disease Control and Prevention | Garten,R. |
| EPI_ISL_134427 | Jammu & Kashmir | 2012-12-24 | A/India/2205/2012 | Centers for Disease Control and Prevention | Garten,R. |
| EPI_ISL_134846 | Jammu & Kashmir | 2012-12-24 | A/India/2205/2012 | Centers for Disease Control and Prevention | Garten,R. |
| EPI_ISL_134025 | Jammu & Kashmir | 2012-12-25 | A/India/2227/2012 | Centers for Disease Control and Prevention | Garten,R. |
| EPI_ISL_134856 | Jammu & Kashmir | 2012-12-25 | A/India/2227/2012 | Centers for Disease Control and Prevention | Garten,R. |
| EPI_ISL_258693 | Maharashtra | 2012-12-29 | A/India/P153926/2015 | Other Database Import | Gurav,Y.K.; Chadha,M.S.; Tandale,B.V.; Potdar,V.A.; Pawar,S.D.; Shil,P.; Deoshatwar,A.R.; Aarthy,R.; Bhushan,A.; Dakhave,M.; Hinge,D.; Bhosle,P.; Koul,P.; Dar,L.; Raj,K. |
| EPI_ISL_190116 | Karnataka | 2012-12-31 | A/Bangalore/3925-45/2012 | Other Database Import | Thomas,M.; Desai,A.; Vasanthapuram,R. |
| EPI_ISL_161588 | Haryana | 2013-01-02 | A/Haryana/079/2013 | Other Database Import | Kumar,S.; Khare,S.; Saidullah,B.; Gandhok,I.; Rai,A. |
| EPI_ISL_218352 | Maharashtra | 2013-01-02 | A/India/Pun13102/2013 | Other Database Import | Potdar,V.A.; Dakhave,M.R. |
| EPI_ISL_218387 | Maharashtra | 2013-01-02 | A/India/Pun1338/2013 | Other Database Import | Potdar,V.A.; Dakhave,M.R. |
| EPI_ISL_218401 | Maharashtra | 2013-01-02 | A/India/Pun1364/2013 | Other Database Import | Potdar,V.A.; Dakhave,M.R. |
| EPI_ISL_218379 | Maharashtra | 2013-01-08 | A/India/Pun13253/2013 | Other Database Import | Potdar,V.A.; Dakhave,M.R. |
| EPI_ISL_218381 | Maharashtra | 2013-01-08 | A/India/Pun13269/2013 | Other Database Import | Potdar,V.A.; Dakhave,M.R. |
| EPI_ISL_218394 | Maharashtra | 2013-01-10 | A/India/Pun13565/2013 | Other Database Import | Potdar,V.A.; Dakhave,M.R. |
| EPI_ISL_218395 | Maharashtra | 2013-01-11 | A/India/Pun13602/2013 | Other Database Import | Potdar,V.A.; Dakhave,M.R. |
| EPI_ISL_218396 | Maharashtra | 2013-01-11 | A/India/Pun13605/2013 | Other Database Import | Potdar,V.A.; Dakhave,M.R. |
| EPI_ISL_218407 | Rajasthan | 2013-01-12 | A/India/Jai133302/2013 | Other Database Import | Potdar,V.A.; Dakhave,M.R. |
| EPI_ISL_218344 | Maharashtra | 2013-01-14 | A/India/Nag132465/2013 | Other Database Import | Potdar,V.A.; Dakhave,M.R. |
| EPI_ISL_218400 | Maharashtra | 2013-01-15 | A/India/Pun13633/2013 | Other Database Import | Potdar,V.A.; Dakhave,M.R. |
| EPI_ISL_201016 | Madhya Pradesh | 2013-01-19 | A/Ujjain/2558/2013 | Other Database Import | Sahu,M.; Shukla,M.K.; Singh,N.; Barde,P.V. |
| EPI_ISL_218404 | Maharashtra | 2013-01-19 | A/India/Pun13886/2013 | Other Database Import | Potdar,V.A.; Dakhave,M.R. |
| EPI_ISL_161590 | Haryana | 2013-01-22 | A/Haryana/081/2013 | Other Database Import | Kumar,S.; Khare,S.; Saidullah,B.; Gandhok,I.; Rai,A. |
| EPI_ISL_218405 | Maharashtra | 2013-01-22 | A/India/Pun13940/2013 | Other Database Import | Potdar,V.A.; Dakhave,M.R. |
| EPI_ISL_218406 | Maharashtra | 2013-01-24 | A/India/Pun13990/2013 | Other Database Import | Potdar,V.A.; Dakhave,M.R. |
| EPI_ISL_218354 | Maharashtra | 2013-01-25 | A/India/Pun131046/2013 | Other Database Import | Potdar,V.A.; Dakhave,M.R. |
| EPI_ISL_164782 | Maharashtra | 2013-01-27 | A/India/1027/2013 | Centers for Disease Control and Prevention | Garten,R. |
| EPI_ISL_218353 | Maharashtra | 2013-01-27 | A/India/Pun131027/2013 | Other Database Import | Potdar,V.A.; Dakhave,M.R. |
| EPI_ISL_153079 | Maharashtra | 2013-01-27 | A/India/DRDE_GWL989/2015 | Other Database Import | Dash,P.K.; Pattabiraman,C.; Tandel,K.; Sharma,S.; Siddappa,S.; Kumar,J.; Gowda,M.; Krishna,S.; Parida,M.M. |
| EPI_ISL_279398 | Rajasthan | 2013-01-30 | A/Bikaner/3362/2013 | Other Database Import | Angel,B.; Angel,A.; Joshi,A.P.; Baharia,R.K.; Rathore,S.; Joshi,V. |
| EPI_ISL_146123 | Maharashtra | 2013-01-31 | A/India/Nag132467/2013 | Other Database Import | Potdar,V.A.; Dakhave,M.R.; Patil,K.N.; Kadam,A.A.; Mullick,J.; Chadha,M.S. |
| EPI_ISL_161595 | Delhi | 2013-01-31 | A/Delhi/086/2013 | Other Database Import | Kumar,S.; Khare,S.; Saidullah,B.; Gandhok,I.; Rai,A. |
| EPI_ISL_218345 | Maharashtra | 2013-01-31 | A/India/Nag132467/2013 | Other Database Import | Potdar,V.A.; Dakhave,M.R. |
| EPI_ISL_218346 | Jammu & Kashmir | 2013-02-06 | A/India/Sri132838/2013 | Other Database Import | Potdar,V.A.; Dakhave,M.R. |
| EPI_ISL_218337 | Delhi | 2013-02-08 | A/India/Del136188/2013 | Other Database Import | Potdar,V.A.; Dakhave,M.R. |
| EPI_ISL_218347 | Jammu & Kashmir | 2013-02-08 | A/India/Sri132844/2013 | Other Database Import | Potdar,V.A.; Dakhave,M.R. |
| EPI_ISL_218408 | Rajasthan | 2013-02-10 | A/India/Jai133312/2013 | Other Database Import | Potdar,V.A.; Dakhave,M.R. |
| EPI_ISL_218366 | Maharashtra | 2013-02-11 | A/India/Pun131634/2013 | Other Database Import | Potdar,V.A.; Dakhave,M.R. |
| EPI_ISL_218377 | Maharashtra | 2013-02-13 | A/India/Pun131721/2013 | Other Database Import | Potdar,V.A.; Dakhave,M.R. |
| EPI_ISL_218368 | Maharashtra | 2013-02-14 | A/India/Pun131723/2013 | Other Database Import | Potdar,V.A.; Dakhave,M.R. |
| EPI_ISL_143682 | Maharashtra | 2013-02-15 | A/India/1343/2013 | Centers for Disease Control and Prevention | Garten,R. |
| EPI_ISL_145638 | Maharashtra | 2013-02-15 | A/India/1343/2013 | Centers for Disease Control and Prevention | Garten,R. |
| EPI_ISL_218369 | Maharashtra | 2013-02-15 | A/India/Pun131740/2013 | Other Database Import | Potdar,V.A.; Dakhave,M.R. |
| EPI_ISL_218370 | Maharashtra | 2013-02-15 | A/India/Pun131845/2013 | Other Database Import | Potdar,V.A.; Dakhave,M.R. |
| EPI_ISL_146125 | Maharashtra | 2013-02-15 | A/India/P154866/2015 | Other Database Import | Gurav,Y.K.; Chadha,M.S.; Tandale,B.V.; Potdar,V.A.; Pawar,S.D.; Shil,P.; Deoshatwar,A.R.; Aarthy,R.; Bhushan,A.; Dakhave,M.; Hinge,D.; Bhosle,P.; Koul,P.; Dar,L.; Raj,K. |
| EPI_ISL_161596 | Delhi | 2013-02-17 | A/Delhi/087/2013 | Other Database Import | Kumar,S.; Khare,S.; Saidullah,B.; Gandhok,I.; Rai,A. |
| EPI_ISL_161591 | Uttarakhand | 2013-02-18 | A/Uttrakhand/082/2013 | Other Database Import | Kumar,S.; Khare,S.; Saidullah,B.; Gandhok,I.; Rai,A. |
| EPI_ISL_161597 | Delhi | 2013-02-18 | A/Delhi/088/2013 | Other Database Import | Kumar,S.; Khare,S.; Saidullah,B.; Gandhok,I.; Rai,A. |
| EPI_ISL_218375 | Maharashtra | 2013-02-18 | A/India/Pun132194/2013 | Other Database Import | Potdar,V.A.; Dakhave,M.R. |
| EPI_ISL_146127 | Maharashtra | 2013-02-18 | A/India/Kol-S4481/2015 | Other Database Import | Mukherjee,A.; Chawla-Sarkar,M. |
| EPI_ISL_218350 | Maharashtra | 2013-02-19 | A/India/Pun132042/2013 | Other Database Import | Potdar,V.A.; Dakhave,M.R. |
| EPI_ISL_176867 | Jammu & Kashmir | 2013-02-20 | A/Jammu & Kashmir/085/2013 | Other Database Import | Kumar,S.; Khare,S.; Saidullah,B.; Rai,A. |
| EPI_ISL_218409 | Rajasthan | 2013-02-20 | A/India/Jai133321/2013 | Other Database Import | Potdar,V.A.; Dakhave,M.R. |
| EPI_ISL_161592 | Uttarakhand | 2013-02-22 | A/Uttrakhand/083/2013 | Other Database Import | Kumar,S.; Khare,S.; Saidullah,B.; Gandhok,I.; Rai,A. |
| EPI_ISL_161598 | Delhi | 2013-02-22 | A/Delhi/089/2013 | Other Database Import | Kumar,S.; Khare,S.; Saidullah,B.; Gandhok,I.; Rai,A. |
| EPI_ISL_176866 | Jammu & Kashmir | 2013-02-24 | A/Jammu & Kashmir/084/2013 | Other Database Import | Kumar,S.; Khare,S.; Saidullah,B.; Rai,A. |
| EPI_ISL_218351 | Maharashtra | 2013-02-25 | A/India/Pun132446/2013 | Other Database Import | Potdar,V.A.; Dakhave,M.R. |
| EPI_ISL_218378 | Maharashtra | 2013-02-25 | A/India/Pun132376/2013 | Other Database Import | Potdar,V.A.; Dakhave,M.R. |
| EPI_ISL_218410 | Rajasthan | 2013-02-27 | A/India/Jai133330/2013 | Other Database Import | Potdar,V.A.; Dakhave,M.R. |
| EPI_ISL_218380 | Maharashtra | 2013-02-28 | A/India/Pun132598/2013 | Other Database Import | Potdar,V.A.; Dakhave,M.R. |
| EPI_ISL_218382 | Maharashtra | 2013-03-02 | A/India/Pun132733/2013 | Other Database Import | Potdar,V.A.; Dakhave,M.R. |
| EPI_ISL_218334 | Delhi | 2013-03-08 | A/India/Del136199/2013 | Other Database Import | Potdar,V.A.; Dakhave,M.R. |
| EPI_ISL_218383 | Maharashtra | 2013-03-08 | A/India/Pun132978/2013 | Other Database Import | Potdar,V.A.; Dakhave,M.R. |
| EPI_ISL_143241 | Jammu & Kashmir | 2013-03-13 | A/India/4180/2013 | Centers for Disease Control and Prevention | Garten,R. |
| EPI_ISL_218349 | Jammu & Kashmir | 2013-03-13 | A/India/Sri134180/2013 | Other Database Import | Potdar,V.A.; Dakhave,M.R. |
| EPI_ISL_218384 | Maharashtra | 2013-03-14 | A/India/Pun133286/2013 | Other Database Import | Potdar,V.A.; Dakhave,M.R. |
| EPI_ISL_218385 | Maharashtra | 2013-03-18 | A/India/Pun133435/2013 | Other Database Import | Potdar,V.A.; Dakhave,M.R. |
| EPI_ISL_218348 | Jammu & Kashmir | 2013-03-20 | A/India/Sri134179/2013 | Other Database Import | Potdar,V.A.; Dakhave,M.R. |
| EPI_ISL_218386 | Maharashtra | 2013-03-22 | A/India/Pun133680/2013 | Other Database Import | Potdar,V.A.; Dakhave,M.R. |
| EPI_ISL_218388 | Maharashtra | 2013-03-26 | A/India/Pun133840/2013 | Other Database Import | Potdar,V.A.; Dakhave,M.R. |
| EPI_ISL_167451 | Madhya Pradesh | 2013-03-29 | A/Indore/2820/2013 | Other Database Import | Barde,P.V.; Sahu,M.; Shukla,M.K.; Kori,B.K.; Sharma,R.K.; Singh,N. |
| EPI_ISL_218332 | Kerala | 2013-04-01 | A/India/Alp135124/2013 | Other Database Import | Potdar,V.A.; Dakhave,M.R. |
| EPI_ISL_218333 | Kerala | 2013-04-03 | A/India/Alp135125/2013 | Other Database Import | Potdar,V.A.; Dakhave,M.R. |
| EPI_ISL_143240 | Maharashtra | 2013-04-05 | A/India/2213/2013 | Centers for Disease Control and Prevention | Garten,R. |
| EPI_ISL_218390 | Maharashtra | 2013-04-07 | A/India/Pun134512/2013 | Other Database Import | Potdar,V.A.; Dakhave,M.R. |
| EPI_ISL_218391 | Maharashtra | 2013-04-15 | A/India/Pun134666/2013 | Other Database Import | Potdar,V.A.; Dakhave,M.R. |
| EPI_ISL_218392 | Maharashtra | 2013-04-18 | A/India/Pun134828/2013 | Other Database Import | Potdar,V.A.; Dakhave,M.R. |
| EPI_ISL_218393 | Maharashtra | 2013-04-29 | A/India/Pun135091/2013 | Other Database Import | Potdar,V.A.; Dakhave,M.R. |
| EPI_ISL_218399 | Maharashtra | 2013-04-29 | A/India/Pun136180/2013 | Other Database Import | Potdar,V.A.; Dakhave,M.R. |
| EPI_ISL_161589 | Haryana | 2013-05-02 | A/Haryana/080/2013 | Other Database Import | Kumar,S.; Khare,S.; Saidullah,B.; Gandhok,I.; Rai,A. |
| EPI_ISL_218397 | Maharashtra | 2013-05-10 | A/India/Pun136169/2013 | Other Database Import | Potdar,V.A.; Dakhave,M.R. |
| EPI_ISL_218398 | Maharashtra | 2013-05-13 | A/India/Pun136171/2013 | Other Database Import | Potdar,V.A.; Dakhave,M.R. |
| EPI_ISL_167452 | Madhya Pradesh | 2013-05-15 | A/Itarsi/2934/2013 | Other Database Import | Barde,P.V.; Sahu,M.; Shukla,M.K.; Kori,B.K.; Sharma,R.K.; Singh,N. |
| EPI_ISL_151999 | Madhya Pradesh | 2013-07-03 | A/Indore/2683/2013 | Other Database Import | Barde,P.V.; Sahu,M.; Shukla,M.K.; Kori,B.K.; Sahare,L. |
| EPI_ISL_190698 | Madhya Pradesh | 2013-07-08 | A/India/GWL-13/2013 | Other Database Import | Sharma,S.; Joshi,G.; Dash,P.K.; Parida,M.M. |
| EPI_ISL_218356 | Maharashtra | 2013-07-16 | A/India/Pun1311101/2013 | Other Database Import | Potdar,V.A.; Dakhave,M.R. |
| EPI_ISL_218342 | Maharashtra | 2013-07-19 | A/India/Nag1320058/2013 | Other Database Import | Potdar,V.A.; Dakhave,M.R. |
| EPI_ISL_218367 | Maharashtra | 2013-07-20 | A/India/Pun1311648/2013 | Other Database Import | Potdar,V.A.; Dakhave,M.R. |
| EPI_ISL_218358 | Maharashtra | 2013-07-22 | A/India/Pun1311704/2013 | Other Database Import | Potdar,V.A.; Dakhave,M.R. |
| EPI_ISL_150294 | Maharashtra | 2013-07-26 | A/India/1968/2013 | Centers for Disease Control and Prevention | Garten,R. |
| EPI_ISL_218359 | Maharashtra | 2013-07-26 | A/India/Pun1311968/2013 | Other Database Import | Potdar,V.A.; Dakhave,M.R. |
| EPI_ISL_164374 | Maharashtra | 2013-07-29 | A/India/2167/2013 | Centers for Disease Control and Prevention | Garten,R. |
| EPI_ISL_218360 | Maharashtra | 2013-07-29 | A/India/Pun1312095/2013 | Other Database Import | Potdar,V.A.; Dakhave,M.R. |
| EPI_ISL_218361 | Maharashtra | 2013-07-29 | A/India/Pun1312167/2013 | Other Database Import | Potdar,V.A.; Dakhave,M.R. |
| EPI_ISL_218363 | Maharashtra | 2013-08-07 | A/India/Pun1312848/2013 | Other Database Import | Potdar,V.A.; Dakhave,M.R. |
| EPI_ISL_218364 | Maharashtra | 2013-08-08 | A/India/Pun1313346/2013 | Other Database Import | Potdar,V.A.; Dakhave,M.R. |
| EPI_ISL_218365 | Maharashtra | 2013-08-11 | A/India/Pun1313521/2013 | Other Database Import | Potdar,V.A.; Dakhave,M.R. |
| EPI_ISL_218343 | Maharashtra | 2013-08-22 | A/India/Nag1320061/2013 | Other Database Import | Potdar,V.A.; Dakhave,M.R. |
| EPI_ISL_150164 | Maharashtra | 2013-09-07 | A/India/3743/2013 | Centers for Disease Control and Prevention | Garten,R. |
| EPI_ISL_218371 | Maharashtra | 2013-09-10 | A/India/Pun1318508/2013 | Other Database Import | Potdar,V.A.; Dakhave,M.R. |
| EPI_ISL_218372 | Maharashtra | 2013-09-15 | A/India/Pun1319260/2013 | Other Database Import | Potdar,V.A.; Dakhave,M.R. |
| EPI_ISL_218373 | Maharashtra | 2013-09-30 | A/India/Pun1320517/2013 | Other Database Import | Potdar,V.A.; Dakhave,M.R. |
| EPI_ISL_218374 | Maharashtra | 2013-10-07 | A/India/Pun1321025/2013 | Other Database Import | Potdar,V.A.; Dakhave,M.R. |
| EPI_ISL_218376 | Maharashtra | 2013-11-18 | A/India/Pun132318/2013 | Other Database Import | Potdar,V.A.; Dakhave,M.R. |
| EPI_ISL_218417 | Maharashtra | 2014-01-18 | A/India/Pun14549/2014 | Other Database Import | Potdar,V.A.; Dakhave,M.R. |
| EPI_ISL_218418 | Maharashtra | 2014-01-21 | A/India/Pun14584/2014 | Other Database Import | Potdar,V.A.; Dakhave,M.R. |
| EPI_ISL_195926 | Delhi | 2014-02-05 | A/India/159/2014 (H1N1) | National Centre for Disease Control (NCDC) | Datt,T. |
| EPI_ISL_218414 | Maharashtra | 2014-02-10 | A/India/Pun141194/2014 | Other Database Import | Potdar,V.A.; Dakhave,M.R. |
| EPI_ISL_218338 | Delhi | 2014-03-04 | A/India/Del136197/2014 | Other Database Import | Potdar,V.A.; Dakhave,M.R. |
| EPI_ISL_164784 | Maharashtra | 2014-03-06 | A/India/6427/2014 | Centers for Disease Control and Prevention | Garten,R. |
| EPI_ISL_218421 | Kerala | 2014-03-11 | A/India/Alp1410359/2014 | Other Database Import | Potdar,V.A.; Dakhave,M.R. |
| EPI_ISL_218425 | Tamil Nadu | 2014-03-14 | A/Mysore/MCVRAF7729/2017 | Other Database Import | Jagadesh,A.; Arunkumar,G. |
| EPI_ISL_237083 | Kerala | 2014-04-01 | A/Kerala/RGCB140815/2014 | Other Database Import | Jones Palakkat,S.; Chirundodh,D.V.; Sujatha,A.S.; Fettle,A.; Jacob,J.; Pillai,M.R. |
| EPI_ISL_218426 | Tamil Nadu | 2014-05-02 | A/Mysore/MCVRAF7736/2017 | Other Database Import | Jagadesh,A.; Arunkumar,G. |
| EPI_ISL_164370 | Maharashtra | 2014-05-24 | A/India/5964/2014 | Centers for Disease Control and Prevention | Garten,R. |
| EPI_ISL_218419 | Maharashtra | 2014-06-03 | A/India/Pun146427/2014 | Other Database Import | Potdar,V.A.; Dakhave,M.R. |
| EPI_ISL_237084 | Kerala | 2014-06-09 | A/Kerala/RGCB141437/2014 | Other Database Import | Jones Palakkat,S.; Chirundodh,D.V.; Sujatha,A.S.; Fettle,A.; Jacob,J.; Pillai,M.R. |
| EPI_ISL_217003 | West Bengal | 2015 | A/India/Kol-3527/2015 | Other Database Import | Mukherjee,A.; Chawla-Sarkar,M. |
| EPI_ISL_217004 | West Bengal | 2015 | A/India/Kol-4628/2015 | Other Database Import | Mukherjee,A.; Chawla-Sarkar,M. |
| EPI_ISL_217005 | West Bengal | 2015 | A/India/Kol-4632/2015 | Other Database Import | Mukherjee,A.; Chawla-Sarkar,M. |
| EPI_ISL_217006 | West Bengal | 2015 | A/India/Kol-4651/2015 | Other Database Import | Mukherjee,A.; Chawla-Sarkar,M. |
| EPI_ISL_217007 | West Bengal | 2015 | A/India/Kol-T3/2015 | Other Database Import | Mukherjee,A.; Chawla-Sarkar,M. |
| EPI_ISL_217008 | West Bengal | 2015 | A/India/Kol-T4/2015 | Other Database Import | Mukherjee,A.; Chawla-Sarkar,M. |
| EPI_ISL_217009 | West Bengal | 2015 | A/India/Kol-T5/2015 | Other Database Import | Mukherjee,A.; Chawla-Sarkar,M. |
| EPI_ISL_217010 | West Bengal | 2015 | A/India/Kol-T15/2015 | Other Database Import | Mukherjee,A.; Chawla-Sarkar,M. |
| EPI_ISL_217011 | West Bengal | 2015 | A/India/Kol-T16/2015 | Other Database Import | Mukherjee,A.; Chawla-Sarkar,M. |
| EPI_ISL_217012 | West Bengal | 2015 | A/India/Kol-T8/2015 | Other Database Import | Mukherjee,A.; Chawla-Sarkar,M. |
| EPI_ISL_258875 | Delhi | 2015-01-09 | A/India/Jaipur152563/2015 | Other Database Import | Potdar,V.; Dakhave,M.; Hinge,D.; Bhosle,P.; Koul,P.; Dar,L.; Raj,K.; Chadha,M. |
| EPI_ISL_193011 | Maharashtra | 2015-01-09 | A/India/Kol-4501/2015 | Other Database Import | Mukherjee,A.; Chawla-Sarkar,M. |
| EPI_ISL_237085 | Kerala | 2015-01-14 | A/Kerala/RGCB145385/2015 | Other Database Import | Jones Palakkat,S.; Chirundodh,D.V.; Sujatha,A.S.; Fettle,A.; Jacob,J.; Pillai,M.R. |
| EPI_ISL_201017 | Madhya Pradesh | 2015-01-15 | A/Indore/3415/2015 | Other Database Import | Sahu,M.; Shukla,M.K.; Singh,N.; Barde,P.V. |
| EPI_ISL_258891 | Delhi | 2015-01-20 | A/India/Jaipur152573/2015 | Other Database Import | Potdar,V.; Dakhave,M.; Hinge,D.; Bhosle,P.; Koul,P.; Dar,L.; Raj,K.; Chadha,M. |
| EPI_ISL_258897 | Tamil Nadu | 2015-01-22 | A/Mysore/MCVRAF7638/2017 | Other Database Import | Jagadesh,A.; Arunkumar,G. |
| EPI_ISL_218432 | Maharashtra | 2015-01-23 | A/India/Pun151192/2015 | Other Database Import | Potdar,V.A.; Hinge,D.D.; Harpale,P.M. |
| EPI_ISL_258738 | Maharashtra | 2015-01-23 | A/India/Kol-S4587/2015 | Other Database Import | Mukherjee,A.; Chawla-Sarkar,M. |
| EPI_ISL_258896 | Tamil Nadu | 2015-01-23 | A/Nilgiris/MCVRAF7809/2017 | Other Database Import | Jagadesh,A.; Arunkumar,G. |
| EPI_ISL_258895 | Tamil Nadu | 2015-01-26 | A/Ernakulam/MCVRAF7821/2017 | Other Database Import | Jagadesh,A.; Arunkumar,G. |
| EPI_ISL_218433 | Maharashtra | 2015-01-27 | A/India/Pun151214/2015 | Other Database Import | Potdar,V.A.; Hinge,D.D.; Harpale,P.M. |
| EPI_ISL_218434 | Maharashtra | 2015-01-27 | A/India/Pun151268/2015 | Other Database Import | Potdar,V.A.; Hinge,D.D.; Harpale,P.M. |
| EPI_ISL_258737 | Maharashtra | 2015-01-27 | A/India/Kol-S4659/2015 | Other Database Import | Mukherjee,A.; Chawla-Sarkar,M. |
| EPI_ISL_258902 | Maharashtra | 2015-01-27 | A/India/P155351/2015 | Other Database Import | Potdar,V.; Dakhave,M.; Hinge,D.; Bhosle,P.; Koul,P.; Dar,L.; Raj,K.; Chadha,M. |
| EPI_ISL_201029 | Madhya Pradesh | 2015-01-28 | A/Bhopal/3500/2015 | Other Database Import | Sahu,M.; Shukla,M.K.; Singh,N.; Barde,P.V. |
| EPI_ISL_218431 | Maharashtra | 2015-01-28 | A/India/Pun151245/2015 | Other Database Import | Potdar,V.A.; Hinge,D.D.; Harpale,P.M. |
| EPI_ISL_218442 | Maharashtra | 2015-01-28 | A/India/Pun151247/2015 | Other Database Import | Potdar,V.A.; Hinge,D.D.; Harpale,P.M. |
| EPI_ISL_258743 | Maharashtra | 2015-01-28 | A/India/Kol-S4666/2015 | Other Database Import | Mukherjee,A.; Chawla-Sarkar,M. |
| EPI_ISL_258894 | Tamil Nadu | 2015-01-28 | A/Chikmagalur/MCVRAF7881/2017 | Other Database Import | Jagadesh,A.; Arunkumar,G. |
| EPI_ISL_258893 | Tamil Nadu | 2015-01-28 | A/Shimoga/MCVRAF9709/2017 | Other Database Import | Jagadesh,A.; Arunkumar,G. |
| EPI_ISL_258741 | Jammu & Kashmir | 2015-01-31 | A/India/Srinagar1510350/2015 | Other Database Import | Potdar,V.; Dakhave,M.; Hinge,D.; Bhosle,P.; Koul,P.; Dar,L.; Raj,K.; Chadha,M. |
| EPI_ISL_193014 | Maharashtra | 2015-01-31 | A/India/610/2015 | Centers for Disease Control and Prevention |  |
| EPI_ISL_218436 | Maharashtra | 2015-02-01 | A/India/Pun151399/2015 | Other Database Import | Potdar,V.A.; Hinge,D.D.; Harpale,P.M. |
| EPI_ISL_218435 | Maharashtra | 2015-02-02 | A/India/Pun151368/2015 | Other Database Import | Potdar,V.A.; Hinge,D.D.; Harpale,P.M. |
| EPI_ISL_258735 | Maharashtra | 2015-02-02 | A/India/P155355/2015 | Other Database Import | Potdar,V.; Dakhave,M.; Hinge,D.; Bhosle,P.; Koul,P.; Dar,L.; Raj,K.; Chadha,M. |
| EPI_ISL_218438 | Maharashtra | 2015-02-03 | A/India/Pun151508/2015 | Other Database Import | Potdar,V.A.; Hinge,D.D.; Harpale,P.M. |
| EPI_ISL_220979 | Jammu & Kashmir | 2015-02-03 | A/India/3275/2015 | Centers for Disease Control and Prevention | Garten,R. |
| EPI_ISL_258740 | Jammu & Kashmir | 2015-02-03 | A/India/Srinagar1513275/2015 | Other Database Import | Potdar,V.; Dakhave,M.; Hinge,D.; Bhosle,P.; Koul,P.; Dar,L.; Raj,K.; Chadha,M. |
| EPI_ISL_193013 | Maharashtra | 2015-02-03 | A/India/P155145/2015 | Other Database Import | Potdar,V.; Dakhave,M.; Hinge,D.; Bhosle,P.; Koul,P.; Dar,L.; Raj,K.; Chadha,M. |
| EPI_ISL_201018 | Madhya Pradesh | 2015-02-04 | A/Indore/3598/2015 | Other Database Import | Sahu,M.; Shukla,M.K.; Singh,N.; Barde,P.V. |
| EPI_ISL_220978 | Maharashtra | 2015-02-04 | A/India/7674/2015 | Centers for Disease Control and Prevention | Garten,R. |
| EPI_ISL_258874 | Maharashtra | 2015-02-04 | A/India/4880/2015 (H1N1) | National Centre for Disease Control (NCDC) |  |
| EPI_ISL_258890 | Delhi | 2015-02-05 | A/India/Jaipur152569/2015 | Other Database Import | Gurav,Y.K.; Chadha,M.S.; Tandale,B.V.; Potdar,V.A.; Pawar,S.D.; Shil,P.; Deoshatwar,A.R.; Aarthy,R.; Bhushan,A.; Dakhave,M.; Hinge,D.; Bhosle,P.; Koul,P.; Dar,L.; Raj,K. |
| EPI_ISL_237088 | Kerala | 2015-02-06 | A/Kerala/RGCB145751/2015 | Other Database Import | Jones Palakkat,S.; Chirundodh,D.V.; Sujatha,A.S.; Fettle,A.; Jacob,J.; Pillai,M.R. |
| EPI_ISL_258870 | Rajasthan | 2015-02-06 | A/India/Jaipur152563/2015 | Other Database Import | Potdar,V.; Dakhave,M.; Hinge,D.; Bhosle,P.; Koul,P.; Dar,L.; Raj,K.; Chadha,M. |
| EPI_ISL_258868 | Rajasthan | 2015-02-08 | A/India/Jaipur152573/2015 | Other Database Import | Potdar,V.; Dakhave,M.; Hinge,D.; Bhosle,P.; Koul,P.; Dar,L.; Raj,K.; Chadha,M. |
| EPI_ISL_258869 | Rajasthan | 2015-02-08 | A/India/Jaipur152569/2015 | Other Database Import | Gurav,Y.K.; Chadha,M.S.; Tandale,B.V.; Potdar,V.A.; Pawar,S.D.; Shil,P.; Deoshatwar,A.R.; Aarthy,R.; Bhushan,A.; Dakhave,M.; Hinge,D.; Bhosle,P.; Koul,P.; Dar,L.; Raj,K. |
| EPI_ISL_258739 | Jammu & Kashmir | 2015-02-09 | A/India/Srinagar1513276/2015 | Other Database Import | Potdar,V.; Dakhave,M.; Hinge,D.; Bhosle,P.; Koul,P.; Dar,L.; Raj,K.; Chadha,M. |
| EPI_ISL_279397 | Rajasthan | 2015-02-09 | A/Barmer/922/2015 | Other Database Import | Angel,B.; Angel,A.; Joshi,A.P.; Baharia,R.K.; Rathore,S.; Joshi,V. |
| EPI_ISL_279399 | Rajasthan | 2015-02-09 | A/Barmer/936/2015 | Other Database Import | Angel,B.; Angel,A.; Joshi,A.P.; Baharia,R.K.; Rathore,S.; Joshi,V. |
| EPI_ISL_237086 | Kerala | 2015-02-10 | A/Kerala/RGCB145808/2015 | Other Database Import | Jones Palakkat,S.; Chirundodh,D.V.; Sujatha,A.S.; Fettle,A.; Jacob,J.; Pillai,M.R. |
| EPI_ISL_258889 | Delhi | 2015-02-12 | A/India/Srinagar1513276/2015 | Other Database Import | Potdar,V.; Dakhave,M.; Hinge,D.; Bhosle,P.; Koul,P.; Dar,L.; Raj,K.; Chadha,M. |
| EPI_ISL_258863 | Maharashtra | 2015-02-12 | A/India/8900/2015 | Centers for Disease Control and Prevention |  |
| EPI_ISL_258888 | Delhi | 2015-02-13 | A/Barmer/922/2015 | Other Database Import | Angel,B.; Angel,A.; Joshi,A.P.; Baharia,R.K.; Rathore,S.; Joshi,V. |
| EPI_ISL_258862 | Maharashtra | 2015-02-13 | A/India/P155422/2015 | Other Database Import | Potdar,V.; Dakhave,M.; Hinge,D.; Bhosle,P.; Koul,P.; Dar,L.; Raj,K.; Chadha,M. |
| EPI_ISL_258861 | Maharashtra | 2015-02-14 | A/India/P158900/2015 | National Institute of Infectious Diseases (NIID) | Takashita,Emi; Fujisaki,Seiichiro; Shirakura,Masayuki; Watanabe,Shinji; Odagiri,Takato |
| EPI_ISL_195927 | Delhi | 2015-02-15 | A/India/4101/2015 (H1N1) | National Centre for Disease Control (NCDC) | Datt,T. |
| EPI_ISL_195928 | Delhi | 2015-02-15 | A/India/4302/2015 (H1N1) | National Centre for Disease Control (NCDC) | Datt,T. |
| EPI_ISL_258887 | Delhi | 2015-02-16 | A/Barmer/936/2015 | Other Database Import | Angel,B.; Angel,A.; Joshi,A.P.; Baharia,R.K.; Rathore,S.; Joshi,V. |
| EPI_ISL_258886 | Delhi | 2015-02-16 | A/Kerala/RGCB145808/2015 | Other Database Import | Jones Palakkat,S.; Chirundodh,D.V.; Sujatha,A.S.; Fettle,A.; Jacob,J.; Pillai,M.R. |
| EPI_ISL_258885 | Delhi | 2015-02-16 | A/India/D1510031/2015 | Other Database Import | Potdar,V.; Dakhave,M.; Hinge,D.; Bhosle,P.; Koul,P.; Dar,L.; Raj,K.; Chadha,M. |
| EPI_ISL_258860 | Maharashtra | 2015-02-16 | A/India/P157674/2015 | National Institute of Infectious Diseases (NIID) | Takashita,Emi; Fujisaki,Seiichiro; Shirakura,Masayuki; Watanabe,Shinji; Odagiri,Takato |
| EPI_ISL_258859 | Maharashtra | 2015-02-16 | A/Assam/SW-84/2015 | Other Database Import | Biswas,D.; Sarmah,K.; Dutta,M.; Buragohain,M.; Yadav,K.; Borkakoty,B. |
| EPI_ISL_201019 | Madhya Pradesh | 2015-02-17 | A/Dewas/4497/2015 | Other Database Import | Sahu,M.; Shukla,M.K.; Singh,N.; Barde,P.V. |
| EPI_ISL_258731 | Jammu & Kashmir | 2015-02-18 | A/India/Srinagar1513278/2015 | Other Database Import | Potdar,V.; Dakhave,M.; Hinge,D.; Bhosle,P.; Koul,P.; Dar,L.; Raj,K.; Chadha,M. |
| EPI_ISL_258732 | Jammu & Kashmir | 2015-02-18 | A/India/Srinagar1513277/2015 | Other Database Import | Potdar,V.; Dakhave,M.; Hinge,D.; Bhosle,P.; Koul,P.; Dar,L.; Raj,K.; Chadha,M. |
| EPI_ISL_195929 | Haryana | 2015-02-19 | A/India/4880/2015 (H1N1) | National Centre for Disease Control (NCDC) | Datt,T. |
| EPI_ISL_258884 | Delhi | 2015-02-19 | A/India/P151963/2015 | Other Database Import | Potdar,V.; Dakhave,M.; Hinge,D.; Bhosle,P.; Koul,P.; Dar,L.; Raj,K.; Chadha,M. |
| EPI_ISL_218437 | Maharashtra | 2015-02-20 | A/India/Pun153225/2015 | Other Database Import | Potdar,V.A.; Hinge,D.D.; Harpale,P.M. |
| EPI_ISL_258883 | Delhi | 2015-02-20 | A/India/D1510032/2015 | Other Database Import | Potdar,V.; Dakhave,M.; Hinge,D.; Bhosle,P.; Koul,P.; Dar,L.; Raj,K.; Chadha,M. |
| EPI_ISL_258901 | Maharashtra | 2015-02-20 | A/India/4051/2015 | Centers for Disease Control and Prevention |  |
| EPI_ISL_258873 | Maharashtra | 2015-02-23 | A/India/C1510064/2015 | Other Database Import | Potdar,V.; Dakhave,M.; Hinge,D.; Bhosle,P.; Koul,P.; Dar,L.; Raj,K.; Chadha,M. |
| EPI_ISL_193006 | Maharashtra | 2015-02-23 | A/India/3175/2015 | Centers for Disease Control and Prevention |  |
| EPI_ISL_258882 | Delhi | 2015-02-24 | A/India/P152122/2015 | Other Database Import | Potdar,V.; Dakhave,M.; Hinge,D.; Bhosle,P.; Koul,P.; Dar,L.; Raj,K.; Chadha,M. |
| EPI_ISL_201026 | Madhya Pradesh | 2015-02-25 | A/Ujjain/5448/2015 | Other Database Import | Sahu,M.; Shukla,M.K.; Singh,N.; Barde,P.V. |
| EPI_ISL_193007 | Maharashtra | 2015-02-25 | A/India/4816/2015 | Centers for Disease Control and Prevention |  |
| EPI_ISL_258872 | Maharashtra | 2015-02-25 | A/India/Kol-4992/2015 | Other Database Import | Mukherjee,A.; Chawla-Sarkar,M. |
| EPI_ISL_195930 | Delhi | 2015-02-27 | A/India/6691/2015 (H1N1) | National Centre for Disease Control (NCDC) | Datt,T. |
| EPI_ISL_218439 | Maharashtra | 2015-02-27 | A/India/Pun153389/2015 | Other Database Import | Potdar,V.A.; Hinge,D.D.; Harpale,P.M. |
| EPI_ISL_218440 | Maharashtra | 2015-02-27 | A/India/Pun153388/2015 | Other Database Import | Potdar,V.A.; Hinge,D.D.; Harpale,P.M. |
| EPI_ISL_258867 | Rajasthan | 2015-02-27 | A/India/Jaipur153442/2015 | Other Database Import | Potdar,V.; Dakhave,M.; Hinge,D.; Bhosle,P.; Koul,P.; Dar,L.; Raj,K.; Chadha,M. |
| EPI_ISL_258881 | Delhi | 2015-02-27 | A/India/P152151/2015 | Other Database Import | Gurav,Y.K.; Chadha,M.S.; Tandale,B.V.; Potdar,V.A.; Pawar,S.D.; Shil,P.; Deoshatwar,A.R.; Aarthy,R.; Bhushan,A.; Dakhave,M.; Hinge,D.; Bhosle,P.; Koul,P.; Dar,L.; Raj,K. |
| EPI_ISL_258900 | Maharashtra | 2015-02-27 | A/India/Kol-5018/2015 | Other Database Import | Mukherjee,A.; Chawla-Sarkar,M. |
| EPI_ISL_258899 | Maharashtra | 2015-02-27 | A/India/Kol-5025/2015 | Other Database Import | Mukherjee,A.; Chawla-Sarkar,M. |
| EPI_ISL_201027 | Madhya Pradesh | 2015-03-01 | A/Barwani/6024/2015 | Other Database Import | Sahu,M.; Shukla,M.K.; Singh,N.; Barde,P.V. |
| EPI_ISL_258730 | Jammu & Kashmir | 2015-03-02 | A/India/Srinagar1513274/2015 | Other Database Import | Potdar,V.; Dakhave,M.; Hinge,D.; Bhosle,P.; Koul,P.; Dar,L.; Raj,K.; Chadha,M. |
| EPI_ISL_258871 | Jammu & Kashmir | 2015-03-02 | A/India/Srinagar1510349/2015 | Other Database Import | Potdar,V.; Dakhave,M.; Hinge,D.; Bhosle,P.; Koul,P.; Dar,L.; Raj,K.; Chadha,M. |
| EPI_ISL_195931 | Uttarakhand | 2015-03-03 | A/India/8259/2015 (H1N1) | National Centre for Disease Control (NCDC) | Datt,T. |
| EPI_ISL_258880 | Delhi | 2015-03-03 | A/India/4101/2015 (H1N1) | National Centre for Disease Control (NCDC) |  |
| EPI_ISL_258879 | Delhi | 2015-03-03 | A/India/4302/2015 (H1N1) | National Centre for Disease Control (NCDC) |  |
| EPI_ISL_258858 | Maharashtra | 2015-03-03 | A/India/5714/2015 | Centers for Disease Control and Prevention |  |
| EPI_ISL_195932 | Haryana | 2015-03-04 | A/India/8448/2015 (2015) | National Centre for Disease Control (NCDC) | Datt,T. |
| EPI_ISL_218441 | Maharashtra | 2015-03-04 | A/India/Pun153793/2015 | Other Database Import | Potdar,V.A.; Hinge,D.D.; Harpale,P.M. |
| EPI_ISL_258729 | Jammu & Kashmir | 2015-03-04 | A/India/Srinagar1510348/2015 | Other Database Import | Potdar,V.; Dakhave,M.; Hinge,D.; Bhosle,P.; Koul,P.; Dar,L.; Raj,K.; Chadha,M. |
| EPI_ISL_193012 | Maharashtra | 2015-03-04 | A/India/P155134/2015 | Other Database Import | Potdar,V.; Dakhave,M.; Hinge,D.; Bhosle,P.; Koul,P.; Dar,L.; Raj,K.; Chadha,M. |
| EPI_ISL_258898 | Maharashtra | 2015-03-04 | A/India/Kol-5065/2015 | Other Database Import | Mukherjee,A.; Chawla-Sarkar,M. |
| EPI_ISL_266412 | Madhya Pradesh | 2015-03-05 | A/India/DRDE_GWL672/2015 | Other Database Import | Dash,P.K.; Pattabiraman,C.; Tandel,K.; Sharma,S.; Siddappa,S.; Kumar,J.; Gowda,M.; Krishna,S.; Parida,M.M. |
| EPI_ISL_258866 | Maharashtra | 2015-03-05 | A/India/1674/2016 | Centers for Disease Control and Prevention |  |
| EPI_ISL_234592 | West Bengal | 2015-03-06 | A/India/Kol-3828/2015 | Other Database Import | Mukherjee,A.; Chawla-Sarkar,M. |
| EPI_ISL_258728 | Jammu & Kashmir | 2015-03-06 | A/India/Srinagar1513279/2015 | Other Database Import | Potdar,V.; Dakhave,M.; Hinge,D.; Bhosle,P.; Koul,P.; Dar,L.; Raj,K.; Chadha,M. |
| EPI_ISL_220491 | Madhya Pradesh | 2015-03-07 | A/India/DRDE_GWL703/2015 | Other Database Import | Parida,M.; Dash,P.K.; Kumar,J.S.; Joshi,G.; Tandel,K.; Sharma,S.; Srivastava,A.; Agarwal,A.; Saha,A.; Saraswat,S.; Karothia,D.; Malviya,V. |
| EPI_ISL_220486 | Madhya Pradesh | 2015-03-08 | A/India/DRDE_GWL719/2015 | Other Database Import | Parida,M.; Dash,P.K.; Kumar,J.S.; Joshi,G.; Tandel,K.; Sharma,S.; Srivastava,A.; Agarwal,A.; Saha,A.; Saraswat,S.; Karothia,D.; Malviya,V. |
| EPI_ISL_220489 | Madhya Pradesh | 2015-03-08 | A/India/DRDE_GWL721/2015 | Other Database Import | Parida,M.; Dash,P.K.; Kumar,J.S.; Joshi,G.; Tandel,K.; Sharma,S.; Srivastava,A.; Agarwal,A.; Saha,A.; Saraswat,S.; Karothia,D.; Malviya,V. |
| EPI_ISL_234593 | West Bengal | 2015-03-08 | A/India/Kol-3846/2015 | Other Database Import | Mukherjee,A.; Chawla-Sarkar,M. |
| EPI_ISL_234594 | West Bengal | 2015-03-09 | A/India/Kol-3959/2015 | Other Database Import | Mukherjee,A.; Chawla-Sarkar,M. |
| EPI_ISL_266416 | Madhya Pradesh | 2015-03-09 | A/India/DRDE_GWL812/2015 | Other Database Import | Dash,P.K.; Pattabiraman,C.; Tandel,K.; Sharma,S.; Siddappa,S.; Kumar,J.; Gowda,M.; Krishna,S.; Parida,M.M. |
| EPI_ISL_258878 | Delhi | 2015-03-10 | A/India/D1510033/2015 | Other Database Import | Potdar,V.; Dakhave,M.; Hinge,D.; Bhosle,P.; Koul,P.; Dar,L.; Raj,K.; Chadha,M. |
| EPI_ISL_220490 | Madhya Pradesh | 2015-03-11 | A/India/DRDE_GWL812/2015 | Other Database Import | Parida,M.; Dash,P.K.; Kumar,J.S.; Joshi,G.; Tandel,K.; Sharma,S.; Srivastava,A.; Agarwal,A.; Saha,A.; Saraswat,S.; Karothia,D.; Malviya,V. |
| EPI_ISL_234595 | West Bengal | 2015-03-11 | A/India/Kol-4040/2015 | Other Database Import | Mukherjee,A.; Chawla-Sarkar,M. |
| EPI_ISL_234596 | West Bengal | 2015-03-13 | A/India/Kol-4122/2015 | Other Database Import | Mukherjee,A.; Chawla-Sarkar,M. |
| EPI_ISL_193008 | Maharashtra | 2015-03-13 | A/India/1761/2016 | Centers for Disease Control and Prevention |  |
| EPI_ISL_258857 | Maharashtra | 2015-03-13 | A/India/1819/2016 | Centers for Disease Control and Prevention |  |
| EPI_ISL_234587 | West Bengal | 2015-03-14 | A/India/Kol-S4163/2015 | Other Database Import | Mukherjee,A.; Chawla-Sarkar,M. |
| EPI_ISL_266422 | Madhya Pradesh | 2015-03-14 | A/India/DRDE_GWL897/2015 | Other Database Import | Dash,P.K.; Pattabiraman,C.; Tandel,K.; Sharma,S.; Siddappa,S.; Kumar,J.; Gowda,M.; Krishna,S.; Parida,M.M. |
| EPI_ISL_220487 | Madhya Pradesh | 2015-03-15 | A/India/DRDE_GWL897/2015 | Other Database Import | Parida,M.; Dash,P.K.; Kumar,J.S.; Joshi,G.; Tandel,K.; Sharma,S.; Srivastava,A.; Agarwal,A.; Saha,A.; Saraswat,S.; Karothia,D.; Malviya,V. |
| EPI_ISL_201028 | Madhya Pradesh | 2015-03-17 | A/Betul/6515/2015 | Other Database Import | Sahu,M.; Shukla,M.K.; Singh,N.; Barde,P.V. |
| EPI_ISL_266411 | Madhya Pradesh | 2015-03-17 | A/India/DRDE_GWL84/2015 | Other Database Import | Dash,P.K.; Pattabiraman,C.; Tandel,K.; Sharma,S.; Siddappa,S.; Kumar,J.; Gowda,M.; Krishna,S.; Parida,M.M. |
| EPI_ISL_258877 | Delhi | 2015-03-17 | A/India/D1510035/2015 | Other Database Import | Potdar,V.; Dakhave,M.; Hinge,D.; Bhosle,P.; Koul,P.; Dar,L.; Raj,K.; Chadha,M. |
| EPI_ISL_258876 | Delhi | 2015-03-17 | A/India/D1510036/2015 | Other Database Import | Potdar,V.; Dakhave,M.; Hinge,D.; Bhosle,P.; Koul,P.; Dar,L.; Raj,K.; Chadha,M. |
| EPI_ISL_258748 | Maharashtra | 2015-03-17 | A/India/3725/2016 | Centers for Disease Control and Prevention |  |
| EPI_ISL_220488 | Madhya Pradesh | 2015-03-18 | A/India/DRDE_GWL989/2015 | Other Database Import | Parida,M.; Dash,P.K.; Kumar,J.S.; Joshi,G.; Tandel,K.; Sharma,S.; Srivastava,A.; Agarwal,A.; Saha,A.; Saraswat,S.; Karothia,D.; Malviya,V. |
| EPI_ISL_266423 | Madhya Pradesh | 2015-03-18 | A/India/DRDE_GWL989/2015 | Other Database Import | Dash,P.K.; Pattabiraman,C.; Tandel,K.; Sharma,S.; Siddappa,S.; Kumar,J.; Gowda,M.; Krishna,S.; Parida,M.M. |
| EPI_ISL_258749 | Maharashtra | 2015-03-18 | A/India/1819/2016 | Centers for Disease Control and Prevention |  |
| EPI_ISL_234588 | West Bengal | 2015-03-21 | A/India/Kol-S4481/2015 | Other Database Import | Mukherjee,A.; Chawla-Sarkar,M. |
| EPI_ISL_234597 | West Bengal | 2015-03-21 | A/India/Kol-4501/2015 | Other Database Import | Mukherjee,A.; Chawla-Sarkar,M. |
| EPI_ISL_258747 | Maharashtra | 2015-03-21 | A/Assam/RMRC_226/2016 | Other Database Import | Borkakoty,B.; Jakharia,A.; Sarmah,K.; Hazarika,R.; Biswas,D.; Mahanta,J. |
| EPI_ISL_258746 | Maharashtra | 2015-03-22 | A/India/7506/2016 | Centers for Disease Control and Prevention |  |
| EPI_ISL_220980 | Jammu & Kashmir | 2015-03-23 | A/India/610/2015 | Centers for Disease Control and Prevention | Garten,R. |
| EPI_ISL_234589 | West Bengal | 2015-03-23 | A/India/Kol-S4587/2015 | Other Database Import | Mukherjee,A.; Chawla-Sarkar,M. |
| EPI_ISL_234590 | West Bengal | 2015-03-25 | A/India/Kol-S4659/2015 | Other Database Import | Mukherjee,A.; Chawla-Sarkar,M. |
| EPI_ISL_234591 | West Bengal | 2015-03-25 | A/India/Kol-S4666/2015 | Other Database Import | Mukherjee,A.; Chawla-Sarkar,M. |
| EPI_ISL_258745 | Maharashtra | 2015-03-28 | A/India/4183/2016 | Centers for Disease Control and Prevention |  |
| EPI_ISL_258744 | Maharashtra | 2015-03-28 | A/India/7512/2016 | Centers for Disease Control and Prevention |  |
| EPI_ISL_220977 | West Bengal | 2015-03-30 | A/India/8900/2015 | Centers for Disease Control and Prevention | Garten,R. |
| EPI_ISL_258742 | Maharashtra | 2015-03-30 | A/Assam/RMRC_449/2016 | Other Database Import | Borkakoty,B.; Jakharia,A.; Sarmah,K.; Hazarika,R.; Biswas,D.; Mahanta,J. |
| EPI_ISL_193010 | Maharashtra | 2015-03-30 | A/Assam/RMRC_527/2016 | Other Database Import | Borkakoty,B.; Jakharia,A.; Sarmah,K.; Hazarika,R.; Biswas,D.; Mahanta,J. |
| EPI_ISL_193009 | Maharashtra | 2015-04-02 | A/Assam/RMRC_494/2016 | Other Database Import | Borkakoty,B.; Jakharia,A.; Sarmah,K.; Hazarika,R.; Biswas,D.; Mahanta,J. |
| EPI_ISL_206031 | Assam | 2015-04-18 | A/Assam/SW-84/2015 | Other Database Import | Biswas,D.; Sarmah,K.; Dutta,M.; Buragohain,M.; Yadav,K.; Borkakoty,B. |
| EPI_ISL_258892 | Tamil Nadu | 2015-05-04 | A/Calicut/MCVRAF9834/2017 | Other Database Import | Jagadesh,A.; Arunkumar,G. |
| EPI_ISL_220974 | Maharashtra | 2015-08-15 | A/India/3175/2015 | Centers for Disease Control and Prevention | Garten,R. |
| EPI_ISL_224289 | Maharashtra | 2015-09-01 | A/India/4051/2015 | Centers for Disease Control and Prevention | Garten,R. |
| EPI_ISL_220975 | Maharashtra | 2015-09-07 | A/India/4816/2015 | Centers for Disease Control and Prevention | Garten,R. |
| EPI_ISL_234598 | West Bengal | 2015-09-09 | A/India/Kol-4992/2015 | Other Database Import | Mukherjee,A.; Chawla-Sarkar,M. |
| EPI_ISL_234599 | West Bengal | 2015-09-16 | A/India/Kol-5018/2015 | Other Database Import | Mukherjee,A.; Chawla-Sarkar,M. |
| EPI_ISL_234600 | West Bengal | 2015-09-17 | A/India/Kol-5025/2015 | Other Database Import | Mukherjee,A.; Chawla-Sarkar,M. |
| EPI_ISL_220976 | Maharashtra | 2015-09-24 | A/India/5714/2015 | Centers for Disease Control and Prevention | Garten,R. |
| EPI_ISL_234601 | West Bengal | 2015-10-07 | A/India/Kol-5065/2015 | Other Database Import | Mukherjee,A.; Chawla-Sarkar,M. |
| EPI_ISL_234639 | Maharashtra | 2016-02-14 | A/India/1674/2016 | Centers for Disease Control and Prevention | Garten,R. |
| EPI_ISL_234640 | Maharashtra | 2016-02-15 | A/India/1761/2016 | Centers for Disease Control and Prevention | Garten,R. |
| EPI_ISL_234641 | Maharashtra | 2016-02-18 | A/India/1819/2016 | Centers for Disease Control and Prevention | Garten,R. |
| EPI_ISL_239246 | Maharashtra | 2016-02-18 | A/India/1819/2016 | Centers for Disease Control and Prevention | Garten,R. |
| EPI_ISL_233514 | Maharashtra | 2016-03-16 | A/India/3725/2016 | Centers for Disease Control and Prevention | Garten,R. |
| EPI_ISL_279412 | Assam | 2016-03-16 | A/Assam/RMRC_226/2016 | Other Database Import | Borkakoty,B.; Jakharia,A.; Sarmah,K.; Hazarika,R.; Biswas,D.; Mahanta,J. |
| EPI_ISL_233516 | Maharashtra | 2016-03-18 | A/India/7506/2016 | Centers for Disease Control and Prevention | Garten,R. |
| EPI_ISL_233515 | Maharashtra | 2016-03-24 | A/India/4183/2016 | Centers for Disease Control and Prevention | Garten,R. |
| EPI_ISL_233517 | Maharashtra | 2016-03-24 | A/India/7512/2016 | Centers for Disease Control and Prevention | Garten,R. |
| EPI_ISL_279416 | Assam | 2016-06-06 | A/Assam/RMRC_449/2016 | Other Database Import | Borkakoty,B.; Jakharia,A.; Sarmah,K.; Hazarika,R.; Biswas,D.; Mahanta,J. |
| EPI_ISL_279406 | Assam | 2016-06-13 | A/Assam/RMRC_494/2016 | Other Database Import | Borkakoty,B.; Jakharia,A.; Sarmah,K.; Hazarika,R.; Biswas,D.; Mahanta,J. |
| EPI_ISL_279407 | Assam | 2016-06-21 | A/Assam/RMRC_527/2016 | Other Database Import | Borkakoty,B.; Jakharia,A.; Sarmah,K.; Hazarika,R.; Biswas,D.; Mahanta,J. |
| EPI_ISL_279415 | Assam | 2016-06-21 | A/Assam/RMRC_534/2016 | Other Database Import | Borkakoty,B.; Jakharia,A.; Sarmah,K.; Hazarika,R.; Biswas,D.; Mahanta,J. |
| EPI_ISL_279408 | Assam | 2016-06-24 | A/Assam/RMRC_598/2016 | Other Database Import | Borkakoty,B.; Jakharia,A.; Sarmah,K.; Hazarika,R.; Biswas,D.; Mahanta,J. |
| EPI_ISL_279409 | Assam | 2016-06-27 | A/Assam/RMRC_605/2016 | Other Database Import | Borkakoty,B.; Jakharia,A.; Sarmah,K.; Hazarika,R.; Biswas,D.; Mahanta,J. |
| EPI_ISL_279410 | Assam | 2016-06-27 | A/Assam/RMRC_609/2016 | Other Database Import | Borkakoty,B.; Jakharia,A.; Sarmah,K.; Hazarika,R.; Biswas,D.; Mahanta,J. |
| EPI_ISL_279413 | Assam | 2016-07-06 | A/Assam/RMRC_693/2016 | Other Database Import | Borkakoty,B.; Jakharia,A.; Sarmah,K.; Hazarika,R.; Biswas,D.; Mahanta,J. |
| EPI_ISL_279411 | Assam | 2016-07-13 | A/Assam/RMRC_709/2016 | Other Database Import | Borkakoty,B.; Jakharia,A.; Sarmah,K.; Hazarika,R.; Biswas,D.; Mahanta,J. |
| EPI_ISL_279414 | Assam | 2016-07-13 | A/Assam/RMRC_711/2016 | Other Database Import | Borkakoty,B.; Jakharia,A.; Sarmah,K.; Hazarika,R.; Biswas,D.; Mahanta,J. |
| EPI_ISL_290177 | Karnataka | 2017-01-27 | A/Kolar/MCVRAF3154/2017 | Other Database Import | Jagadesh,A.; Arunkumar,G. |
| EPI_ISL_290173 | Karnataka | 2017-03-25 | A/Mysore/MCVRAF7729/2017 | Other Database Import | Jagadesh,A.; Arunkumar,G. |
| EPI_ISL_290174 | Karnataka | 2017-03-25 | A/Mysore/MCVRAF7736/2017 | Other Database Import | Jagadesh,A.; Arunkumar,G. |
| EPI_ISL_290178 | Karnataka | 2017-03-25 | A/Mysore/MCVRAF7638/2017 | Other Database Import | Jagadesh,A.; Arunkumar,G. |
| EPI_ISL_290179 | Tamil Nadu | 2017-03-28 | A/Assam/RMRC_534/2016 | Other Database Import | Borkakoty,B.; Jakharia,A.; Sarmah,K.; Hazarika,R.; Biswas,D.; Mahanta,J. |
| EPI_ISL_290175 | Kerala | 2017-03-29 | A/Ernakulam/MCVRAF7821/2017 | Other Database Import | Jagadesh,A.; Arunkumar,G. |
| EPI_ISL_290176 | Karnataka | 2017-03-30 | A/Chikmagalur/MCVRAF7881/2017 | Other Database Import | Jagadesh,A.; Arunkumar,G. |
| EPI_ISL_290180 | Karnataka | 2017-04-24 | A/Shimoga/MCVRAF9709/2017 | Other Database Import | Jagadesh,A.; Arunkumar,G. |
| EPI_ISL_290181 | Kerala | 2017-04-24 | A/Calicut/MCVRAF9834/2017 | Other Database Import | Jagadesh,A.; Arunkumar,G. |
