# Supplementary material for: Phylodynamics of Influenza A/H1N1pdm09 in India Reveals Circulation Patterns and Increased Selection for Clade 6b Residues and Other High Mortality Mutants
Source: Viruses. 2019 Aug 27;11(9):791. doi: 10.3390/v11090791 (PMC6783925; doi:10.3390/v11090791)
Supplement: Supplementary file 1 [file viruses-11-00791-s001.zip › Supplementary Material.docx]

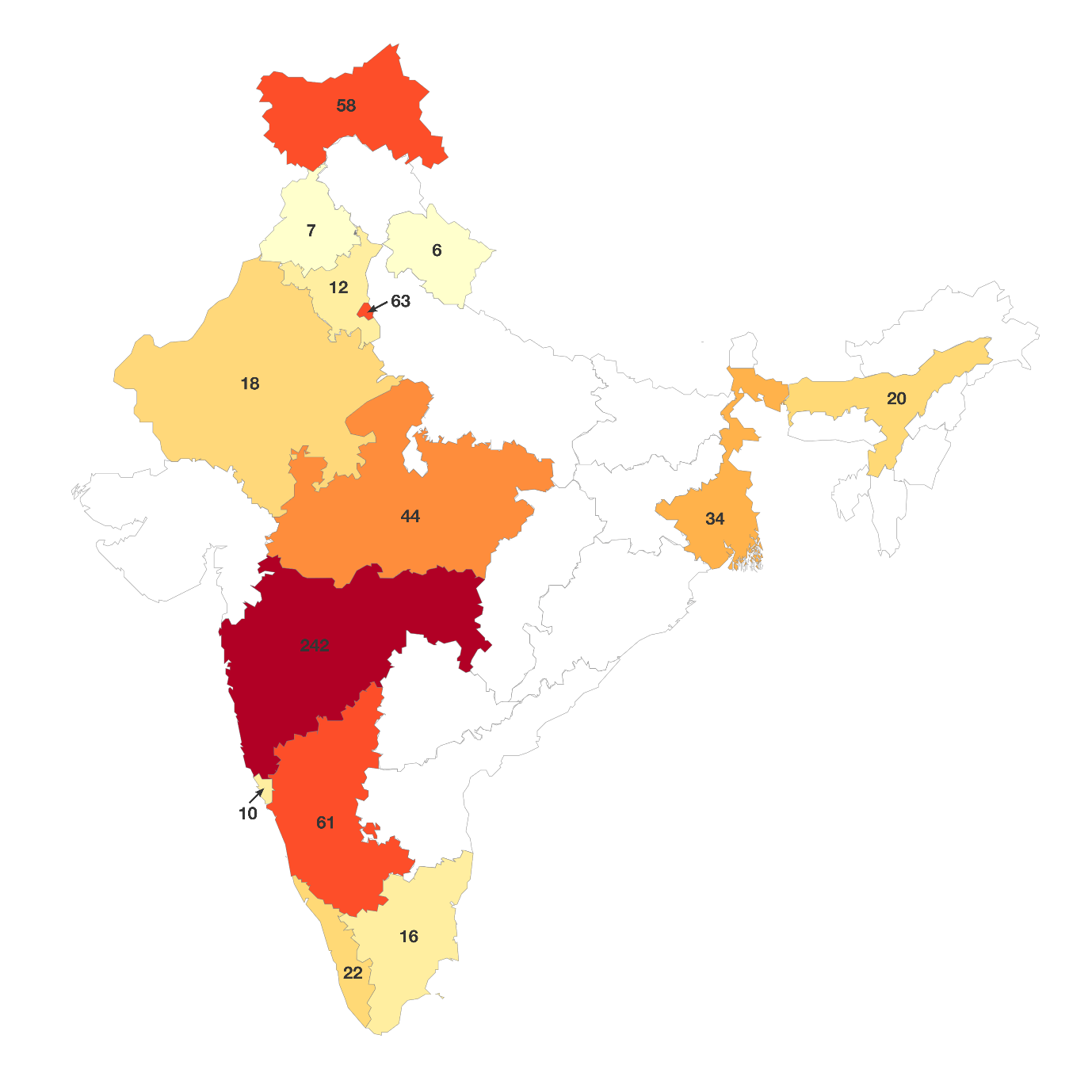


**Figure S1:** Spatial distribution of 613 taxa included in the final dataset sampled in India between 2009 and 2017 by S/UT.

Table S1. Global dataset of HA sequences by year and region for comparative analysis.

|  | **Year** | | | | | | | |  |  |  | |
| --- | --- | --- | --- | --- | --- | --- | --- | --- | --- | --- | --- | --- |
| **S/UT** | **2009** | **2010** | **2011** | **2012** | **2013** | **2014** | **2015** | **2016** | **2017** |  | **Total** |  |
| Africa | 99 | 89 | 60 | 47 | 79 | 40 | 216 | 287 | 348 |  | 1,265 | 5.96 |
| China | 662 | 259 | 100 | 18 | 78 | 60 | 148 | 162 | 208 |  | 1,695 | 7.99 |
| Europe | 1,456 | 200 | 250 | 128 | 354 | 413 | 617 | 1,291 | 438 |  | 5,147 | 24.27 |
| Japan/Korea | 550 | 76 | 23 | 15 | 104 | 193 | 87 | 268 | 178 |  | 1,494 | 7.04 |
| Middle East /  Western Asia | 73 | 28 | 23 | 37 | 52 | 53 | 255 | 235 | 305 |  | 1,061 | 5.00 |
| North America | 1,895 | 275 | 204 | 245 | 351 | 248 | 320 | 1,457 | 657 |  | 5,652 | 26.65 |
| Northern Asia | 150 | 14 | 65 | 5 | 95 | 66 | 162 | 402 | 17 |  | 976 | 4.60 |
| Oceania | 173 | 14 | 12 | 24 | 119 | 149 | 38 | 152 | 150 |  | 831 | 3.92 |
| South America | 195 | 59 | 57 | 120 | 143 | 79 | 110 | 553 | 47 |  | 1,363 | 6.43 |
| Southern Asia /  South East Asia | 263 | 42 | 22 | 47 | 131 | 97 | 383 | 366 | 374 |  | 1,725 | 8.13 |
| Year Total | 5,516 | 1,056 | 816 | 686 | 1,506 | 1,398 | 2,336 | 5,173 | 2,722 |  | 21,209 | 100.00 |

Table S2: Substitution model and clock prior testing results using path sampling (PS) and stepping-stone sampling (SSS) of log marginal likelihoods.

| **Sites Model** |  | **HKY** | | | |  | **GTR** | | | | |
| --- | --- | --- | --- | --- | --- | --- | --- | --- | --- | --- | --- |
| **Clock Model** |  | **Relaxed** | | **Strict** | |  |  | **Relaxed** | | **Strict** | |
| **Statistic** |  | **PS** | **SSS** | **PS** | **SSS** |  |  | **PS** | **SSS** | **PS** | **SSS** |
| **Run 1** |  | -18,850 | -18,893 | -18,977 | -19,006 |  |  | -18,796 | -18,836 | -18,897 | -18,930 |
| **Run 2** |  | -18,857 | -18,894 | -18,935 | -18,967 |  |  | -18,803 | -18,840 | -18,865 | -18,897 |
| **Average** |  | -18,854 | -18,894 | -18,956 | -18,986 |  |  | -18,800 | -18,838 | -18,881 | -18,914 |

**
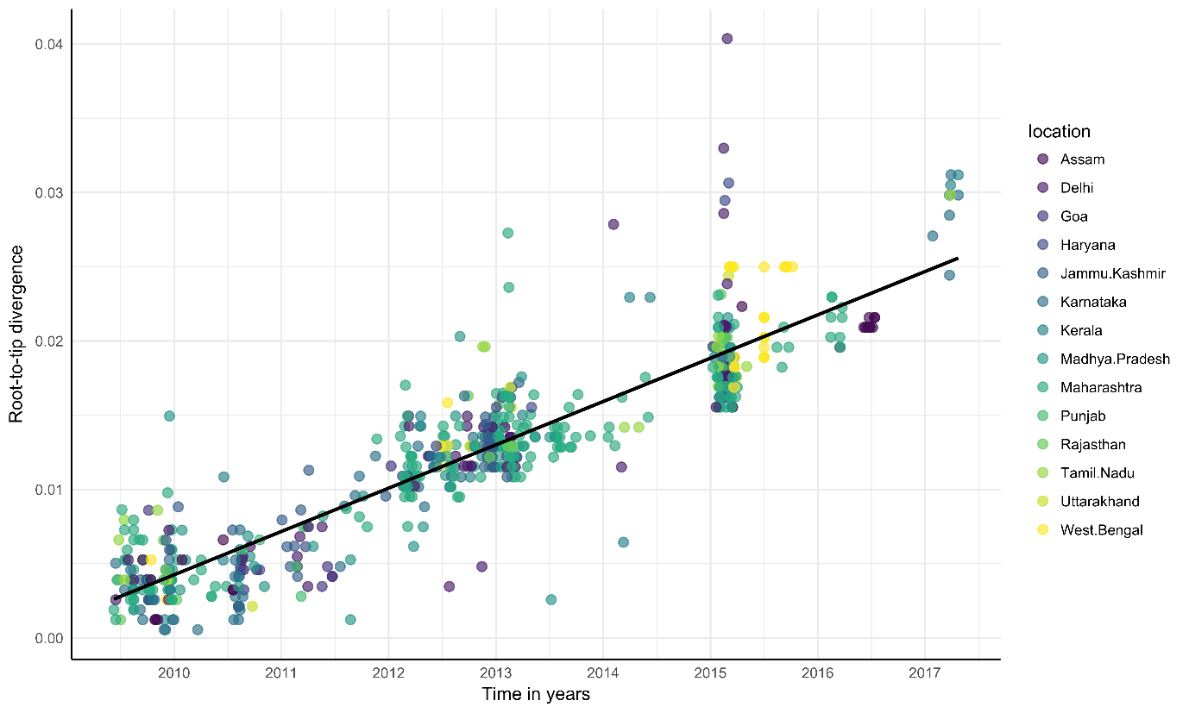
**

**Figure S2:** Root-to-tip regression over time of 613 A/H1N1pdm09 isolates sampled from India between 2009 and 2017. Demonstrating temporal signal suitable for time-scaled analysis and suggestive of relaxed molecular clock model assumptions.

Table S3. Interpretation of computed Bayes Factor values

| **Bayes Factor (BF)** | **Interpretation of Evidence** |
| --- | --- |
| >100 | Decisive |
| 30-100 | Very strong |
| 10-30 | Strong |
| 3-10 | Substantial |
| Adapted from Liang F (2013) Table 1 and Jeffreys H (1961) | |

Table S4. Subsample of HA sequences by year and S/UT included for replicate analysis

|  | **Year** | | | | | | | |  |  |  | |
| --- | --- | --- | --- | --- | --- | --- | --- | --- | --- | --- | --- | --- |
| **S/UT (Population 10^6^)** | **2009** | **2010** | **2011** | **2012** | **2013** | **2014** | **2015** | **2016** | **2017** |  | **Total** | **%** |
| Assam (31.2) | 4 | 4 |  |  |  |  | 1 | 5 |  |  | 14 | 5.4 |
| Delhi (16.8) | 5 | 4 | 5 | 5 | 5 | 2 | 5 |  |  |  | 31 | 12.0 |
| Goa (1.5) | 1 | 3 | 3 | 3 |  |  |  |  |  |  | 10 | 3.9 |
| Haryana (25.4) | 1 | 2 | 1 | 3 | 3 |  | 2 |  |  |  | 12 | 4.6 |
| Jammu & Kashmir (12.5) | 1 | 2 | 5 | 5 | 5 |  | 5 |  |  |  | 23 | 8.9 |
| Karnataka (61.1) | 5 | 5 | 5 | 5 |  |  |  |  | 5 |  | 25 | 9.7 |
| Kerala (33.4) | 1 | 2 |  | 5 | 2 | 3 | 3 |  | 2 |  | 18 | 6.9 |
| Madhya Pradesh (72.6) | 4 | 5 | 4 | 5 | 5 |  | 5 |  |  |  | 28 | 10.8 |
| Maharashtra (112.4) | 5 | 5 | 5 | 5 | 5 | 5 | 5 | 5 |  |  | 40 | 15.4 |
| Punjab (27.7) | 1 | 2 | 4 |  |  |  |  |  |  |  | 7 | 2.7 |
| Rajasthan (68.6) | 3 | 1 |  | 3 | 5 |  | 5 |  |  |  | 17 | 6.6 |
| Tamil Nadu (72.15) | 5 |  |  | 2 |  | 2 | 5 |  | 1 |  | 15 | 5.8 |
| Uttarakhand (10.1) | 1 | 1 |  | 1 | 2 |  | 1 |  |  |  | 6 | 2.3 |
| West Bengal (91.3) | 3 |  |  | 5 |  |  | 5 |  |  |  | 13 | 5.0 |
| Year Total | 40 | 36 | 32 | 47 | 32 | 12 | 42 | 10 | 8 |  | 259 | 100.0 |

Table S5: d_N_/d_S_ rate ratios and 95% BCI of all positively selected HA sites among Indian (N=613) and International (S1 & S2) taxa of H1N1pdm09 detected using renaissance counting (REN).

| **Site (H3#)** | **India Taxa (N = 613)** | | |  | **International Taxa (S1)** | | |  | **International Taxa (S2)** | | |
| --- | --- | --- | --- | --- | --- | --- | --- | --- | --- | --- | --- |
|  | **d_N_/d_S_** | **95% BCI** | **Sig** |  | **d_N_/d_S_** | **95% BCI** | **Sig** |  | **d_N_/d_S_** | **95% BCI** | **Sig** |
| 3 (-) | 0.60 | (0.25 - 0.98) |  |  | 0.99 | (0.59 - 1.37) |  |  | 2.13 | (1.46 - 2.92) | + |
| 4 (-) | 2.18 | (0.78 - 3.46) |  |  | 2.92 | (2.01 - 3.94) | + |  | 3.37 | (2.14 - 4.58) | + |
| 6 (-) | 2.92 | (0.70 - 4.56) |  |  | 2.67 | (1.85 - 3.68) | + |  | 1.82 | (1.17 - 2.55) | + |
| 12 (-) | 0.83 | (0.23 - 1.52) |  |  | 1.52 | (1.05 - 2.09) | + |  | 1.31 | (0.89 - 1.79) |  |
| 13 (-) | 0.36 | (0.15 - 0.63) |  |  | 3.27 | (2.31 - 4.57) | + |  | 3.02 | (2.07 - 4.18) | + |
| 15 (-) | 3.91 | (1.11 - 5.94) | + |  | 0.78 | (0.52 - 1.06) |  |  | 1.94 | (1.29 - 2.72) | + |
| 16 (-) | 0.39 | (0.20 - 0.61) |  |  | 3.23 | (2.19 - 4.51) | + |  | 1.09 | (0.69 - 1.52) |  |
| 35 (45) | 2.14 | (1.46 - 2.93) | + |  | 3.26 | (2.10 - 4.50) | + |  | 6.97 | (4.51 - 9.79) | + |
| 38 (48) | 2.62 | (1.79 - 3.65) | + |  | 0.27 | (0.18 - 0.37) |  |  | 0.24 | (0.15 - 0.32) |  |
| 45 (-) | 1.11 | (0.76 - 1.53) |  |  | 4.37 | (2.99 - 6.17) | + |  | 2.84 | (1.84 - 3.90) | + |
| 48 (57) | 1.42 | (0.98 - 1.96) |  |  | 2.49 | (1.59 - 3.38) | + |  | 1.95 | (1.28 - 2.66) | + |
| 69 (78) | 0.22 | (0.15 - 0.30) |  |  | 1.19 | (0.81 - 1.62) |  |  | 1.67 | (1.15 - 2.42) | + |
| 73 (82) | 0.02 | (0.01 - 0.03) |  |  | 1.72 | (1.16 - 2.35) | + |  | 1.63 | (1.12 - 2.23) | + |
| 74 (-) | 0.13 | (0.09 - 0.18) |  |  | 2.59 | (1.66 - 3.63) | + |  | 1.56 | (1.06 - 2.13) | + |
| 84 (92) | 7.15 | (4.83 - 9.95) | + |  | 3.51 | (2.33 - 4.79) | + |  | 2.53 | (1.70 - 3.45) | + |
| 86 (93) | 8.18 | (5.44-11.29) | + |  | 0.53 | (0.35 - 0.72) |  |  | 0.42 | (0.28 - 0.56) |  |
| 97 (104) | 3.14 | (2.06 - 4.33) | + |  | 1.25 | (0.82 - 1.73) |  |  | 1.24 | (0.78 - 1.70) |  |
| 120 (-) | 0.22 | (0.15 - 0.30) |  |  | 1.70 | (1.10 - 2.38) | + |  | 3.39 | (2.28 - 4.64) | + |
| 125 (129) | 2.21 | (1.38 - 3.24) | + |  | 1.20 | (0.81 - 1.65) |  |  | 0.80 | (0.53 - 1.08) |  |
| 129 (133) | 2.02 | (1.35 - 2.85) | + |  | 2.85 | (1.83 - 3.95) | + |  | 2.41 | (1.48 - 3.29) | + |
| 137 (140) | 0.20 | (0.14 - 0.28) |  |  | 1.16 | (0.79 - 1.57) |  |  | 1.74 | (1.13 - 2.33) | + |
| 139 (142) | 0.51 | (0.35 - 0.71) |  |  | 1.95 | (1.27 - 2.66) | + |  | 4.34 | (2.93 - 6.10) | + |
| 141 (144) | 0.16 | (0.11 - 0.22) |  |  | 1.69 | (1.11 - 2.31) | + |  | 2.32 | (1.54 - 3.31) | + |
| 143 (146) | 2.02 | (1.34 - 2.74) | + |  | 1.32 | (0.88 - 1.83) |  |  | 1.12 | (0.71 - 1.51) |  |
| 156 (159) | 0.05 | (0.03 - 0.08) |  |  | 1.67 | (1.11 - 2.31) | + |  | 1.04 | (0.73 - 1.42) |  |
| 162 (165) | 2.66 | (1.81 - 3.68) | + |  | 0.84 | (0.56 - 1.12) |  |  | 1.34 | (0.91 - 1.84) |  |
| 163 (166) | 3.13 | (2.03 - 4.43) | + |  | 2.62 | (1.79 - 3.68) | + |  | 4.54 | (2.98 - 6.38) | + |
| 185 (188) | 4.00 | (2.55 - 5.55) | + |  | 1.13 | (0.76 - 1.54) |  |  | 2.14 | (1.41 - 2.94) | + |
| 186 (189) | 2.65 | (1.82 - 3.72) | + |  | 1.47 | (0.93 - 1.97) |  |  | 1.44 | (0.97 - 1.94) |  |
| 191 (194) | 0.60 | (0.40 - 0.82) |  |  | 1.63 | (1.05 - 2.24) | + |  | 0.89 | (0.60 - 1.21) |  |
| 203 (206) | 1.63 | (1.12 - 2.30) | + |  | 0.28 | (0.18 - 0.39) |  |  | 0.36 | (0.23 - 0.51) |  |
| 205 (208) | 2.02 | (1.36 - 2.75) | + |  | 2.37 | (1.59 - 3.25) | + |  | 2.06 | (1.32 - 2.85) | + |
| 215 (218) | 1.42 | (0.97 - 1.94) |  |  | 3.84 | (2.51 - 5.20) | + |  | 2.09 | (1.33 - 2.82) | + |
| 216 (219) | 2.03 | (1.37 - 2.80) | + |  | 2.28 | (1.57 - 3.15) | + |  | 3.64 | (2.44 - 5.07) | + |
| 222 (225) | 11.11 | (7.53-15.29) | + |  | 4.32 | (2.87 - 5.85) | + |  | 3.42 | (2.30 - 4.81) | + |
| 223 (226) | 0.41 | (0.29 - 0.57) |  |  | 4.07 | (2.58 - 5.53) | + |  | 5.14 | (3.36 - 7.24) | + |
| 227 (230) | 0.21 | (0.14 - 0.28) |  |  | 1.65 | (1.12 - 2.30) | + |  | 1.45 | (0.98 - 1.98) |  |
| 249 (252) | 3.23 | (2.14 - 4.38) | + |  | 0.22 | (0.14 - 0.30) |  |  | 0.24 | (0.17 - 0.34) |  |
| 256 (259) | 4.47 | (3.03 - 6.04) | + |  | 1.08 | (0.75 - 1.48) |  |  | 1.16 | (0.74 - 1.58) |  |
| 257 (260) | 0.81 | (0.56 - 1.10) |  |  | 3.73 | (2.55 - 5.07) | + |  | 5.06 | (3.61 - 7.12) | + |
| 260 (-) | 0.54 | (0.34 - 0.74) |  |  | 2.00 | (1.29 - 2.72) | + |  | 1.78 | (1.15 - 2.42) | + |
| 261 (263) | 2.04 | (1.33 - 2.79) | + |  | 1.07 | (0.70 - 1.45) |  |  | 1.22 | (0.84 - 1.70) |  |
| 286 (288) | 2.66 | (1.74 - 3.60) | + |  | 2.43 | (1.70 - 3.32) | + |  | 5.51 | (3.69 - 7.31) | + |
| 295 (297) | 3.02 | (1.93 - 4.31) | + |  | 1.95 | (1.29 - 2.66) | + |  | 2.95 | (2.04 - 4.02) | + |
| 365 (367) | 2.64 | (1.80 - 3.57) | + |  | 0.13 | (0.08 - 0.17) |  |  | 0.25 | (0.17 - 0.34) |  |
| 370 (372) | 0.07 | (0.05 - 0.10) |  |  | 1.87 | (1.24 - 2.56) | + |  | 2.05 | (1.37 - 2.95) | + |
| 372 (374) | 2.06 | (1.32 - 2.90) | + |  | 0.41 | (0.26 - 0.58) |  |  | 0.66 | (0.44 - 0.91) |  |
| 408 (410) | 2.03 | (1.35 - 2.74) | + |  | 0.09 | (0.06 - 0.12) |  |  | 0.06 | (0.04 - 0.09) |  |
| 451 (453) | 4.28 | (2.76 - 6.03) | + |  | 1.22 | (0.84 - 1.66) |  |  | 0.84 | (0.56 - 1.13) |  |
| 460 (462) | 0.82 | (0.56 - 1.17) |  |  | 3.14 | (2.13 - 4.44) | + |  | 2.96 | (1.86 - 4.13) | + |
| 499 (501) | 3.90 | (2.53 - 5.32) | + |  | 1.93 | (1.32 - 2.62) | + |  | 1.91 | (1.29 - 2.61) | + |
| 520 (521) | 1.89 | (1.28 - 2.61) | + |  | 2.21 | (1.53 - 3.09) | + |  | 1.67 | (1.13 - 2.28) | + |
| 527 (528) | 2.03 | (1.41 - 2.84) | + |  | 0.81 | (0.56 - 1.15) |  |  | 1.32 | (0.85 - 1.83) |  |
| 533 (534) | 2.03 | (1.36 - 2.80) | + |  | 1.89 | (1.30 - 2.66) | + |  | 2.24 | (1.46 - 3.07) | + |
| 547 (548) | 0.35 | (0.22 - 0.50) |  |  | 2.61 | (1.79 - 3.59) | + |  | 2.15 | (1.40 - 2.89) | + |
| The dash ‘(-)’ indicates no equivalent position in the H3 reference genome used by the FluDB HA Subtype Numbering system. | | | | | | | | | | | |

Table S6: Codon sites under pervasive positive selection as identified using Bayesian Renaissance Counting (BRC), two-rate fixed effects likelihood (FEL) and single-likelihood ancestor counting (SLAC) in India.

| **H1 Site (H3#)^a^** | **Renaissance Counting** | |  | **FEL** | |  | **SLAC** | |
| --- | --- | --- | --- | --- | --- | --- | --- | --- |
|  | **d_N_/d_S_** | **95% BCI** |  | **d_N_/d_S_** | **P-value** |  | **d_N_/d_S_** | **P-value** |
| S84 (92) | 8.14 | (5.68 - 10.93) |  | ∞ | 0.02 |  | ∞ | 0.03 |
| K163 (166) | 3.83 | (2.69 - 5.36) |  | 5.05 | 0.05 |  | 5.40 | 0.05 |
| S185 (188) | 4.50 | (3.19 - 6.21) |  | ∞ | 0.03 |  | ∞ | 0.06 |
| A186 (189) | 3.35 | (2.27 - 4.52) |  | ∞ | 0.05 |  | ∞ | 0.09 |
| D222 (225) | 13.43 | (9.45 - 18.42) |  | ∞ | < 0.01 |  | ∞ | < 0.01 |
| A256 (259) | 4.40 | (3.12 - 6.10) |  | ∞ | 0.02 |  | ∞ | 0.04 |
| ^a^ Relative H3 numbering determined by FluDB HA Subtype Numbering algorithm; Results presented as the mean dN/dS and range of both samples. The dash ‘(-)’ indicates no equivalent position in the H3 reference genome. Bayesian Credible Interval (BCI). | | | | | | | | |

**
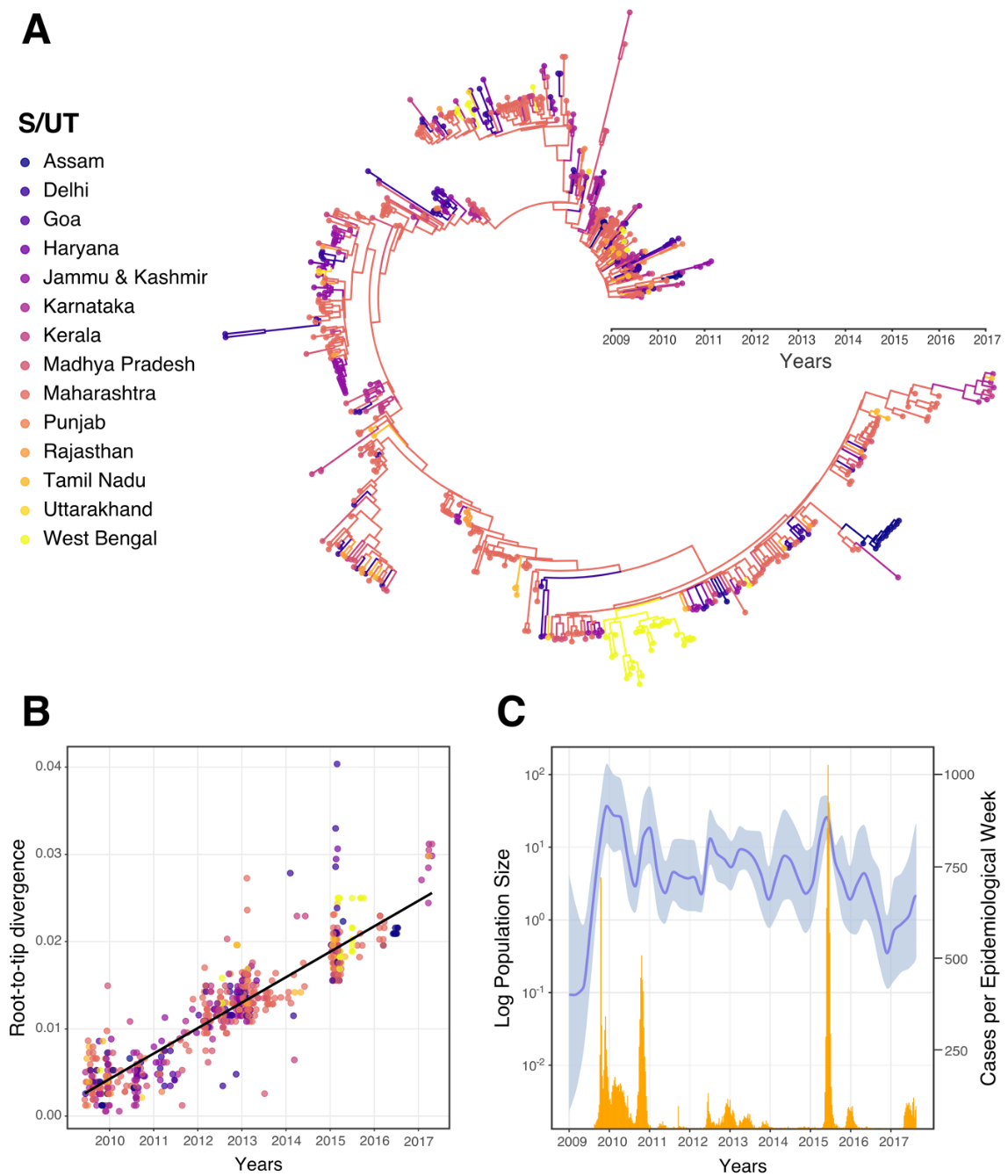
**

**Figure S3:** Maximum clade credibility tree (MCC) of 613 A/H1N1pdm09 taxa sequenced for HA between 2009 and 2017 inclusive from 14 states and union territory’s (S/UT) in India. Tips are coloured by sampled location state and internal edges and nodes coloured by statistically inferred ancestral origin.


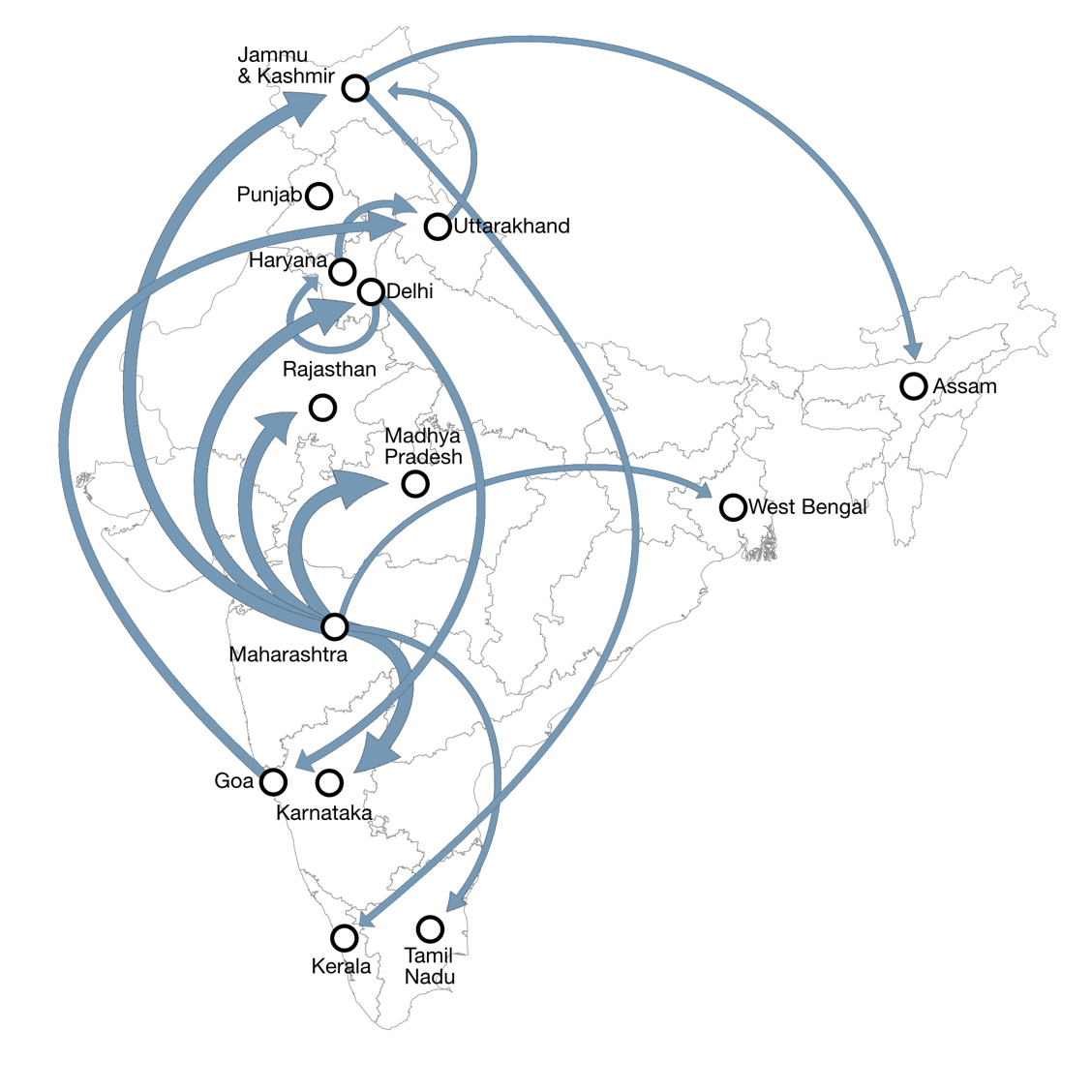


**Figure S4:** Definitively supported (BF > 100) routes of A/H1N1pdm09 transmission between S/UT in India from 2009 to 2017 based on five randomly subsampled datasets. Direction of spread is indicated by arrowheads. Paths between S/UT are weighted according to their corresponding significance in Supplementary Table S7 on a log scale. Less supported routes (100 > BF > 3) are not shown but can be seen in Supplementary Table S7.

Table S7: Statistically supported routes for A/H1N1pdm09 transmission in India between 14 S/UT from 2009 to 2017 based on the average of five randomly subsampled sequence datasets (N=259).

| **Origin** | **Destination** | **Bayes Factor (BF)** | **Posterior Probability** |
| --- | --- | --- | --- |
| Maharashtra | Karnataka | 88654.23 | 1.00 |
| Maharashtra | Madhya Pradesh | 88653.09 | 1.00 |
| Maharashtra | Rajasthan | 88504.03 | 0.95 |
| Maharashtra | Delhi | 67205.70 | 0.97 |
| Maharashtra | Jammu Kashmir | 45396.62 | 0.98 |
| Goa | Uttarakhand | 3732.35 | 0.99 |
| Delhi | Goa | 1390.98 | 0.98 |
| Haryana | Uttarakhand | 564.42 | 0.94 |
| Jammu Kashmir | Kerala | 394.76 | 0.77 |
| Delhi | Haryana | 362.22 | 0.88 |
| Maharashtra | Tamil Nadu | 301.75 | 0.89 |
| Jammu Kashmir | Assam | 205.30 | 0.66 |
| Maharashtra | West Bengal | 181.67 | 0.76 |
| Uttarakhand | Jammu Kashmir | 177.89 | 0.85 |
| Maharashtra | Kerala | 73.90 | 0.72 |
| Delhi | Punjab | 62.11 | 0.48 |
| Karnataka | Kerala | 46.67 | 0.74 |
| Maharashtra | Assam | 38.60 | 0.52 |
| Jammu Kashmir | Punjab | 20.31 | 0.48 |
| Karnataka | Tamil Nadu | 16.23 | 0.53 |
| Jammu Kashmir | Delhi | 9.79 | 0.23 |
| Tamil Nadu | Maharashtra | 8.18 | 0.34 |
| Karnataka | West Bengal | 5.67 | 0.22 |
| Madhya Pradesh | Kerala | 5.35 | 0.23 |
| Jammu Kashmir | Rajasthan | 4.95 | 0.24 |
| West Bengal | Karnataka | 4.80 | 0.23 |
| Rajasthan | Madhya Pradesh | 4.19 | 0.25 |
| Punjab | Haryana | 3.81 | 0.22 |

Table S8: Statistically supported routes for A/H1N1pdm09 transmission in India between 14 S/UT discrete locations from 2009 to 2017.

| **Origin** | **Destination** | **Bayes Factor (BF)** | **Posterior Probability** |
| --- | --- | --- | --- |
| Maharashtra | Rajasthan | 221243.76 | 1.00 |
| Maharashtra | Tamil Nadu | 221243.76 | 1.00 |
| Maharashtra | Delhi | 221243.76 | 1.00 |
| Maharashtra | Jammu & Kashmir | 221243.76 | 1.00 |
| Maharashtra | Karnataka | 221243.76 | 1.00 |
| Maharashtra | Madhya Pradesh | 221243.76 | 1.00 |
| Maharashtra | West Bengal | 27644.71 | 1.00 |
| Delhi | Goa | 656.16 | 0.98 |
| Delhi | Haryana | 340.59 | 0.97 |
| Jammu & Kashmir | Punjab | 258.86 | 0.95 |
| Haryana | Uttarakhand | 252.69 | 0.95 |
| Maharashtra | Assam | 239.42 | 0.95 |
| Goa | Uttarakhand | 227.68 | 0.95 |
| Maharashtra | Kerala | 199.23 | 0.94 |
| Karnataka | Kerala | 132.99 | 0.92 |
| Uttarakhand | Jammu & Kashmir | 126.78 | 0.91 |
| Madhya Pradesh | Kerala | 114.94 | 0.90 |
| Karnataka | Tamil Nadu | 28.35 | 0.70 |
| Karnataka | Madhya Pradesh | 19.70 | 0.62 |
| West Bengal | Karnataka | 8.30 | 0.40 |
| Jammu & Kashmir | Kerala | 6.24 | 0.34 |
| Karnataka | Haryana | 5.59 | 0.31 |
| Karnataka | Goa | 5.21 | 0.30 |
| Karnataka | Punjab | 4.50 | 0.27 |
| Karnataka | Jammu & Kashmir | 4.40 | 0.26 |
| Jammu & Kashmir | Assam | 4.40 | 0.26 |
| Karnataka | Maharashtra | 3.70 | 0.23 |
| Tamil Nadu | Kerala | 3.45 | 0.22 |
| Kerala | Punjab | 3.20 | 0.21 |
| Jammu & Kashmir | Karnataka | 3.00 | 0.20 |

Table S9. Distribution of A/H1N1pdm09 viruses belonging to clade 6B by year and S/UT in India between 2009 and 2011.

|  | **Year** | | | | | | | |  |  |  | |
| --- | --- | --- | --- | --- | --- | --- | --- | --- | --- | --- | --- | --- |
| **S/UT (Population 10^6^)** | **2009** | **2010** | **2011** | **2012** | **2013** | **2014** | **2015** | **2016** | **2017** |  | **Total** | **%** |
| Assam (31.2) |  |  |  |  |  |  | 1 |  |  |  | 1 | 0.45 |
| Delhi (16.8) |  |  |  |  | 1 | 2 | 17 |  |  |  | 20 | 9.05 |
| Goa (1.5) |  |  |  |  |  |  |  |  |  |  |  | 0.00 |
| Haryana (25.4) |  |  |  |  |  |  | 1 |  |  |  | 1 | 0.45 |
| Jammu & Kashmir (12.5) |  |  |  |  |  | 3 |  | 11 |  |  | 14 | 6.33 |
| Karnataka (61.1) |  |  |  |  |  |  |  |  | 6 |  | 6 | 2.71 |
| Kerala (33.4) |  |  |  |  |  | 2 | 3 |  | 2 |  | 7 | 3.17 |
| Madhya Pradesh (72.6) |  |  |  |  | 1 |  | 17 |  |  |  | 18 | 8.14 |
| Maharashtra (112.4) |  |  |  | 2 | 39 | 6 | 53 | 8 |  |  | 108 | 48.87 |
| Punjab (27.7) |  |  |  |  |  |  |  |  |  |  |  | 0.00 |
| Rajasthan (68.6) |  |  |  |  | 3 |  | 5 |  |  |  | 8 | 3.62 |
| Tamil Nadu (72.15) |  |  |  | 2 |  | 2 | 6 | 1 |  |  | 11 | 4.98 |
| Uttarakhand (10.1) |  |  |  |  |  |  | 1 |  |  |  | 1 | 0.45 |
| West Bengal (91.3) |  |  |  |  |  |  | 26 |  |  |  | 26 | 11.76 |
| Year Total |  |  |  | 4 | 47 | 12 | 141 | 8 | 9 |  | 221 | 100.00 |
